# Fast score test with global null estimation regardless of missing genotypes

**DOI:** 10.1371/journal.pone.0199692

**Published:** 2018-07-05

**Authors:** Shuntaro Sato, Masao Ueki, Alzheimer’s Disease Neuroimaging Initiative

**Affiliations:** 1 Clinical Research Center, Nagasaki University Hospital, 1-7-1 Sakamoto, Nagasaki, Nagasaki 852-8501, Japan; 2 Biostatistics, Graduate School of Medicine, Kurume University, 67 Asahi-machi, Kurume, Fukuoka, 830-0011, Japan; 3 Statistical Genetics Team, RIKEN Center for Advanced Intelligence Project, 1-4-1 Nihonbashi, Chuo-ku, Tokyo, 103-0027, Japan; Janssen Research and Development, UNITED STATES

## Abstract

In genome-wide association studies (GWASs) for binary traits (or case-control samples) in the presence of covariates to be adjusted for, researchers often use a logistic regression model to test variants for disease association. Popular tests include Wald, likelihood ratio, and score tests. For likelihood ratio test and Wald test, maximum likelihood estimation (MLE), which requires iterative procedure, must be computed for each single nucleotide polymorphism (SNP). In contrast, the score test only requires MLE under the null model, being lower in computational cost than other tests. Usually, genotype data include missing genotypes because of assay failures. It loses computational efficiency in the conventional score test (CST), which requires null estimation by excluding individuals with missing genotype for each SNP. In this study, we propose two new score tests, called PM1 and PM2, that use a single global null estimator for all SNPs regardless of missing genotypes, thereby enabling faster computation than CST. We prove that PM2 and CST have an equivalent asymptotic power and that the power of PM1 is asymptotically lower than that of PM2. We evaluate the performance of the proposed methods in terms of type I error rates and power by simulation studies and application to real GWAS data provided by the Alzheimer’s Disease Neuroimaging Initiative (ADNI), confirming our theoretical results. ADNI-GWAS application demonstrated that the proposed score tests improve computational speed about 6–18 times faster than the existing tests, CST, Wald tests and likelihood ratio tests. Our score tests are general and applicable to other regression models.

## Introduction

Over the last decades, genome-wide association studies (GWASs) have successfully identified many variants that are susceptible to hundreds of human diseases and traits [1, 2]. For discovery of an association between disease and genotypes, researchers often use tests based on a logistic regression model. It can analyze an association between disease (binary trait) and each single nucleotide polymorphism (SNP) while adjusting for the effect of covariates including age, sex, body mass index, and/or principal components for population stratification [3]. Wald test, likelihood ratio test, and score test are popularly used to examine the effects of each SNP on an outcome and are applied to genome-wide scan. For example, PLINK (http://zzz.bwh.harvard.edu/plink/) [4] and PLINK 1.9 (https://www.cog-genomics.org/plink2/) [5] use the Wald test by default for genome-wide scan in the presence of covariates to be adjusted for. Recently, we need to test over 500,000 loci in SNP-GWAS or tens of millions of loci in the whole-genome sequencing studies. Inclusion of large number of covariates slows the computation further. We often carry out this genome-wide scan for multiple traits. Consequently, computationally efficient method for genome-wide scan for large number of variants is highly desired [6].

In a logistic regression model, an iterative procedure such as Newton–Raphson method is needed to compute maximum likelihood estimator (MLE), which incurs computational burden in application to genome-wide scan. For the Wald test and the likelihood ratio test, MLE under full model for each SNP is required. On the other hand, the score test only requires MLE under null model. Furthermore, since the null model is common for all SNPs in testing association of SNPs (i.e. no SNPs have effect on outcome), if no SNPs have missing genotypes, a single null estimation can be used in score test statistics for all SNPs and computationally demanding iterative optimization process in computing MLE for each SNP is unnecessary. However, genotype data usually include missing genotypes because of assay failures [7]. Then, we still face computational burden even in the score test because missing pattern differs across loci and null estimation by excluding individuals with missing separately for each SNP is necessary. For example, the qtscore function in GenABEL package [8] implements fast genome-wide scan by the score test, where individuals with missing genotpyes are removed for each SNP [9, 10]. The Wald test implemented in PLINK also uses the complete case analysis.

**Fig 1:**
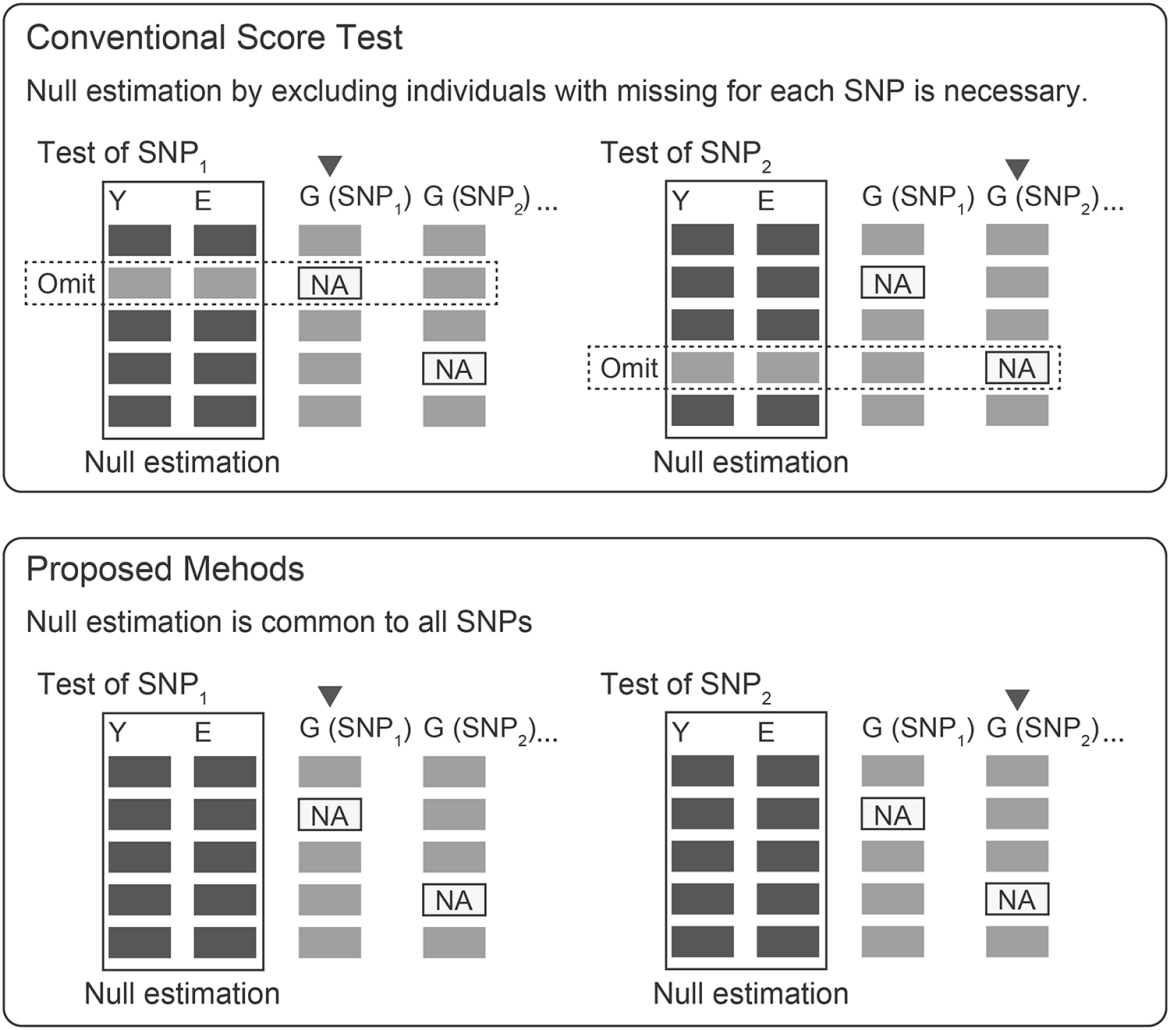
Conceptual difference between the conventional score test and the proposed new score tests. Conceptual difference between the conventional score test (CST) and the proposed new score tests (PM1 and PM2). Outcomes (Y) and covariates (E) are observed in all individuals. NA indicates missing genotype. In CST, null estimation is performed for each SNP excluding individuals with missing genotype. On the other hand, null estimation required in PM1 or PM2 is common in all SNP.

In this study, we propose two fast score tests, called the proposed method 1 (PM1) and the proposed method 2 (PM2), that require only a single global null estimator for all SNPs regardless of missing genotypes unlike the conventional score test (CST) which requires separate null estimations for all SNPs. Fig 1 illustrates our idea. We prove that PM2 and CST have an equivalent asymptotic power and that the power of PM1 is asymptotically lower than that of PM2. We show through simulation studies that our PM1 and PM2 give correct control of the type I error. The simulations also confirm our theoretical results for an equivalent power between PM2 and CST, and the lower power of PM1 although the loss of power seems to be small in a range of practical missing genotype rates (<10%) in current GWAS. Application to real GWAS data from the Alzheimer’s Disease Neuroimaging Initiative (ADNI) demonstrates 6–18 times faster computation of the proposed methods than the CST, Wald test, and likelihood ratio test.

## Materials and methods

### Logistic regression model

We consider a case-control study with total sample size *n*. For individual *i*, let *Y*_*i*_ = 1 or *Y*_*i*_ = 0 be an indicator of disease (case or control), respectively. The probability of being case is *π*_*i*_ = *Pr*(*Y*_*i*_ = 1). Our logistic regression model for a SNP is written as

$$\begin{array}{c} \text{logit}\left[Pr\left(Y_{i}=1\right)\right]=\text{logit}\left[\pi_{i}\left(\beta_{0},\beta_{e},\beta_{g}\right)\right]=\beta_{0}+\beta_{e}E_{i}+\beta_{g}G_{i} \end{array}$$ (1)

where *G*_*i*_ is some genotype coding, such as an additive coding {0, 1, 2} and *E*_*i*_ is a covariate or an environment factor. Letting *θ*_1_ = (*β*_0_, *β*_*e*_)^T^, *θ*_2_ = *β*_*g*_, $\theta ={\left(\theta_{1}^{T},\theta_{2}^{T}\right)}^{T}$, *X*_1_ = (1, *E*), and *X*_2_ = *G*, the probability of being case at a full model is

$$\begin{array}{c} \pi_{i}\left(\theta \right)=\pi_{i}\left(\theta_{1},\theta_{2}\right)=\frac{\exp(\beta_{0}+\beta_{e}E_{i}+\beta_{g}G_{i})}{1+\exp(\beta_{0}+\beta_{e}E_{i}+\beta_{g}G_{i})}=\frac{\exp(X_{1}\theta_{1}+X_{2}\theta_{2})}{1+\exp(X_{1}\theta_{1}+X_{2}\theta_{2})}. \end{array}$$

Under these setting, the log-likelihood function at the full model is

$$\begin{array}{c} \log f\left(\theta \right)=\sum_{i=1}^{n}\left[Y_{i}\log\pi_{i}\left(\theta \right)+\left(1-Y_{i}\right)\log\left\{1-\pi_{i}\left(\theta \right)\right\}\right]. \end{array}$$

For logistic regression model, no closed-form solution is available for MLE for ${\left(\theta_{1}^{T},\theta_{2}^{T}\right)}^{T}$, and iterative procedure such as Newton–Raphson method is required, which causes high computational load in applying to genome-wide scan.

The score function evaluated at the full model is

$$\begin{array}{c} \left(\begin{array}{c} u_{1}\left(\theta \right)\\ u_{2}\left(\theta \right) \end{array})=(\begin{array}{c} \partial \log f\left(\theta \right)/\partial \theta_{1}\\ \partial \log f\left(\theta \right)/\partial \theta_{2} \end{array})=(\begin{array}{c} \sum_{i=1}^{n}u_{1i}\left(\theta \right)\\ \sum_{i=1}^{n}u_{2i}\left(\theta \right) \end{array}\right) \end{array}$$

and their covariance matrix at the full model is

$$\begin{array}{cc} J_{11}\left(\theta \right)=(\begin{array}{cc} J_{11}\left[1,1\right]\left(\theta \right) & J_{11}\left[1,2\right]\left(\theta \right)\\ J_{11}\left[2,1\right]\left(\theta \right) & J_{11}\left[2,2\right]\left(\theta \right) \end{array}) & =-\frac{1}{n}(\begin{array}{cc} \partial u_{1}\left(\theta \right)/\partial \theta_{1} & \partial u_{1}\left(\theta \right)/\partial \theta_{2}\\ \partial u_{2}\left(\theta \right)/\partial \theta_{1} & \partial u_{2}\left(\theta \right)/\partial \theta_{2} \end{array}). \end{array}$$

Here, the score function and their covariance matrix at a null model ($\theta ={\left(\theta_{1}^{T},0^{T}\right)}^{T}$) are

$$\begin{array}{cc} \left(\begin{array}{c} u_{1}\left(\theta_{1}\right)\\ u_{2}\left(\theta_{1}\right) \end{array}\right) & =(\begin{array}{c} u_{1}\left(\theta \right)\\ u_{2}\left(\theta \right) \end{array}){|}_{\theta ={\left(\theta_{1}^{T},0^{T}\right)}^{T}}\\ & =(\begin{array}{c} \partial \log f\left(\theta \right)/\partial \theta_{1}\\ \partial \log f\left(\theta \right)/\partial \theta_{2} \end{array}){|}_{\theta ={\left(\theta_{1}^{T},0^{T}\right)}^{T}}=(\begin{array}{c} \sum_{i=1}^{n}u_{1i}\left(\theta \right)\\ \sum_{i=1}^{n}u_{2i}\left(\theta \right) \end{array}){|}_{\theta ={\left(\theta_{1}^{T},0^{T}\right)}^{T}} \end{array}$$

and

$$\begin{array}{cc} \left(\begin{array}{cc} J_{11}\left[1,1\right]\left(\theta_{1}\right) & J_{11}\left[1,2\right]\left(\theta_{1}\right)\\ J_{11}\left[2,1\right]\left(\theta_{1}\right) & J_{11}\left[2,2\right]\left(\theta_{1}\right) \end{array}\right) & =(\begin{array}{cc} J_{11}\left[1,1\right]\left(\theta \right) & J_{11}\left[1,2\right]\left(\theta \right)\\ J_{11}\left[2,1\right]\left(\theta \right) & J_{11}\left[2,2\right]\left(\theta \right) \end{array}){|}_{\theta ={\left(\theta_{1}^{T},0^{T}\right)}^{T}}\\ & =-\frac{1}{n}(\begin{array}{cc} \partial u_{1}\left(\theta \right)/\partial \theta_{1} & \partial u_{1}\left(\theta \right)/\partial \theta_{2}\\ \partial u_{2}\left(\theta \right)/\partial \theta_{1} & \partial u_{2}\left(\theta \right)/\partial \theta_{2} \end{array}){|}_{\theta ={\left(\theta_{1}^{T},0^{T}\right)}^{T}}. \end{array}$$

### Wald, likelihood ratio, and score tests

In GWAS, the null hypothesis *H*_0_: *θ*_2_ = 0 in the logistic regression model (1) for each SNP is tested using the Wald test, the likelihood ratio test, or the score test. The Wald statistic is

$$\begin{array}{c} W={\hat{\theta }}_{2}^{\text{T}}var{\left({\hat{\theta }}_{2}\right)}^{-1}{\hat{\theta }}_{2}, \end{array}$$

where ${\hat{\theta }}_{2}$ is MLE for *θ*_2_ under the full model and $var({\hat{\theta }}_{2})$ denotes an estimator of (asymptotic) variance of ${\hat{\theta }}_{2}$. Wald tests need single optimization for full model MLE for each SNP. The likelihood ratio statistic is

$$\begin{array}{c} LR=-2\{\log f\left({\overset{ˇ}{\theta }}_{1},0\right)-\log f\left({\hat{\theta }}_{1},{\hat{\theta }}_{2}\right)\}, \end{array}$$

where ${\hat{\theta }}_{1}$ and ${\hat{\theta }}_{2}$ are MLE for *θ*_1_ and *θ*_2_, respectively, under the full model and ${\overset{ˇ}{\theta }}_{1}$ is MLE for *θ*_1_ under the null model. Likelihood ratio tests need two optimizations for the null model MLE and the full model MLE for each SNP. The score statistic is

$$\begin{array}{c} S=u_{2}{\left({\overset{ˇ}{\theta }}_{1}\right)}^{T}{var\{u_{2}\left({\overset{ˇ}{\theta }}_{1}\right)\}}^{-1}u_{2}\left({\overset{ˇ}{\theta }}_{1}\right), \end{array}$$

where $var\{u_{2}\left({\overset{ˇ}{\theta }}_{1}\right)\}$ denotes an estimator of (asymptotic) variance of $u_{2}\left({\overset{ˇ}{\theta }}_{1}\right)$. Score tests require single optimization for the null model MLE for each SNP. If there is no missing genotypes for all SNPs, that is complete data, then null estimation is common for all SNPs. We focus score tests in this study, because parameter estimation which needs iterative optimization can be performed only once. Therefore, the score test with complete data can be computed with much lower computational cost than the Wald and the Likelihood ratio tests.

For a SNP, which we denote the individual *i*’s genotype by *G*_*i*_, we consider the setting where there are missing genotypes. Under this setting, score functions and their covariance matrix at the null model are given as follows:

$$\begin{array}{c} (\begin{array}{c} u_{1}^{m}\left(\theta_{1}\right)\\ u_{2}^{m}\left(\theta_{1}\right) \end{array})=(\begin{array}{c} u_{1}^{m}\left(\theta \right)\\ u_{2}^{m}\left(\theta \right) \end{array}){|}_{\theta ={\left(\theta_{1}^{T},0^{T}\right)}^{T}}=(\begin{array}{c} \sum_{i=1}^{n}u_{1i}\left(\theta \right)I_{i}\\ \sum_{i=1}^{n}u_{2i}\left(\theta \right)I_{i} \end{array}){|}_{\theta ={\left(\theta_{1}^{T},0^{T}\right)}^{T}} \end{array}$$

and

$$\begin{array}{cc} \left(\begin{array}{cc} J_{11}^{m}\left[1,1\right]\left(\theta_{1}\right) & J_{11}^{m}\left[1,2\right]\left(\theta_{1}\right)\\ J_{11}^{m}\left[2,1\right]\left(\theta_{1}\right) & J_{11}^{m}\left[2,2\right]\left(\theta_{1}\right) \end{array}\right) & =(\begin{array}{cc} J_{11}^{m}\left[1,1\right]\left(\theta \right) & J_{11}^{m}\left[1,2\right]\left(\theta \right)\\ J_{11}^{m}\left[2,1\right]\left(\theta \right) & J_{11}^{m}\left[2,2\right]\left(\theta \right) \end{array}){|}_{\theta ={\left(\theta_{1}^{T},0^{T}\right)}^{T}}\\ & =-\frac{1}{n}(\begin{array}{cc} \partial u_{1}^{m}\left(\theta \right)/\partial \theta_{1} & \partial u_{1}^{m}\left(\theta \right)/\partial \theta_{2}\\ \partial u_{2}^{m}\left(\theta \right)/\partial \theta_{1} & \partial u_{2}^{m}\left(\theta \right)/\partial \theta_{2} \end{array}){|}_{\theta ={\left(\theta_{1}^{T},0^{T}\right)}^{T}}. \end{array}$$

where *I*_*i*_ is an indicator defined by

$$\begin{array}{c} I_{i}=\{\begin{array}{cc} 1 & \text{if}\;G_{i}\;\text{is}\;\text{observed}\\ 0 & \text{if}\;G_{i}\;\text{is}\;\text{missing} \end{array}. \end{array}$$

For theoretical studies in what follows, we assume missing completely at random (MCAR), that is, *I*_*i*_ independently and identically follows a binomial distribution of size 1, *I*_*i*_ ∼ Bin(1, 1 − *R*) for *i* = 1, ⋯, *n*, where *R* is a probability of random missing, and is independent of *u*_1*i*_ and *u*_2*i*_.

### Score tests

#### Conventional score test

The conventional score test (CST) means the score test using null estimator computed by removing individuals having missing genotype to be tested. The score statistic of CST is expressed by the following formula, and asymptotically follows a chi-squared distribution with 1 degree of freedom under the null hypothesis:

$$\begin{array}{c} S_{\text{CST}}={\left\{\frac{1}{\sqrt{n}}\sum_{i=1}^{n}u_{2i}^{m}\left({\overset{ˇ}{\theta }}_{1}^{m}\right)\right\}}^{T}V_{m}^{-1}\{\frac{1}{\sqrt{n}}\sum_{i=1}^{n}u_{2i}^{m}\left({\overset{ˇ}{\theta }}_{1}^{m}\right)\} \end{array}$$

where ${\overset{ˇ}{\theta }}_{1}^{m}$ is MLE for *θ*_1_ under the null model on CST and $V_{m}=\left\{-J_{11}^{m}\left[2,1\right]\left({\overset{ˇ}{\theta }}_{1}^{m}\right)J_{11}^{m}\left[1,1\right]{\left({\overset{ˇ}{\theta }}_{1}^{m}\right)}^{-1}J_{11}^{m}\left[1,2\right]\left({\overset{ˇ}{\theta }}_{1}^{m}\right)+J_{11}^{m}\left[2,2\right]\left({\overset{ˇ}{\theta }}_{1}^{m}\right)\right\}/n$. In Appendix (p. 8), it is shown that the convergence rate to chi-square distribution has order *o*_*p*_(1) as *n* → ∞.

#### Proposed methods

We describe two new proposed score tests. The proposed method 1 (PM1) is the score test which uses a single null estimator for all score test statistics regardless of missing genotypes. See Fig 1 for the difference from CST. The score statistic of PM1 is expressed by the following formula, and asymptotically follows a chi-squared distribution with 1 degree of freedom under the null hypothesis:

$$\begin{array}{c} S_{\text{PM1}}={\left\{\frac{1}{\sqrt{n}}\sum_{i=1}^{n}u_{2i}^{m}\left({\overset{ˇ}{\theta }}_{1}^{f}\right)\right\}}^{T}V_{f}^{-1}\{\frac{1}{\sqrt{n}}\sum_{i=1}^{n}u_{2i}^{m}\left({\overset{ˇ}{\theta }}_{1}^{f}\right)\} \end{array}$$

where ${\overset{ˇ}{\theta }}_{1}^{f}$ is MLE for *θ*_1_ under the null model computed independently of genotype data using all individuals, and $V_{f}=\left\{-J_{11}^{m}\left[2,1\right]\left({\overset{ˇ}{\theta }}_{1}^{f}\right)J_{11}\left[1,1\right]{\left({\overset{ˇ}{\theta }}_{1}^{f}\right)}^{-1}J_{11}^{m}\left[1,2\right]\left({\overset{ˇ}{\theta }}_{1}^{f}\right)+J_{11}^{m}\left[2,2\right]\left({\overset{ˇ}{\theta }}_{1}^{f}\right)\right\}/n$. In Appendix (p. 10), it is shown that the convergence rate to chi-square distribution has order *o*_*p*_(1) as *n* → ∞. The MLE ${\overset{ˇ}{\theta }}_{1}^{f}$ is a single global null estimator used in all test statistics for genome-wide scan and does not require re-computation for all SNPs unlike CST, and hence, PM1 achieves lower computational cost than CST. However, we showed that the power of PM1 is asymptotically lower than that of CST. The power of the score test statistic asymptotically increases as the non-centrality parameter increases. In Appendix (p.14–17), we have shown that the mean of CST score function is asymptotically equivalent to that of PM1 score function while the variance of PM1 score function is bigger than the variance of CST score function. That is, the magnitude of non-centrality parameter is dominated only by the magnitude of variance, and the non-centrality parameter of PM1 is smaller than that of CST. Therefore, the power of PM1 is smaller than that of CST.

To improve power, we developed a second test, called the proposed method 2 (PM2). Here, we define the following modified score function:

$$\begin{array}{c} u_{2i}^{*}\left(\theta_{1}\right)=u_{2i}^{m}\left(\theta_{1}\right)-J_{11}^{m}\left[2,1\right]\left(\theta_{1}\right)J_{11}^{m}\left[1,1\right]{\left(\theta_{1}\right)}^{-1}u_{1i}^{m}\left(\theta_{1}\right). \end{array}$$ (2)

PM2 uses the above modified score function (2). It is computed without excluding individuals with missing SNP from null model as in PM1. The score statistic of PM2 is expressed by the following formula, and asymptotically follows a chi-squared distribution with 1 degree of freedom under the null hypothesis:

$$\begin{array}{c} S_{\text{PM2}}={\left\{\frac{1}{\sqrt{n}}\sum_{i=1}^{n}u_{2i}^{*}\left({\overset{ˇ}{\theta }}_{1}^{f}\right)\right\}}^{T}V_{m}^{-1}\{\frac{1}{\sqrt{n}}\sum_{i=1}^{n}u_{2i}^{*}\left({\overset{ˇ}{\theta }}_{1}^{f}\right)\}. \end{array}$$

In Appendix (p.13), it is shown that the convergence rate to chi-square distribution has order *o*_*p*_(1) as *n* → ∞. It is shown in Appendix (p.13) that score function $\left(1/\sqrt{n}\right)\sum_{i=1}^{n}u_{2i}^{*}\left({\overset{ˇ}{\theta }}_{1}^{f}\right)$ is asymptotically equivalent to the score function $\left(1/\sqrt{n}\right)\sum_{i=1}^{n}u_{2i}^{m}\left({\overset{ˇ}{\theta }}_{1}^{m}\right)$ of CST. Therefore, the power of PM2 is asymptotically equivalent to CST and higher than that of PM1. In PM2, null estimation is common for all SNPs as in PM1. Thus, PM2 can have lower computational costs than CST.

So far, we have considered the test of *H*_0_: *θ*_2_ = *β*_*g*_ = 0 under the logistic regression model (1). This framework can be easily extended to other tests. For another application, we consider the following logistic regression model involving gene-environment interaction,

$$\begin{array}{c} \text{logit}\left[Pr\left(Y_{i}=1\right)\right]=\beta_{0}+\beta_{e}E_{i}+\beta_{g}G_{i}+\beta_{ge}G_{i}E_{i}. \end{array}$$ (3)

Let *θ*_2_ = (*β*_*g*_, *β*_*ge*_)^T^. We can perform a joint test for combined effect of genetic marginal and of gene-environment interaction [11]. This test constrains *β*_*g*_ = 0 and *β*_*ge*_ = 0 under the null hypothesis, i.e. the degrees of freedom is two. The joint test is more powerful than the test of interaction alone, which is beneficial, particularly for GWAS where marginal SNP effect is low, and it is applied to real data [12].

More details of this section including formulas, derivations, and additional descriptions are given in S1 Appendix. A program code of simulations are given in S2 Appendix and all data files of “Application to ADNI GWAS Data” are freely and publically available from the Alzheimer’s Disease Neuroimaging Initiative (ADNI) database: http://adni.loni.usc.edu/.

## Results

### Evaluation of proposed methods using simulated data

We performed computer simulations to evaluate the performance (type I error rates and power) of the various test statistics described above. We simulated datasets using R [13] based on two logistic regression models ((1) and (3)) assuming disease prevalence 1%. Case-control data was generated accoring to the retrospective sampling as described in [14]. The test corresponding to (1) is called ‘G test’, and the test corresponding to (3) is called ‘G-GE test’. We considered binary variables as a covariate for *E*, e.g. gender, whose population frequency is 50% and set the odds ratio as OR_*e*_ = exp(*β*_*e*_) = 1.2. Missing genotypes were generated assuming missing completely at random, in particular, individuals with missing genotype are randomly assigned with a given missing rate.

#### Type I error rates

We performed 1,000,000 simulation replicates under the null model to estimate type I error rates for a nominal significance threshold of *α* = 5 × 10^−5^. We considered a range of missing rates (2%, 5%, 10%), the number of case or control (1,000, 5,000), and minor allele frequencies (MAF) (10%, 30%).

We provided the estimated type I error rates in various settings in Table 1. For 1,000,000 replication, the standard deviation of the estimated type I error rates is $\sqrt{(0.00005\times 0.99995)/1,000,000}≃0.71\times 10^{-5}$ and the 95% confidence interval is (3.6 × 10^−5^, 6.4 × 10^−5^) for the nominal significance level of *α* = 5 × 10^−5^. From this table, we can see that all of the type I error rates are consistently within the 95% confidence interval, which indicates that the type I error rates are well-controlled at the nominal level.

**Table 1:** Type I error rates of the conventional score test and the proposed methods.

Test	Missing rate (%)	MAF (%)	#case/control	CST	PM1	PM2
G	2	10	1,000	5.0 × 10^−5^	5.5 × 10^−5^	5.0 × 10^−5^
G	2	10	5,000	3.7 × 10^−5^	3.6 × 10^−5^	3.7 × 10^−5^
G	2	30	1,000	5.6 × 10^−5^	4.4 × 10^−5^	5.6 × 10^−5^
G	2	30	5,000	4.3 × 10^−5^	4.6 × 10^−5^	4.3 × 10^−5^
G	5	10	1,000	4.8 × 10^−5^	5.3 × 10^−5^	4.8 × 10^−5^
G	5	10	5,000	4.2 × 10^−5^	3.7 × 10^−5^	4.2 × 10^−5^
G	5	30	1,000	5.5 × 10^−5^	5.8 × 10^−5^	5.5 × 10^−5^
G	5	30	5,000	4.0 × 10^−5^	4.6 × 10^−5^	4.0 × 10^−5^
G	10	10	1,000	4.6 × 10^−5^	4.0 × 10^−5^	4.6 × 10^−5^
G	10	10	5,000	3.8 × 10^−5^	3.6 × 10^−5^	3.8 × 10^−5^
G	10	30	1,000	5.5 × 10^−5^	5.2 × 10^−5^	5.5 × 10^−5^
G	10	30	5,000	4.9 × 10^−5^	4.5 × 10^−5^	4.9 × 10^−5^
G-GE	2	10	1,000	4.7 × 10^−5^	4.8 × 10^−5^	4.7 × 10^−5^
G-GE	2	10	5,000	4.4 × 10^−5^	4.2 × 10^−5^	4.4 × 10^−5^
G-GE	2	30	1,000	5.7 × 10^−5^	6.0 × 10^−5^	5.6 × 10^−5^
G-GE	2	30	5,000	5.1 × 10^−5^	5.0 × 10^−5^	5.1 × 10^−5^
G-GE	5	10	1,000	4.8 × 10^−5^	4.9 × 10^−5^	4.8 × 10^−5^
G-GE	5	10	5,000	4.9 × 10^−5^	4.3 × 10^−5^	4.9 × 10^−5^
G-GE	5	30	1,000	5.3 × 10^−5^	5.4 × 10^−5^	5.2 × 10^−5^
G-GE	5	30	5,000	5.8 × 10^−5^	5.7 × 10^−5^	5.8 × 10^−5^
G-GE	10	10	1,000	4.6 × 10^−5^	4.1 × 10^−5^	4.6 × 10^−5^
G-GE	10	10	5,000	4.6 × 10^−5^	4.1 × 10^−5^	4.6 × 10^−5^
G-GE	10	30	1,000	5.2 × 10^−5^	5.3 × 10^−5^	5.0 × 10^−5^
G-GE	10	30	5,000	5.8 × 10^−5^	4.9 × 10^−5^	5.8 × 10^−5^

Type I error rates of the conventional score test (CST), the proposed method 1 (PM1), and the proposed method 2 (PM2) at a significance level of *α* = 5 × 10^−5^.

**Fig 2:**
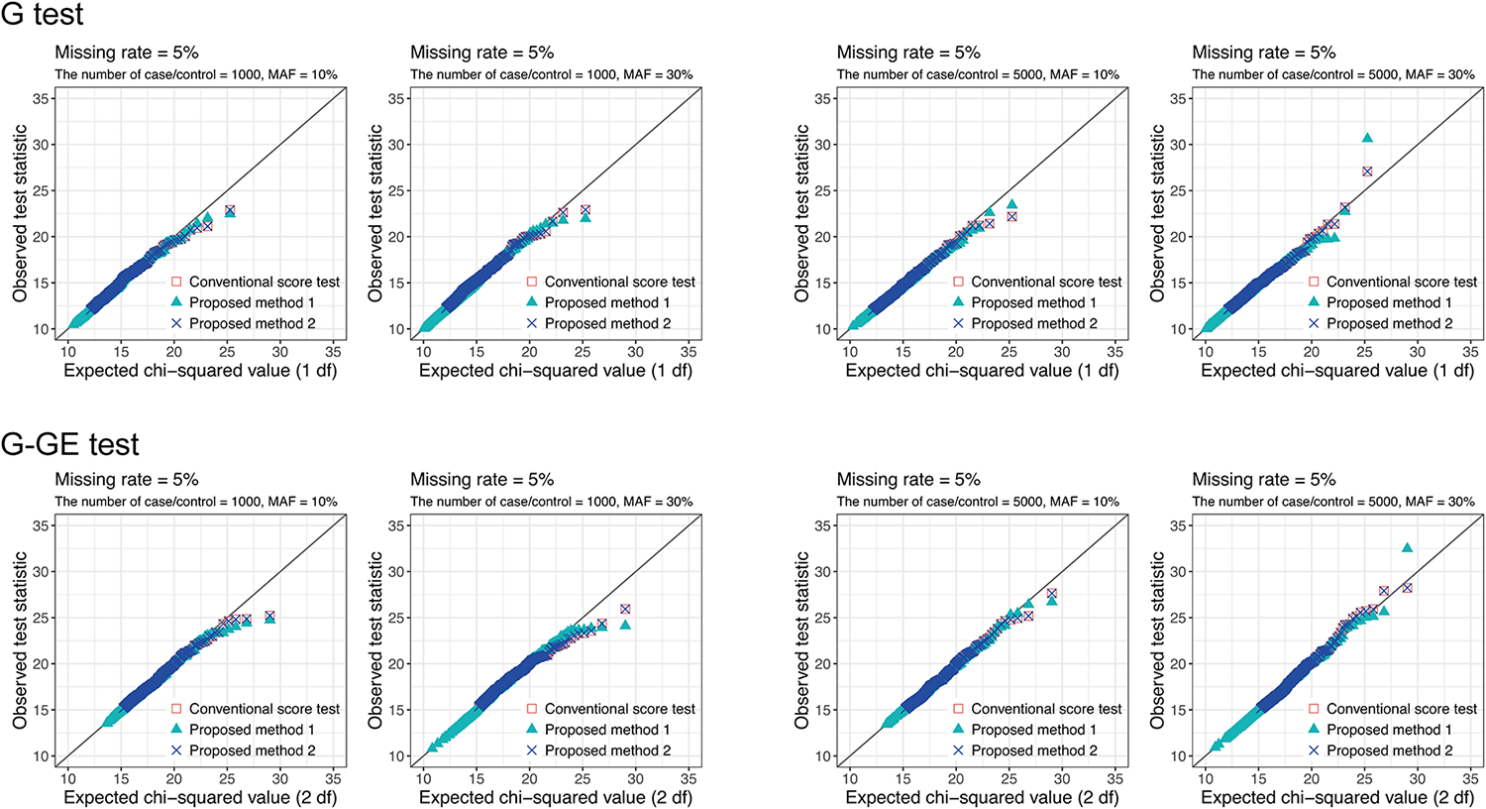
Q-Q plot of the conventional score test and the proposed methods. Chi-squared (1-df or 2-df) Q-Q plot of the top 500 conventional score test, the proposed method 1, and the proposed method 2 score statistics for missing rate is 5% and minor allele frequency (MAF) is 10% and 30% in null simulation.

Next, we constructed quantile-quantile (Q-Q) plots of the distribution of several test settings calculated in the above conditions under the null hypotheses. Fig 2 shows Q-Q plots of G test and G-GE test for missing rate is 5% and MAF is 10% and 30%. We plotted the top 500 score statistics of the CST. Most of the points are distributed around the 45 degree line, which implies that *χ*^2^ approximations to the three score statistics are valid. Q-Q plots in other settings are given in S1 and S2 Figs.

Furthermore, we investigated the scenarios with smaller sample size (the number of case or control: 100, 500) and unbalance sample size (the number of case: 1,000 and control: 2,000) in S1 Table. In the scenarios with smaller sample sizes, especially 100, the type I error rates are slightly lower than the nominal level, but the accuracy is improved as sample sizes get large. On the other hand, the type I error with unbalance scenarios are well-controlled at the nominal level.

#### Power

We performed 1,000 simulation replicates under alternative models to estimate power at *α* = 5 × 10^−8^. We considered a range of missing rate (2%, 5%, 10%, 30%), the number of case or control (100, 500, 1,000, 5,000), unbalance sample size (case/control = 1,000/2,000), and MAF (10%, 30%). Although the missing rate of 30% would be unrealistic in practical human GWAS data, it was set to make the difference in power easy to see for confirmation of our theoretical asymptotic results on power. For reference, we also included the method which simply imputes the missing genotypes by their median (called the median imputation).

**Fig 3:**
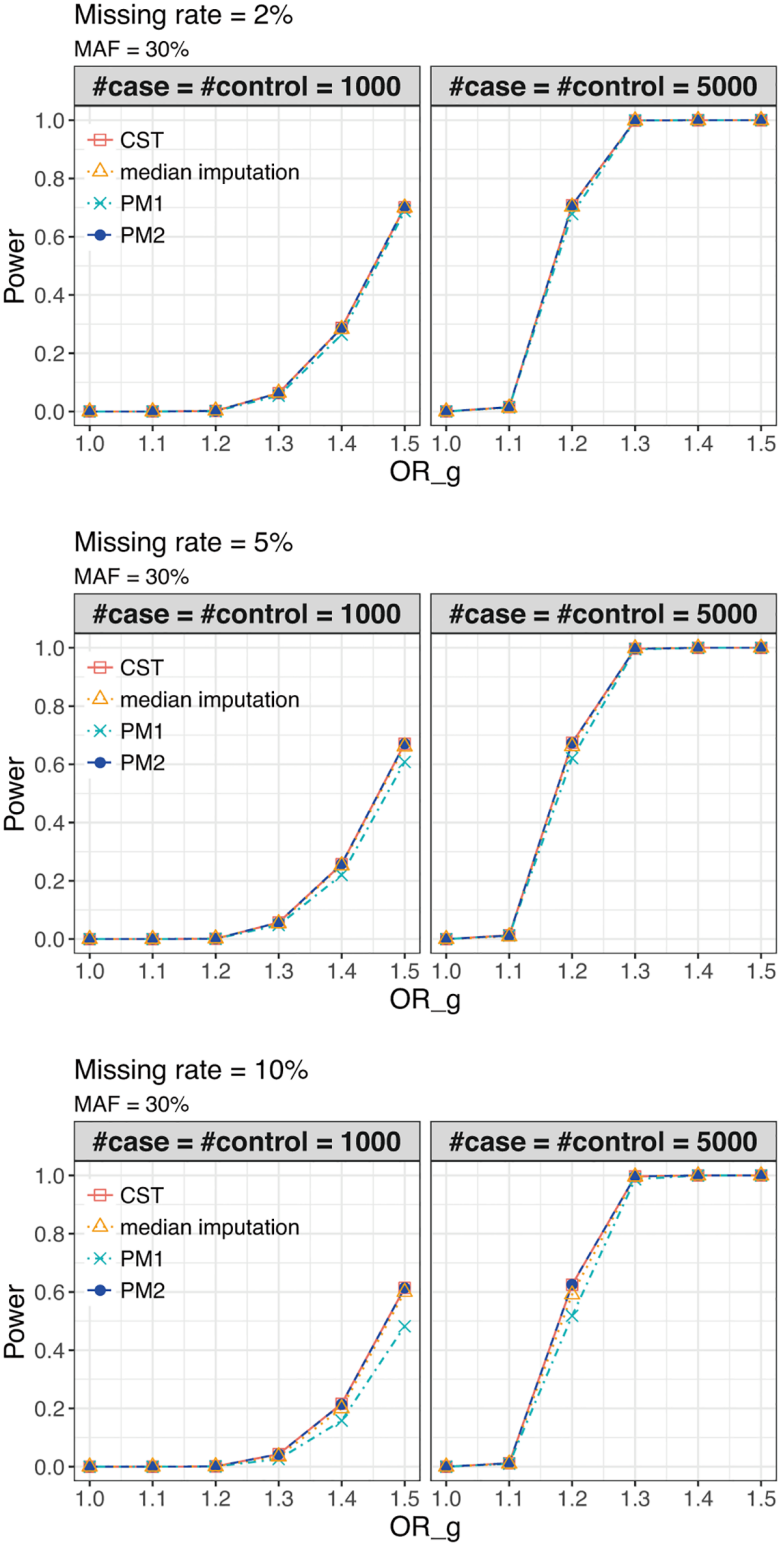
G test Power of the conventional score test and the proposed methods at MAF 30%. G test Power of the conventional score test (CST), the proposed method 1 (PM1), and the proposed method 2 (PM2) under missing rate (2%, 5%, 10%), minor allele frequency (MAF) (30%), and the number of case/control (1,000, 5,000). The x-axis denotes genetic odds ratios (OR_*g*_ = exp(*β*_*g*_)). The significance level is 5 × 10^−8^.

**Fig 4:**
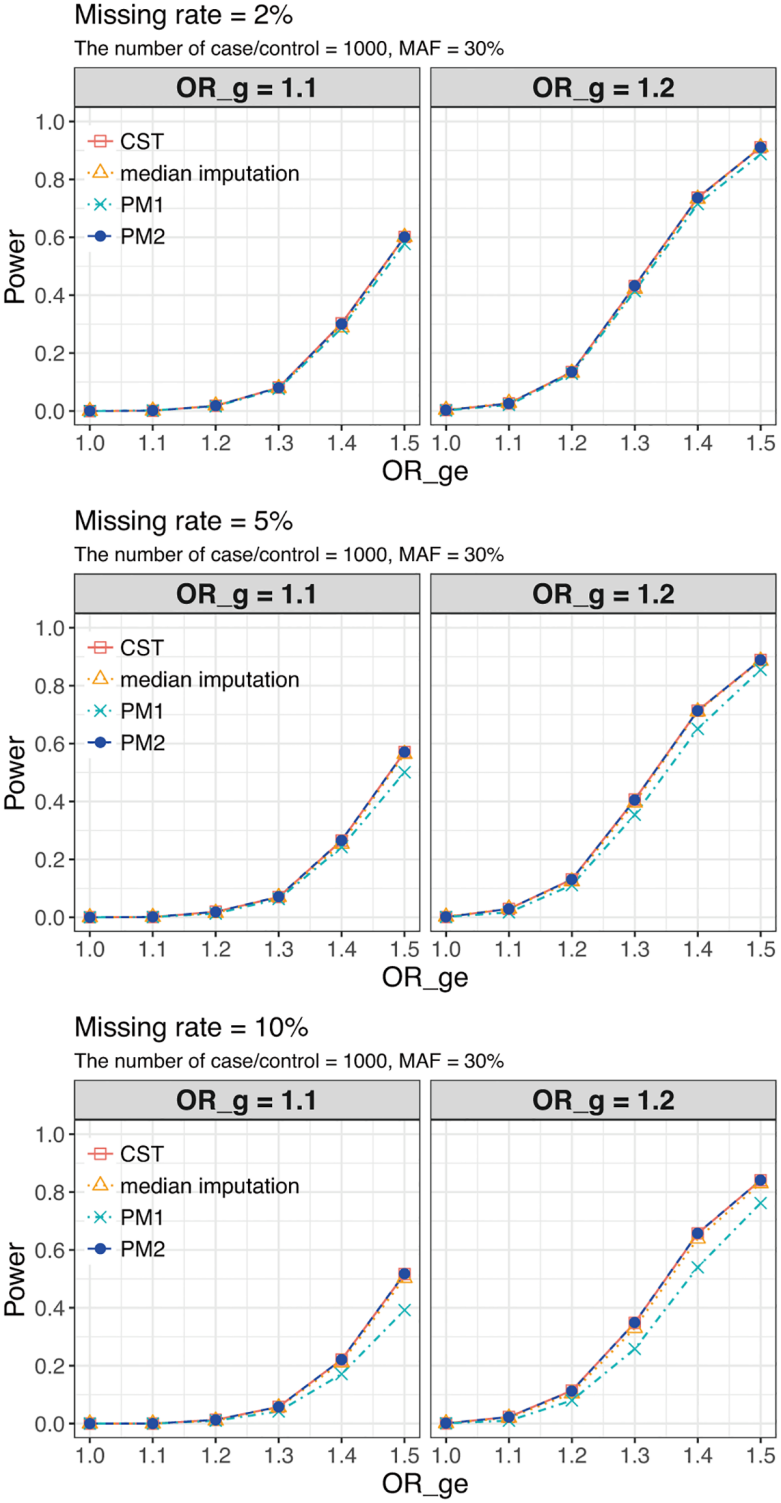
G-GE test Power of the conventional score test and the proposed methods at the number of case/control is 1,000. G-GE test Power of the conventional score test (CST), the proposed method 1 (PM1), and the proposed method 2 (PM2) under genetic odds ratios (OR_*g*_ = exp(*β*_*g*_) = 1.1, 1.2), missing rate (2%, 5%, 10%), minor allele frequency (MAF) (30%), and the number of case/control is 1,000. The x-axis denotes gene-environment interaction odds ratios (OR_*ge*_ = exp(*β*_*ge*_)). The significance level is 5 × 10^−8^.

**Fig 5:**
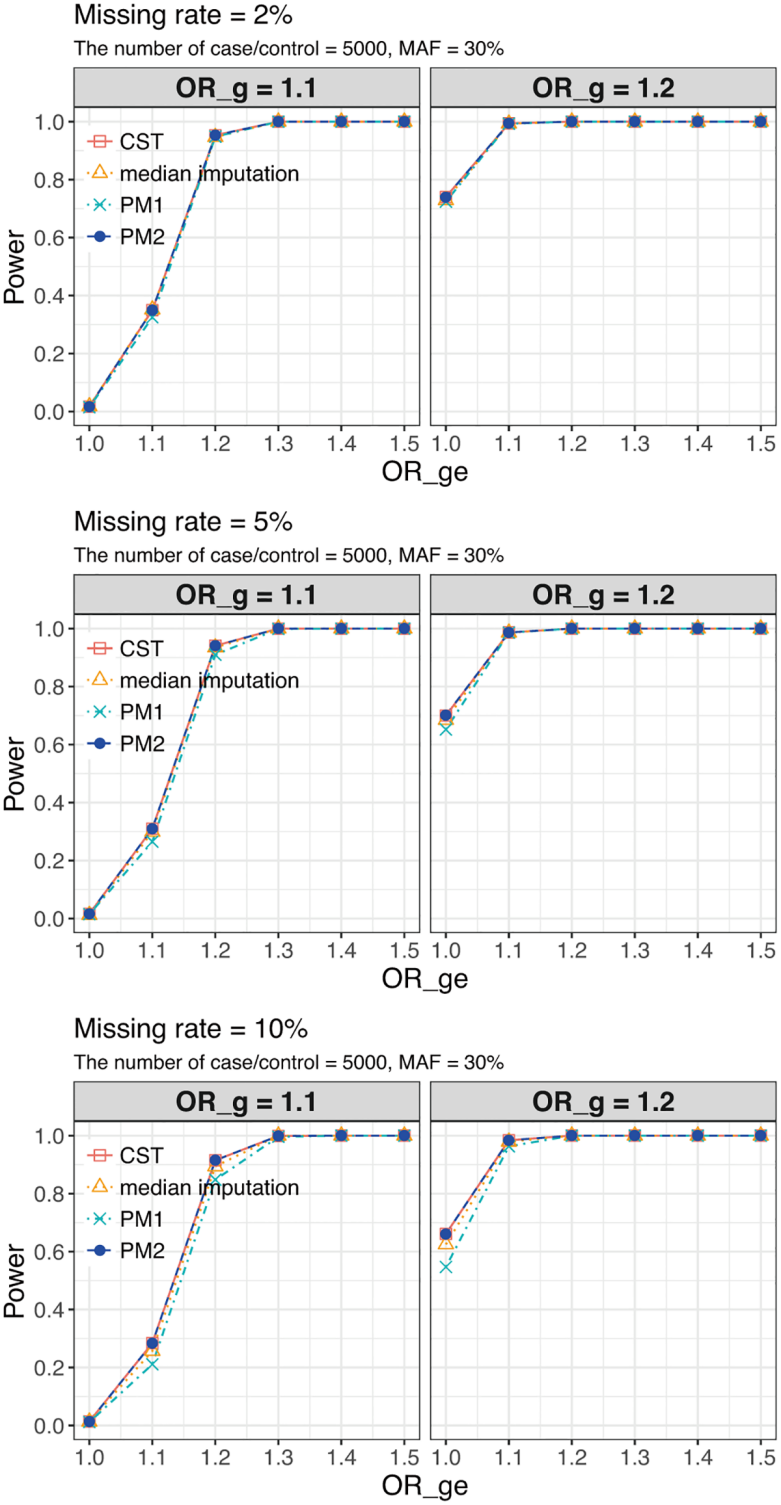
G-GE test Power of the conventional score test and the proposed methods at the number of case/control is 5,000. G-GE test Power of the conventional score test (CST), the proposed method 1 (PM1), and the proposed method 2 (PM2) under genetic odds ratios (OR_*g*_ = exp(*β*_*g*_) = 1.1, 1.2), missing rate (2%, 5%, 10%), minor allele frequency (MAF) (30%), and the number of case/control is 5,000. The x-axis denotes gene-environment interaction odds ratios (OR_*ge*_ = exp(*β*_*ge*_)). The significance level is 5 × 10^−8^.

First, we showed the transition of the power of G tests as the change of OR_*g*_ at MAF of 30% in Fig 3. From Fig 3, we can see that score tests of CST and PM2 are more powerful than PM1. Next, we showed the power transition of the G-GE test as the change of OR_*ge*_ at genetic odds ratios (OR_*g*_ = 1.1, 1.2), missing rate (2%, 5%, 10%), the number of case or control (1,000, 5,000), and MAF (30%) in Figs 4 and 5. Similar to G test, G-GE test also has higher power for CST and PM2 than PM1. In G test and G-GE test, the power of median imputation has slightly lower than CST and PM2, and higher than PM1. Even with a small genetic main effect, the joint test can detect the effect of gene-environment interaction [11]. Analogous results were obtained under other settings (see S3–S5 Figs and S2 and S3 Tables). Collectively, the theoretical results shown in the Materials and Methods section can be confirmed by the simulation studies.

### Application to ADNI GWAS data

We applied our proposed methods to ADNI-GWAS dataset obtained from the publicly available data of the Alzheimer’s Disease Neuroimage Initiative (ADNI) database (adni.loni.usc.edu). The ADNI was launched in 2003 as a public-private partnership, led by Principal Investigator Michael W. Weiner, MD. The primary goal of ADNI has been to test whether serial magnetic resonance imaging (MRI), positron emission tomography (PET), other biological markers, and clinical and neuropsychological assessment can be combined to measure the progression of mild cognitive impairment and early Alzheimer’s disease. For up-to-date information, see www.adni-info.org. ADNI is an ongoing longitudinal study with the primary purpose of exploring the genetic and neuroimaging information associated with late-onset Alzheimer’s disease. The study recruited elderly subjects consisting about 400 subjects with mild cognitive impairment (MCI), about 200 subjects with Alzheimer’s disease (AD), and about 200 healthy controls (normal). Each subject was followed for at least 3 years. During the study period, the subjects were assessed with MRI measures and psychiatric evaluation to determine the diagnosis status at each time point.

The ADNI-GWAS data were obtained from 818 DNA samples of ADNI participants using the Illumina Human 610-Quad genotyping assay [15]. The data initially included 620,901 SNPs. We included the *apolipoprotein* E (APOE) SNP rs429358 on chromosome 19 known to affect AD in our analysis. We used data from 684 non-Hispanic Caucasian samples after we excluded one pair showing cryptic relatedness (revealed by the PLINK pairwise $\hat{\pi }$ statistic being greater than 0.125) [4], and we excluded subjects whose reported sex did not match the sex inferred from X-chromosome SNPs. The total number of remaining SNPs was 528,916, and the demographic variables include gender and age. The distribution of missing rate is shown in the S6 Fig. Of the 528,916 SNPs, 45% of them have missing genotypes. In our work, we used 684 subjects: the status at the baseline of normal, MCI, and AD were 192, 329, and 163, respectively. We defined the following phenotype as an outcome: normal (= 0), MCI (= 0), and AD (= 1) as binary traits. We also included the following covariates in a logistic regression model: gender and age. We compared the proposed methods separately for two subsets of SNPs stratified by missing rate, low missing SNPs (43.9%) with 0% < Missing rate < 1%, and high missing SNPs (11.3%) with Missing rate ≥ 1%.

**Fig 6:**
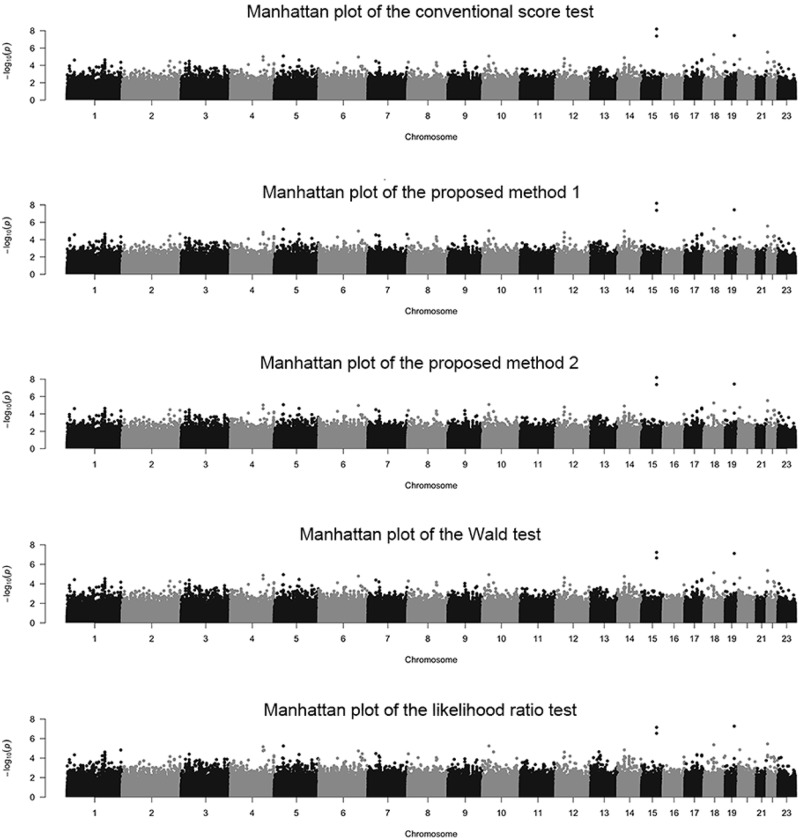
Manhattan plots of each chromosome for ADNI-GWAS dataset with all SNPs. Manhattan plots of each chromosome for ADNI-GWAS dataset. P-values using the conventional score test (CST), the proposed method 1 (PM1) test, the proposed method 2 (PM2) test, the Wald test, and the likelihood ratio test are shown in order from the top. The black and gray points highlight different chromosomes.

Firstly, Fig 6 showed Manhattan plots for all SNPs using CST, PM1, PM2, the Wald test and the likelihood ratio test. All figures were similar in shape, and the APOE SNP was statistically significant in most tests (CST, PM1, and PM2 give P-values of 3.640 × 10^−8^ because of the absence of missing genotypes at the APOE SNP. The Wald and the likelihood ratio tests give P-values of 7.686 × 10^−8^ and 5.372 × 10^−8^, respectively.). Manhattan plots for two SNPs stratified by missing rate are shown in the S7 and S8 Figs and these figures were also similar in shape.

**Fig 7:**
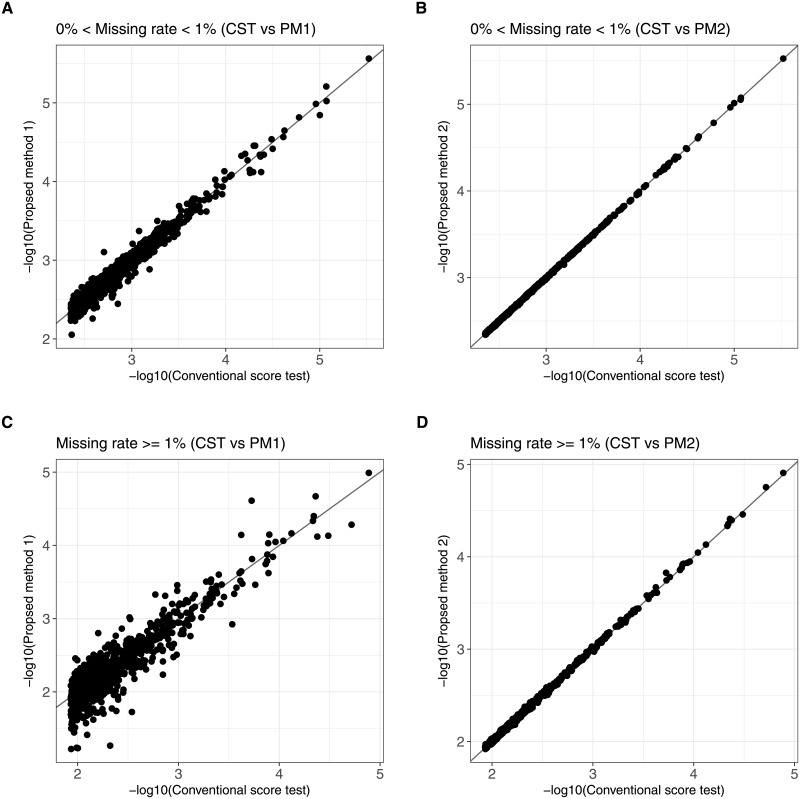
Comparisons of the proposed methods and the conventional score test P-values with the subset SNPs with missing genotypes. Comparisons of the proposed methods (PM1 and PM2) and the conventional score test (CST) P-values that displays only top 1,000. SNPs are stratified by missing rate (low missing SNPs: 0% < Missing rate < 1%, high missing SNPs: Missing rate ≥ 1%).

Secondary, Fig 7 illustrated scatter plots for two SNPs stratified by missing rate comparing top 1,000 P-values of the proposed methods and CST. The Pearson’s correlation coefficient in the low missing SNPs between PM1 and CST was 0.9974 (Fig 7A). On the other hand, the correlation between PM2 and CST was 1.0000 (Fig 7B), showing much higher concordance. Similarly, the Pearson’s correlation coefficient in the high missing SNPs between PM1 and CST was 0.9837 (Fig 7C), and the correlation between PM2 and CST was 1.0000 (Fig 7D). The above results show that the equivalence between test statistics of PM2 and CST and the difference between PM1 and CST (or PM2) as described in the Material and Methods section.

Finally, we compared the run times from the proposed methods, the CST, the Wald test, and the likelihood ratio test on a personal computer (four CPU cores at 4.0 GHz Intel i7) using all SNPs. We implemented all tests in R without using built-in functions (e.g. glm function in R) for fair comparison of execution speed. Table 2 shows the run times. CST showed similar run times to the Wald test as both tests need a single iterative optimization for MLE for each SNP, i.e. MLE under the null model for CST and MLE under the full model for the Wald test. Likelihood ratio test requires two iterative optimizations for MLE under both null and full models, which make run times about twice longer compared with CST or the Wald test. PM1 and PM2 resulted in about 6–18 times faster than the CST, Wald test and likelihood ratio test. A slightly longer run time was observed for PM2 compared to PM1 because PM2 needs more matrix calculation processes than PM1. Based on these findings, we confirmed that the proposed methods have much lower computational cost than the CST, Wald test, and likelihood ratio test.

**Table 2:** Run times (CPU sec) from the proposed methods and the conventional tests.

CST	PM1	PM2	Wald	LRT
452.9	49.2	74.6	460.6	874.5

Run times (CPU sec) from the proposed methods (PM1 and PM2), the conventional score test (CST), the Wald test, and the likelihood ratio test (LRT).

## Discussion

In this paper, we presented two new fast score tests, PM1 and PM2, that require only a single global null estimator for all SNPs for genome-wide scan when missing genotypes are present. We confirmed that our proposed methods can significantly reduce the computational cost compared to conventional tests for genome-wide scans (e.g. Wald test in PLINK) in an application to ADNI-GWAS data. Run time of PM2 is slightly slower than PM1 because PM2 needs more matrix calculation processes. We theoretically proved that PM2 and CST have an equivalent asymptotic power and that the power of PM1 is lower than that of PM2. Additionally, we evaluated the power of CST, PM1, and PM2 by simulation studies and confirmed theoretical results. Therefore, when even higher power is required in studies, PM2 should be used rather than PM1, although PM2 is slightly slower than PM1 in computation. Our approach can speed up the computation by 6–18 times faster than CST, the Wald test, and the likelihood ratio test for genome-wide scans. The CST, Wald, and likelihood ratio tests require re-computation of null MLE for each SNP because the pattern of missing genotypes differs across loci. Our test statistics only use a single global MLE under the null for all SNPs, which avoids re-computing null MLE for each SNP, and the speed-up is independent of the proportion of missing genotypes. The more the number of covariates is, the more computational speed-up is pronounced. Our framework is more valuable for more complicated analyses which require enormous number of hypotheses to be tested such as gene-environment or/and gene-gene interaction analyses.

Missing genotypes may be imputed by the genetic imputation which is a method to predict the genotypes at the SNPs that are not directly assayed in a sample of individuals. It is achieved by using known haplotype reference panel, for example from the HapMap or the 1000 Genomes Project in humans [16, 17]. However, the accuracy of genotype imputation and boosting power of the subsequent association analyses depends on the quality of reference panel. Moreover, genetic imputation requires a lot of computational resources [18–20]. Even if imputation is applied, there usually remain uncertain genotypes that are hard to call, which are often set to missing. Therefore, missing genotype problem is unavoidable even after genetic imputation.

In this study we have assumed MCAR for the proposed methods, which is a reasonable assumption in the case where simply discarding the missing observations (i.e. complete case analysis in the CST) is not too problematic [21]. Although our proposed method worked in real GWAS data from ADNI, there may be a case where missing genotypes cannot be considered as MCAR [22]. Then, simply ignoring missing genotypes from analysis may lead to severe bias [21]. By the same reason, in this case, our theoretical results regarding type I error and power for the proposed methods may not hold. Further work remains to be done in this important topic.

In this paper, we focused only on the logistic regression model for binary traits. However, our framework is general and is extensible to other different score tests, e.g. in survival analysis.

## Supporting information

S1 Appendix Details of the method. More details of the materials and methods section including formulas, derivations, and additional descriptions.
(PDF)

S2 Appendix Program code. A program code of simulations.
(PDF)

S1 Fig Q-Q plot of the conventional score test and the proposed methods at missing rate 2%. Chi-squared (1-df or 2-df) Q-Q plot of the top 500 conventional score test, the proposed method 1, and the proposed method 2 score statistics for missing rate is 2% and minor allele frequency (MAF) is 10% and 30% in null simulation.
(EPS)

S2 Fig Q-Q plot of the conventional score test and the proposed methods at missing rate 10%. Chi-squared (1-df or 2-df) Q-Q plot of the top 500 conventional score test, the proposed method 1, and the proposed method 2 score statistics for missing rate is 10% and minor allele frequency (MAF) is 10% in null simulation.
(EPS)

S3 Fig G test Power of the conventional score test and the proposed methods at MAF 10%. G test Power of the conventional score test (CST), the proposed method 1 (PM1), and the proposed method 2 (PM2) under missing rate (2%, 5%, 10%), minor allele frequency (MAF) (30%), and the number of case/control (1,000, 5,000). The x-axis denotes genetic odds ratios (OR_*g*_ = exp(*β*_*g*_)). The significance level is 5 × 10^−8^.
(EPS)

S4 Fig G-GE test Power of the conventional score test and the proposed methods at the number of case/control is 1,000 and MAF 10%. G-GE test Power of the conventional score test (CST), the proposed method 1 (PM1), and the proposed method 2 (PM2) under genetic odds ratios (OR_*g*_ = exp(*β*_*g*_) = 1.1, 1.2), missing rate (2%, 5%, 10%), minor allele frequency (MAF) (10%), and the number of case/control is 1,000. The x-axis denotes gene-environment interaction odds ratios (OR_*ge*_ = exp(*β*_*ge*_)). The significance level is 5 × 10^−8^.
(EPS)

S5 Fig G-GE test Power of the conventional score test and the proposed methods at the number of case/control is 5,000 and MAF 10%. G-GE test Power of the conventional score test (CST), the proposed method 1 (PM1), and the proposed method 2 (PM2) under genetic odds ratios (OR_*g*_ = exp(*β*_*g*_) = 1.1, 1.2), missing rate (2%, 5%, 10%), minor allele frequency (MAF) (10%), and the number of case/control is 5,000. The x-axis denotes gene-environment interaction odds ratios (OR_*ge*_ = exp(*β*_*ge*_)). The significance level is 5 × 10^−8^.
(EPS)

S6 Fig Missing rate distribution of ADNI. The y-axis denotes the number of SNPs. The x-axis denotes Missing rate.
(EPS)

S7 Fig Manhattan plots of each chromosome for ADNI-GWAS dataset with the low missing SNPs. The y-axis denotes the number of SNPs. The x-axis denotes Missing rate. Low missing population include SNPs with missing (0% < Missing rate < 1%).
(PNG)

S8 Fig Manhattan plots of each chromosome for ADNI-GWAS dataset with the high missing SNPs. The y-axis denotes the number of SNPs. The x-axis denotes Missing rate. High missing population include SNPs with missing (Missing rate ≥ 1%).
(PNG)

S1 Table Type I error rates of the conventional score test and the proposed methods at the scenarios with smaller sample sizes and unbalanced case-control samples. Type I error rates of the conventional score test (CST), the proposed method 1 (PM1), and the proposed method 2 (PM2) at a significance level of *α* = 5 × 10^−5^.
(PDF)

S2 Table G test and G-GE test Power. G test Power of the conventional score test (CST), the proposed method 1 (PM1), and the proposed method 2 (PM2) under missing rate (2%, 5%, 10%, 30%), minor allele frequency (MAF) (10%, 30%), and the number of case/control (1,000, 5,000). The x-axis denotes genetic odds ratios (OR_*g*_ = exp(*β*_*g*_)). The significance level is 5 × 10^−8^. G-GE test Power of CST, PM1, and PM2 under genetic odds ratios (OR_*g*_ = exp(*β*_*g*_) = 1.1, 1.2), missing rate (2%, 5%, 10%, 30%), minor allele frequency (MAF) (10%, 30%), and the number of case/control is 1,000. The x-axis denotes gene-environment interaction odds ratios (OR_*ge*_ = exp(*β*_*ge*_)). The significance level is 5 × 10^−8^.
(PDF)

S3 Table G test and G-GE test Power at the scenarios with smaller sample sizes and unbalance case-control samples. G test Power of the conventional score test (CST), the proposed method 1 (PM1), and the proposed method 2 (PM2) under missing rate (2%, 5%, 10%, 30%), minor allele frequency (MAF) (10%, 30%), and the number of case/control (1,000, 5,000). The x-axis denotes genetic odds ratios (OR_*g*_ = exp(*β*_*g*_)). The significance level is 5 × 10^−8^. G-GE test Power of CST, PM1, and PM2 under genetic odds ratios (OR_*g*_ = exp(*β*_*g*_) = 1.1, 1.2), missing rate (2%, 5%, 10%, 30%), minor allele frequency (MAF) (10%, 30%), and the number of case/control is 1,000. The x-axis denotes gene-environment interaction odds ratios (OR_*ge*_ = exp(*β*_*ge*_)). The significance level is 5 × 10^−8^.
(PDF)
