# Supplementary material for: Fast score test with global null estimation regardless of missing genotypes
Source: PLoS One. 2018 Jul 5;13(7):e0199692. doi: 10.1371/journal.pone.0199692 (PMC6033421; doi:10.1371/journal.pone.0199692)
Supplement: S1 Appendix — More details of the materials and methods section including formulas, derivations, and additional descriptions. (PDF) [file pone.0199692.s001.pdf]

Fast score test with global null estimation regardless of missing genotypes

Shuntaro Sato* Masao Ueki†

Details of the method

Hjort and Claeskens framework

We use the asymptotic results of Hjort and Claeskens to study our statistical methods [1]. Hjort and Claeskens derived an asymptotic distribution of the maximum likelihood estimator (MLE) of the parameters θ under the following distribution for ith subject ($i = 1, \dots, n$),

$$f_i(Y_i) = f_i(Y_i, \theta) = f_i(Y_i, \theta_0 + \delta/\sqrt{n}), \tag{1}$$

in which our θ_0 corresponds to γ_0 in equation (2.2) of Hjort and Claeskens and we omit their θ_0. The parameter δ represents degrees of departures from the null model $f_i(Y_i, \theta_0)$. Thus, $\delta/\sqrt{n}$ represents a small perturbation from the null model, i.e. local alternative. It is suitable to study asymptotic distribution of test statistics.

*shuntarosato@nagasaki-u.ac.jp, Clinical Research Center, Nagasaki University Hospital, 1-7-1 Sakamoto, Nagasaki, Nagasaki 852-8501, Japan. Biostatistics, Graduate School of Medicine, Kurume University, 67 Asahi-machi, Kurume, Fukuoka, 830-0011, Japan.

†Statistical Genetics Team, RIKEN Center for Advanced Intelligence Project, 1-4-1 Nihonbashi, Chuo-ku, Tokyo, 103-0027, Japan.

Under the assumptions in Hjort and Claeskens,

$$f_i(Y_i, \theta_0 + \frac{\delta}{\sqrt{n}}) = f_{0i}(Y_i)\left\{1 + u_i(Y_i)^{\mathrm{T}}\frac{\delta}{\sqrt{n}} + o(\frac{1}{\sqrt{n}})\right\},$$

where $f_{0i}(Y_i) = f_i(Y_i, \theta_0)$ and $u_i(Y_i) = \partial \log f_i(Y_i, \theta_0)/\partial\theta$. Then, the mean of $u_i(Y_i)$ is given by

$$\begin{aligned}
\mathrm{E}\{u_i(Y_i)\} &= \int u_i(Y_i) f_i(Y_i, \theta_0 + \frac{\delta}{\sqrt{n}})dY_i \\
&= \int u_i(Y_i) f_{0i}(Y_i)\left\{1 + u_i(Y_i)^{\mathrm{T}}\frac{\delta}{\sqrt{n}} + o(\frac{1}{\sqrt{n}})\right\} dY_i \\
&= \int u_i(Y_i) f_{0i}(Y) dY_i + \int u_i(Y_i) u_i(Y_i)^{\mathrm{T}} f_{0i}(Y_i) dY_i \frac{\delta}{\sqrt{n}} + o(\frac{1}{\sqrt{n}}) \\
&= \frac{1}{\sqrt{n}} J_{11,i}\delta + o(\frac{1}{\sqrt{n}}), \qquad (2)
\end{aligned}$$

where

$$\begin{aligned}
J_{11,i} &= -\int \frac{\partial^2 \log f_i(Y_i, \theta_0)}{\partial\theta\partial\theta^T} f_{0i}(Y_i) dY_i \\
&= \int u_i(Y_i) u_i(Y_i)^T f_{0i}(Y_i) dY_i
\end{aligned}$$

Similarly, the variance of $u_i(Y_i)$ is

$$\begin{aligned}
\mathrm{var}\{u_i(Y_i)\} &= \int u_i(Y_i) u_i(Y)^{\mathrm{T}} f_i(Y_i, \theta_0 + \frac{\delta}{\sqrt{n}}) dY_i - \mathrm{E}\{u_i(Y_i)\}\mathrm{E}\{u_i(Y_i)\}^{\mathrm{T}} \\
&= \int u_i(Y_i) u_i(Y_i)^{\mathrm{T}} f_{0i}(Y_i)\left\{1 + u_i(Y_i)^{\mathrm{T}}\frac{\delta}{\sqrt{n}} + o(\frac{1}{\sqrt{n}})\right\} dY_i + o(1) \\
&= \int u_i(Y_i) u_i(Y_i)^{\mathrm{T}} f_{0i}(Y_i) dY_i + \int u_i(Y_i) u_i(Y_i)^{\mathrm{T}} u_i(Y_i) f_{0i}(Y_i) dY_i \frac{\delta}{\sqrt{n}} + o(1) \\
&= J_{11,i} + \int u_i(Y_i) u_i(Y_i)^{\mathrm{T}} u_i(Y_i) f_{0i}(Y_i) dY_i \frac{\delta}{\sqrt{n}} + o(1) \\
&= J_{11,i} + o(1). \qquad (3)
\end{aligned}$$

Let $f(Y, \theta_0) = \prod_{i=1}^{n} f_i(Y_i, \theta_0)$. Suppose that θ is partitioned into two parts,

$(\theta_1^{\mathrm{T}}, \theta_2^{\mathrm{T}})^{\mathrm{T}}$, in which θ_1 and θ_2 are s-dimensional and r-dimensional vectors, respectively. For example, a logistic regression model for a SNP is written as

$$\mathrm{logit}[Pr(Y_i = 1)] = \mathrm{logit}[\pi_i(\beta_0, \beta_e, \beta_g)] = \beta_0 + \beta_e E_i + \beta_g G_i \tag{4}$$

where π_i is a probability of being case, G_i is some genotype coding, such as an addition coding $\{0, 1, 2\}$ and E_i is a covariate or an environment factor. Letting $\theta_1 = (\beta_0, \beta_e)^{\mathrm{T}}$, $\theta_2 = \beta_g$, $X_1 = (1, E)$, and $X_2 = G$, the probability of being case at a full model is

$$\pi_i(\theta) = \pi_i(\theta_1, \theta_2) = \frac{\exp(\beta_0 + \beta_e E_i + \beta_g G_i)}{1 + \exp(\beta_0 + \beta_e E_i + \beta_g G_i)} = \frac{\exp(X_1\theta_1 + X_2\theta_2)}{1 + \exp(X_1\theta_1 + X_2\theta_2)}.$$

Under these setting, the log-likelihood function at the full model is

$$\begin{aligned} f(\theta) &= \prod_{i=1}^{n} \{\pi_i^{Y_i}(1 - \pi_i)^{(1-Y_i)}\} \\ \log f(\theta) &= \sum_{i=1}^{n} [Y_i \log \pi_i(\theta) + (1 - Y_i) \log\{1 - \pi_i(\theta)\}]. \end{aligned}$$

In the same way, a logistic regression model for a joint test is written as $\mathrm{logit}[Pr(Y_i = 1)] = \beta_0 + \beta_e E_i + \beta_g G_i + \beta_{ge} G_i E_i$ where $G_i E_i$ is a gene-environment interaction. Letting $\theta_1 = (\beta_0, \beta_e)^{\mathrm{T}}$, $\theta_2 = (\beta_g, \beta_{ge})^{\mathrm{T}}$, $X_1 = (1, E)$, and $X_2 = (G, GE)$. We consider the distribution of $u(Y)$ in the complete data where

$$u(Y) = \begin{pmatrix} u_1(Y) \\ u_2(Y) \end{pmatrix} = \begin{pmatrix} \partial \log f(Y, \theta_0)/\partial\theta_1 \\ \partial \log f(Y, \theta_0)/\partial\theta_2 \end{pmatrix} = \begin{pmatrix} \sum_{i=1}^{n} u_{1i}(Y_i) \\ \sum_{i=1}^{n} u_{2i}(Y_i) \end{pmatrix}.$$

From (2), the mean of $u(Y)$ is

$$\mathrm{E}\begin{pmatrix}\sum_{i=1}^n u_{1i}(Y_i)\\ \sum_{i=1}^n u_{2i}(Y_i)\end{pmatrix} = \frac{1}{\sqrt{n}}\begin{pmatrix}\sum_{i=1}^n J_{11,i}[1,]\delta\\ \sum_{i=1}^n J_{11,i}[2,]\delta\end{pmatrix} + o(\sqrt{n}).$$

From (3), the variance of $u(Y)$ is

$$\begin{aligned}
\mathrm{var}\begin{pmatrix}\sum_{i=1}^n u_{1i}(Y_i)\\ \sum_{i=1}^n u_{2i}(Y_i)\end{pmatrix} &= \sum_{i=1}^n \mathrm{var}\begin{pmatrix}u_{1i}(Y_i)\\ u_{2i}(Y_i)\end{pmatrix} + o(n)\\
&= \sum_{i=1}^n \begin{pmatrix}\mathrm{var}\{u_{1i}(Y_i)\} & \mathrm{cov}\{u_{1i}(Y_i), u_{2i}(Y_i)\}\\ \mathrm{cov}\{u_{2i}(Y_i), u_{1i}(Y_i)\} & \mathrm{var}\{u_{2i}(Y_i)\}\end{pmatrix} + o(n)\\
&= \begin{pmatrix}\sum_{i=1}^n J_{11,i}[1,1] & \sum_{i=1}^n J_{11,i}[1,2]\\ \sum_{i=1}^n J_{11,i}[2,1] & \sum_{i=1}^n J_{11,i}[2,2]\end{pmatrix} + o(n).
\end{aligned}$$

By letting

$$J_{11} = \begin{pmatrix}J_{11}[1,1] & J_{11}[1,2]\\ J_{11}[2,1] & J_{11}[2,2]\end{pmatrix} = \begin{pmatrix}\sum_{i=1}^n J_{11,i}[1,1] & \sum_{i=1}^n J_{11,i}[1,2]\\ \sum_{i=1}^n J_{11,i}[2,1] & \sum_{i=1}^n J_{11,i}[2,2]\end{pmatrix},$$

the distribution of $u(Y)/\sqrt{n}$ is asymptotically

$$\frac{1}{\sqrt{n}}u(Y) \sim \mathrm{N}_{s+r}\left(\frac{1}{n}\begin{pmatrix}J_{11}[1,]\delta\\ J_{11}[2,]\delta\end{pmatrix}, \frac{1}{n}\begin{pmatrix}J_{11}[1,1] & J_{11}[1,2]\\ J_{11}[2,1] & J_{11}[2,2]\end{pmatrix}\right) = \mathrm{N}_{s+r}\left(\frac{1}{n}J_{11}\delta, \frac{1}{n}J_{11}\right), \tag{5}$$

which is exactly the statement in Lemma 3.1 of Hjort and Claeskens.

Similarly, we consider the distribution of $u^m(Y)$ in the presence of missing

data, in which

$$u^m(Y) = \begin{pmatrix} \sum_{i=1}^n u_{1i}(Y_i)I_i \\ \sum_{i=1}^n u_{2i}(Y_i)I_i \end{pmatrix}.$$

The mean of $u^m(Y)$ is

$$\mathrm{E}\begin{pmatrix} \sum_{i=1}^n u_{1i}(Y_i)I_i \\ \sum_{i=1}^n u_{2i}(Y_i)I_i \end{pmatrix} = \frac{1}{\sqrt{n}}\begin{pmatrix} \sum_{i=1}^n J_{11,i}[1,]\delta I_i \\ \sum_{i=1}^n J_{11,i}[2,]\delta I_i \end{pmatrix} + o(\sqrt{n}).$$

The variance of $u^m(Y)$ is

$$\begin{aligned} \mathrm{var}\begin{pmatrix} \sum_{i=1}^n u_{1i}(Y_i)I_i \\ \sum_{i=1}^n u_{2i}(Y_i)I_i \end{pmatrix} &= \sum_{i=1}^n \mathrm{var}\begin{pmatrix} u_{1i}(Y_i)I_i \\ u_{2i}(Y_i)I_i \end{pmatrix} + o(n) \\ &= \sum_{i=1}^n \begin{pmatrix} \mathrm{var}\{u_{1i}(Y_i)I_i\} & \mathrm{cov}\{u_{1i}(Y_i), u_{2i}(Y_i)I_i\} \\ \mathrm{cov}\{u_{2i}(Y_i)I_i, u_{1i}(Y_i)\} & \mathrm{var}\{u_{2i}(Y_i)I_i\} \end{pmatrix} + o(n) \\ &= \begin{pmatrix} \sum_{i=1}^n J_{11,i}[1,1]I_i & \sum_{i=1}^n J_{11,i}[1,2]I_i \\ \sum_{i=1}^n J_{11,i}[2,1]I_i & \sum_{i=1}^n J_{11,i}[2,2]I_i \end{pmatrix} + o(n). \end{aligned}$$

By letting

$$J_{11}^m = \begin{pmatrix} J_{11}^m[1,1] & J_{11}^m[1,2] \\ J_{11}^m[2,1] & J_{11}^m[2,2] \end{pmatrix} = \begin{pmatrix} \sum_{i=1}^n J_{11,i}[1,1]I_i & \sum_{i=1}^n J_{11,i}[1,2]I_i \\ \sum_{i=1}^n J_{11,i}[2,1]I_i & \sum_{i=1}^n J_{11,i}[2,2]I_i \end{pmatrix},$$

Hence, the distribution of $u^m(Y)/\sqrt{n}$ is asymptotically

$$\frac{1}{\sqrt{n}}u^m(Y) \sim \mathrm{N}_{s+r}\left(\frac{1}{n}\begin{pmatrix} J_{11}^m[1,]\delta \\ J_{11}^m[2,]\delta \end{pmatrix}, \frac{1}{n}\begin{pmatrix} J_{11}^m[1,1] & J_{11}^m[1,2] \\ J_{11}^m[2,1] & J_{11}^m[2,2] \end{pmatrix}\right) = \mathrm{N}_{s+r}\left(\frac{1}{n}J_{11}^m\delta, \frac{1}{n}J_{11}^m\right). \tag{6}$$

Analogously, we consider the distribution of $u^{m\prime}(Y)$ in the presence of missing data, where $u^{m\prime}(Y)$ is

$$u^{m\prime}(Y) = \begin{pmatrix} \sum_{i=1}^n u_{1i}(Y_i) \\ \sum_{i=1}^n u_{2i}(Y_i) I_i \end{pmatrix}.$$

The mean of $u^{m\prime}(Y)$ is

$$\mathrm{E}\begin{pmatrix} \sum_{i=1}^n u_{1i}(Y_i) \\ \sum_{i=1}^n u_{2i}(Y_i) I_i \end{pmatrix} = \frac{1}{\sqrt{n}} \begin{pmatrix} \sum_{i=1}^n J_{11,i}[1,]\delta \\ \sum_{i=1}^n J_{11,i}[2,]\delta I_i \end{pmatrix} + o(\sqrt{n}).$$

The variance of $u^{m\prime}(Y)$ is

$$\begin{aligned} \mathrm{var}\begin{pmatrix} \sum_{i=1}^n u_{1i}(Y_i) \\ \sum_{i=1}^n u_{2i}(Y_i) I_i \end{pmatrix} &= \sum_{i=1}^n \mathrm{var}\begin{pmatrix} u_{1i}(Y_i) \\ u_{2i}(Y_i) I_i \end{pmatrix} + o(n) \\ &= \sum_{i=1}^n \begin{pmatrix} \mathrm{var}\{u_{1i}(Y_i)\} & \mathrm{cov}\{u_{1i}(Y_i), u_{2i}(Y_i) I_i\} \\ \mathrm{cov}\{u_{2i}(Y_i) I_i, u_{1i}(Y_i)\} & \mathrm{var}\{u_{2i}(Y_i) I_i\} \end{pmatrix} + o(n) \\ &= \begin{pmatrix} \sum_{i=1}^n J_{11,i}[1,1] & \sum_{i=1}^n J_{11,i}[1,2] I_i \\ \sum_{i=1}^n J_{11,i}[2,1] I_i & \sum_{i=1}^n J_{11,i}[2,2] I_i \end{pmatrix} + o(n). \end{aligned}$$

Hence, the distribution of $u^{m\prime}(Y)/\sqrt{n}$ is asymptotically

$$\frac{1}{\sqrt{n}} u^{m\prime}(Y) \sim \mathrm{N}_{s+r}\left(\frac{1}{n} \begin{pmatrix} J_{11}[1,] \\ J_{11}^m[2,]\delta \end{pmatrix} \delta, \frac{1}{n} \begin{pmatrix} J_{11}[1,1] & J_{11}^m[1,2] \\ J_{11}^m[2,1] & J_{11}^m[2,2] \end{pmatrix} \right) = \mathrm{N}_{s+r}\left(\frac{1}{n} J_{11}^{f\prime}\delta, \frac{1}{n} J_{11}^f \right), \tag{7}$$

in which

$$J_{11}^{f} = \begin{pmatrix} J_{11}[1,1] & J_{11}^{m}[1,2] \\ J_{11}^{m}[2,1] & J_{11}^{m}[2,2] \end{pmatrix} \text{ and } J_{11}^{f\prime} = \begin{pmatrix} J_{11}[1,1] & J_{11}[1,2] \\ J_{11}^{m}[2,1] & J_{11}^{m}[2,2] \end{pmatrix}.$$

Conventional Score Test (CST)

Analysis using only individuals whose genotype data is observed is referred to as the complete case analysis. We call the score test in the complete case analysis as the conventional score test (CST). Let

$$\begin{aligned}
\begin{pmatrix} u_1^m(\theta_1) \\ u_2^m(\theta_1) \end{pmatrix} = \begin{pmatrix} u_1^m(\theta) \\ u_2^m(\theta) \end{pmatrix} \Big|_{\theta=(\theta_1^T,0^T)^T} &= \begin{pmatrix} \sum_{i=1}^n u_{1i}(Y_i,\theta)I_i \\ \sum_{i=1}^n u_{2i}(Y_i,\theta)I_i \end{pmatrix} \Big|_{\theta=(\theta_1^T,0^T)^T} \\
&= \begin{pmatrix} \sum_{i=1}^n \partial \log f_i(Y_i,\theta)I_i/\partial\theta_1 \\ \sum_{i=1}^n \partial \log f_i(Y_i,\theta)I_i/\partial\theta_2 \end{pmatrix} \Big|_{\theta=(\theta_1^T,0^T)^T}.
\end{aligned}$$

Suppose that the null model correspond to the parameter $\theta_0 = (\theta_{10}^T, 0^T)^T$, then

$$u^m(Y) = \begin{pmatrix} u_1^m(Y) \\ u_2^m(Y) \end{pmatrix} = \begin{pmatrix} u_1^m(\theta_{10}) \\ u_2^m(\theta_{10}) \end{pmatrix}.$$

At the null model on CST, i.e. $\theta_2 = 0$, the MLE of θ_1 is denoted by $\check{\theta}_1^m$.

By the Taylor expansion, the score function is written as

$$\begin{aligned}
\frac{1}{\sqrt{n}}\sum_{i=1}^n u_{2i}^m(\check{\theta}_1^m) &= \frac{1}{\sqrt{n}}\sum_{i=1}^n \left\{ u_{2i}^m(\theta_{10}) + \frac{\partial u_{2i}^m(\theta_{10})}{\partial\theta_1}(\check{\theta}_1^m - \theta_{10}) \right\} + o_p(1) \\
&= \frac{1}{\sqrt{n}} n \left\{ \frac{1}{n}\sum_{i=1}^n u_{2i}^m(\theta_{10}) + \frac{1}{n}\sum_{i=1}^n \frac{\partial u_{2i}^m(\theta_{10})}{\partial\theta_1}(\check{\theta}_1^m - \theta_{10}) \right\} + o_p(1) \\
&= \frac{1}{\sqrt{n}} u_2^m(\theta_{10}) - \frac{1}{n} J_{11}^m[2,1]\sqrt{n}(\check{\theta}_1^m - \theta_{10}) + o_p(1).
\end{aligned}$$

Using an analogous argument in the proof of Lemma 3.2 of Hjort and Claeskens,

we have

$$\sqrt{n}(\check{\theta}_1^m - \theta_{10}) = J_{11}^m[1,1]^{-1}\sqrt{n}u_1^m(\theta_{10}) + o_p(1).$$

Substituting the above equation,

$$\begin{aligned}
\frac{1}{\sqrt{n}}\sum_{i=1}^{n} u_{2i}^m(\check{\theta}_1^m) &= \frac{1}{\sqrt{n}}u_2^m(\theta_{10}) - \frac{1}{n}J_{11}^m[2,1]\left\{J_{11}^m[1,1]^{-1}\sqrt{n}u_1^m(\theta_{10}) + o_p(1)\right\} + o_p(1) \\
&= \frac{1}{\sqrt{n}}u_2^m(\theta_{10}) - J_{11}^m[2,1]J_{11}^m[1,1]^{-1}\frac{1}{\sqrt{n}}u_1^m(\theta_{10}) + o_p(1) \qquad (8) \\
&= \begin{pmatrix} -J_{11}^m[2,1]J_{11}^m[1,1]^{-1} & \mathrm{I} \end{pmatrix} \frac{1}{\sqrt{n}} \begin{pmatrix} u_1^m(\theta_{10}) \\ u_2^m(\theta_{10}) \end{pmatrix} + o_p(1) \\
&= L^m \frac{1}{\sqrt{n}} u^m(Y) + o_p(1),
\end{aligned}$$

in which

$$L^m = \begin{pmatrix} -J_{11}^m[2,1]J_{11}^m[1,1]^{-1} & \mathrm{I} \end{pmatrix}.$$

Applying equation (6), under an alternative hypothesis (1), the distribution of CST score function is asymptotically

$$\frac{1}{\sqrt{n}}\sum_{i=1}^{n} u_{2i}^m(\check{\theta}_1^m) \sim N_r(\tfrac{1}{n}L^m J_{11}^m \delta, \tfrac{1}{n}L^m J_{11}^m L^{m\mathrm{T}})$$

where

$$\begin{aligned}
L^m J_{11}^m \delta &= \begin{pmatrix} -J_{11}^m[2,1]J_{11}^m[1,1]^{-1} & \mathrm{I} \end{pmatrix} \begin{pmatrix} J_{11}^m[1,1] & J_{11}^m[1,2] \\ J_{11}^m[2,1] & J_{11}^m[2,2] \end{pmatrix} \delta \\
&= \begin{pmatrix} 0 & -J_{11}^m[2,1]J_{11}^m[1,1]^{-1}J_{11}^m[1,2] + J_{11}^m[2,2] \end{pmatrix} \begin{pmatrix} \delta_1 \\ \delta_2 \end{pmatrix} \\
&= (-J_{11}^m[2,1]J_{11}^m[1,1]^{-1}J_{11}^m[1,2] + J_{11}^m[2,2])\delta_2
\end{aligned}$$

and

$$L^m J_{11}^m L^{m\mathrm{T}} = \begin{pmatrix} -J_{11}^m[2,1]J_{11}^m[1,1]^{-1} & \mathrm{I} \end{pmatrix} \begin{pmatrix} J_{11}^m[1,1] & J_{11}^m[1,2] \\ J_{11}^m[2,1] & J_{11}^m[2,2] \end{pmatrix} \begin{pmatrix} -J_{11}^m[1,1]^{-1}J_{11}^m[1,2] \\ \mathrm{I} \end{pmatrix}$$

$$= \begin{pmatrix} 0 & -J_{11}^m[2,1]J_{11}^m[1,1]^{-1}J_{11}^m[1,2] + J_{11}^m[2,2] \end{pmatrix} \begin{pmatrix} -J_{11}^m[1,1]^{-1}J_{11}^m[1,2] \\ \mathrm{I} \end{pmatrix}$$

$$= -J_{11}^m[2,1]J_{11}^m[1,1]^{-1}J_{11}^m[1,2] + J_{11}^m[2,2].$$

Let $u_m = (1/\sqrt{n})\sum_{i=1}^n u_{2i}^m(\check{\theta}_1^m)$, $\mu_m = \frac{1}{n}L^m J_{11}^m \delta$ and $V_m = \frac{1}{n}L^m J_{11}^m L^{m\mathrm{T}}$. Then, $u_m^{\mathrm{T}} V_m^{-1} u_m$ is the score statistic for testing $H_0 : \theta_2 = 0$. Under the alternative hypothesis, its asymptotic distribution is non-central chi-squared with $r$df and non-centrality parameter

$$\lambda_m = \mu_m^{\mathrm{T}} V_m^{-1} \mu_m = \tfrac{1}{n}(L^m J_{11}^m \delta)^{\mathrm{T}} \left(L^m J_{11}^m L^{m\mathrm{T}}\right)^{-1} (L^m J_{11}^m \delta).$$

Proposed Method 1 (PM1)

Our first proposed method is to use MLE of θ_1 using all individuals at the null model for the score function $u_2^m(\theta_1)$ instead of the MLE $\check{\theta}_1^m$ ignoring individuals whose genotype is missing. The MLE is denoted by $\check{\theta}_1^f$. We call this method the proposed method 1 (PM1). Let

$$u_1(\theta_1) = u_1(\theta)\Big|_{\theta=(\theta_1^T, 0^T)^T} = \sum_{i=1}^n u_{1i}(Y_i, \theta)\Big|_{\theta=(\theta_1^T, 0^T)^T} = \sum_{i=1}^n \partial \log f_i(Y_i, \theta)/\partial \theta_2 \Big|_{\theta=(\theta_1^T, 0^T)^T}.$$

As in the analysis for CST, suppose that the null model correspond to the parameter $\theta_0 = (\theta_{10}^T, 0^T)^T$, then

$$u^{m\prime}(Y) = \begin{pmatrix} u_1(Y) \\ u_2^m(Y) \end{pmatrix} = \begin{pmatrix} u_1(\theta_{10}) \\ u_2^m(\theta_{10}) \end{pmatrix}.$$

Using an analogous argument in the proof of Lemma 3.2 of Hjort and Claeskens, we have

$$\sqrt{n}(\check{\theta}_1^f - \theta_{10}) = J_{11}[1,1]^{-1}\sqrt{n}u_1(\theta_{10}) + o_p(1).$$

Substituting the above equation, the score function is written as

$$\begin{aligned}
\frac{1}{\sqrt{n}}\sum_{i=1}^n u_{2i}^m(\check{\theta}_1^f) &= \frac{1}{\sqrt{n}}\sum_{i=1}^n \left\{ u_{2i}^m(\theta_{10}) + \frac{\partial u_{2i}^m(\theta_{10})}{\partial \theta_1}(\check{\theta}_1^f - \theta_{10}) \right\} + o_p(1) \\
&= \frac{1}{\sqrt{n}} n \left\{ \frac{1}{n}\sum_{i=1}^n u_{2i}^m(\theta_{10}) + \frac{1}{n}\sum_{i=1}^n \frac{\partial u_{2i}^m(\theta_{10})}{\partial \theta_1}(\check{\theta}_1^f - \theta_{10}) \right\} + o_p(1) \\
&= \sqrt{n}\left\{ \frac{1}{n}u_2^m(\theta_{10}) - \frac{1}{n}J_{11}^m[2,1](\check{\theta}_1^f - \theta_{10}) \right\} + o_p(1) \\
&= \frac{1}{\sqrt{n}}u_2^m(\theta_{10}) - \frac{1}{n}J_{11}^m[2,1]\sqrt{n}(\check{\theta}_1^f - \theta_{10}) + o_p(1) \\
&= \frac{1}{\sqrt{n}}u_2^m(\theta_{10}) - \frac{1}{n}J_{11}^m[2,1]\left\{ J_{11}[1,1]^{-1}\sqrt{n}u_1(\theta_{10}) + o_p(1) \right\} + o_p(1) \\
&= \frac{1}{\sqrt{n}}u_2^m(\theta_{10}) - J_{11}^m[2,1]J_{11}[1,1]^{-1}\frac{1}{\sqrt{n}}u_1(\theta_{10}) + o_p(1) \\
&= \begin{pmatrix} -J_{11}^m[2,1]J_{11}[1,1]^{-1} & \mathrm{I} \end{pmatrix} \frac{1}{\sqrt{n}} \begin{pmatrix} u_1(\theta_{10}) \\ u_2^m(\theta_{10}) \end{pmatrix} + o_p(1) \\
&= L^f \frac{1}{\sqrt{n}} u^{m\prime}(Y) + o_p(1),
\end{aligned}$$

where

$$L^f = \begin{pmatrix} -J_{11}^m[2,1]J_{11}[1,1]^{-1} & \mathrm{I} \end{pmatrix}.$$

Applying equation (7), under an alternative hypothesis (1), the distribution of the PM1 score function is

$$\frac{1}{\sqrt{n}}\sum_{i=1}^{n} u_{2i}^m(\check{\theta}_1^f) \sim N_r(\tfrac{1}{n}L^f J_{11}^{f\prime}\delta, \tfrac{1}{n}L^f J_{11}^f {L^f}^{\mathrm{T}}),$$

where

$$\begin{aligned}
L^f J_{11}^{f\prime}\delta &= \begin{pmatrix} -J_{11}^m[2,1]J_{11}[1,1]^{-1} & \mathrm{I} \end{pmatrix} \begin{pmatrix} J_{11}[1,1] & J_{11}[1,2] \\ J_{11}^m[2,1] & J_{11}^m[2,2] \end{pmatrix} \delta \\
&= \begin{pmatrix} 0 & -J_{11}^m[2,1]J_{11}[1,1]^{-1}J_{11}[1,2] + J_{11}^m[2,2] \end{pmatrix} \begin{pmatrix} \delta_1 \\ \delta_2 \end{pmatrix} \\
&= (-J_{11}^m[2,1]J_{11}[1,1]^{-1}J_{11}[1,2] + J_{11}^m[2,2])\delta_2.
\end{aligned}$$

and

$$\begin{aligned}
L^f J_{11}^f {L^f}^{\mathrm{T}} &= \begin{pmatrix} -J_{11}^m[2,1]J_{11}[1,1]^{-1} & \mathrm{I} \end{pmatrix} \begin{pmatrix} J_{11}[1,1] & J_{11}^m[1,2] \\ J_{11}^m[2,1] & J_{11}^m[2,2] \end{pmatrix} \begin{pmatrix} -J_{11}[1,1]^{-1}J_{11}^m[1,2] \\ \mathrm{I} \end{pmatrix} \\
&= \begin{pmatrix} 0 & -J_{11}^m[2,1]J_{11}[1,1]^{-1}J_{11}^m[1,2] + J_{11}^m[2,2] \end{pmatrix} \begin{pmatrix} -J_{11}[1,1]^{-1}J_{11}^m[1,2] \\ \mathrm{I} \end{pmatrix} \\
&= -J_{11}^m[2,1]J_{11}[1,1]^{-1}J_{11}^m[1,2] + J_{11}^m[2,2].
\end{aligned}$$

Let $u_f = (1/\sqrt{n})\sum_{i=1}^n u_{2i}^m(\check{\theta}_1^f)$, $\mu_f = \frac{1}{n}L^f J_{11}^{f\prime}\delta$, and $V_f = \frac{1}{n}L^f J_{11}^f {L^f}^{\mathrm{T}}$. Then, $u_f^{\mathrm{T}} V_f^{-1} u_f$ is the score statistics. Its asymptotic distribution is non-central

chi-squared with $r$df and non-centrality parameter

$$\lambda_f = \mu_f^{\mathrm{T}} V_f^{-1} \mu_f = \tfrac{1}{n}(L^f J_{11}^{f\prime}\delta)^{\mathrm{T}} (L^f J_{11}^f L^{f\,\mathrm{T}})^{-1} (L^f J_{11}^{f\prime}\delta).$$

Proposed Method 2 (PM2)

We propose another score test whose power is greater than PM1, as shown in what follows. Exploiting the approximation in (8), we define a modified score function $u_{2i}^*(\theta_1)$ by

$$u_{2i}^*(\theta_1) = u_{2i}^m(\theta_1) - J_{11}^m[2,1](\theta_1) J_{11}^m[1,1](\theta_1)^{-1} u_{1i}^m(\theta_1),$$

where $J_{11}^m[2,1](\theta)$ and $J_{11}^m[1,1](\theta)$ are $-\{\partial u_2^m(\theta)/\partial\theta_1\}/n$ and $-\{\partial u_1^m(\theta)/\partial\theta_1\}/n$, respectively.

Then, we propose using $u_2^*(\check{\theta}_1^f)$, which is expanded as

$$\begin{aligned}
\frac{1}{\sqrt{n}} \sum_{i=1}^n u_{2i}^*(\check{\theta}_1^f) &= \frac{1}{\sqrt{n}} \sum_{i=1}^n \left\{ u_{2i}^m(\check{\theta}_1^f) - J_{11}^m[2,1](\check{\theta}_1^f) J_{11}^m[1,1](\check{\theta}_1^f)^{-1} u_{1i}^m(\check{\theta}_1^f) \right\} \\
&= \frac{1}{\sqrt{n}} \sum_{i=1}^n \Bigg[\left\{ u_{2i}^m(\theta_{10}) - J_{11}^m[2,1](\check{\theta}_1^f) J_{11}^m[1,1](\check{\theta}_1^f)^{-1} u_{1i}^m(\theta_{10}) \right\} \\
&\quad + \left\{ \frac{\partial u_{2i}^m(\theta_{10})}{\partial\theta_1} - J_{11}^m[2,1](\check{\theta}_1^f) J_{11}^m[1,1](\check{\theta}_1^f)^{-1} \frac{\partial u_{1i}^m(\theta_{10})}{\partial\theta_1} \right\} (\check{\theta}_1^f - \theta_{10}) \Bigg] + o_p(1) \\
&= \frac{1}{\sqrt{n}} \sum_{i=1}^n \left\{ u_{2i}^m(\theta_{10}) - J_{11}^m[2,1](\check{\theta}_1^f) J_{11}^m[1,1](\check{\theta}_1^f)^{-1} u_{1i}^m(\theta_{10}) \right\} \\
&\quad + \frac{1}{\sqrt{n}} \sum_{i=1}^n \left\{ \frac{\partial u_{2i}^m(\theta_{10})}{\partial\theta_1} - J_{11}^m[2,1](\check{\theta}_1^f) J_{11}^m[1,1](\check{\theta}_1^f)^{-1} \frac{\partial u_{1i}^m(\theta_{10})}{\partial\theta_1} \right\} (\check{\theta}_1^f - \theta_{10}) + o_p(1) \\
&= K_1 + K_2(\check{\theta}_1^f - \theta_{10}) + o_p(1).
\end{aligned}$$

Then,

$$
\begin{aligned}
K_2 &= \frac{1}{\sqrt{n}}\sum_{i=1}^{n}\left\{\frac{\partial u_{2i}^m(\theta_{10})}{\partial\theta_1} - J_{11}^m[2,1](\check{\theta}_1^f)J_{11}^m[1,1](\check{\theta}_1^f)^{-1}\frac{\partial u_{1i}^m(\theta_{10})}{\partial\theta_1}\right\}\\
&= \frac{1}{\sqrt{n}}\Big[-J_{11}^m[2,1](\theta_{10}) - J_{11}^m[2,1](\check{\theta}_1^f)J_{11}^m[1,1](\check{\theta}_1^f)^{-1}\{-J_{11}^m[1,1](\theta_{10})\} + o_p(n)\Big]\\
&= \frac{1}{\sqrt{n}}\Big[-J_{11}^m[2,1](\theta_{10}) + \{J_{11}^m[2,1](\theta_{10}) + o_p(n)\}\{J_{11}^m[1,1](\theta_{10})^{-1} + o_p(1/n)\}J_{11}^m[1,1](\theta_{10}) + o_p(n)\Big]\\
&= \frac{1}{\sqrt{n}}\Big[-J_{11}^m[2,1](\theta_{10}) + \{J_{11}^m[2,1](\theta_{10})J_{11}^m[1,1](\theta_{10})^{-1} + o_p(1)\}J_{11}^m[1,1](\theta_{10}) + o_p(n)\Big]\\
&= \frac{1}{\sqrt{n}}\{-J_{11}^m[2,1](\theta_{10}) + J_{11}^m[2,1](\theta_{10})J_{11}^m[1,1](\theta_{10})^{-1}J_{11}^m[1,1](\theta_{10}) + o_p(n)\}\\
&= \frac{1}{\sqrt{n}}o_p(n)\\
&= o_p(\sqrt{n}).
\end{aligned}
$$

Hence,

$$
\begin{aligned}
\frac{1}{\sqrt{n}}\sum_{i=1}^{n}u_{2i}^*(\check{\theta}_1^f) &= \frac{1}{\sqrt{n}}\sum_{i=1}^{n}\Big\{u_{2i}^m(\theta_{10}) - J_{11}^m[2,1](\check{\theta}_1^f)J_{11}^m[1,1](\check{\theta}_1^f)^{-1}u_{1i}^m(\theta_{10})\Big\} + o_p(\sqrt{n})(\check{\theta}_1^f - \theta_{10}) + o_p(1)\\
&= \frac{1}{\sqrt{n}}\sum_{i=1}^{n}\Big\{u_{2i}^m(\theta_{10}) - J_{11}^m[2,1](\check{\theta}_1^f)J_{11}^m[1,1](\check{\theta}_1^f)^{-1}u_{1i}^m(\theta_{10})\Big\} + o_p(\sqrt{n})O_p(1/\sqrt{n}) + o_p(1)\\
&= \frac{1}{\sqrt{n}}\Big\{u_2^m(\theta_{10}) - J_{11}^m[2,1](\check{\theta}_1^f)J_{11}^m[1,1](\check{\theta}_1^f)^{-1}u_1^m(\theta_{10})\Big\} + o_p(1)\\
&= \frac{1}{\sqrt{n}}u_2^m(\theta_{10}) - J_{11}^m[2,1](\check{\theta}_1^f)J_{11}^m[1,1](\check{\theta}_1^f)^{-1}\frac{1}{\sqrt{n}}u_1^m(\theta_{10}) + o_p(1)\\
&= \frac{1}{\sqrt{n}}u_2^m(\theta_1) - \{J_{11}^m[2,1](\theta_{10}) + o_p(n)\}\left\{J_{11}^m[1,1](\theta_{10})^{-1} + o_p(1/n)\right\}\frac{1}{\sqrt{n}}u_1^m(\theta_1) + o_p(1)\\
&= \frac{1}{\sqrt{n}}u_2^m(\theta_{10}) - \left\{J_{11}^m[2,1](\theta_{10})J_{11}^m[1,1](\theta_{10})^{-1} + o_p(1)\right\}\frac{1}{\sqrt{n}}u_1^m(\theta_{10}) + o_p(1)\\
&= \frac{1}{\sqrt{n}}u_2^m(\theta_{10}) - J_{11}^m[2,1](\theta_{10})J_{11}^m[1,1](\theta_{10})^{-1}\frac{1}{\sqrt{n}}u_1^m(\theta_{10}) - o_p(1)\frac{1}{\sqrt{n}}\sum_{i=1}^{n}u_{1i}^m(\theta_{10}) + o_p(1)\\
&= \frac{1}{\sqrt{n}}u_2^m(\theta_{10}) - J_{11}^m[2,1](\theta_{10})J_{11}^m[1,1](\theta_{10})^{-1}\frac{1}{\sqrt{n}}u_1^m(\theta_{10}) + o_p(1).
\end{aligned}
\tag{9}
$$

Comparing (8) and (9), PM2 is asymptotically equivalent to CST.

Comparison of Power between the Conventional Score Test and the Proposed Method 1

Under the alternative hypothesis, the score function and its distribution of CST are

$$
\begin{aligned}
\frac{1}{\sqrt{n}}\sum_{i=1}^{n} u_{2i}^{m}(\check{\theta}_1^{m}) &\sim N_r(L^m J_{11}^m \delta, L^m J_{11}^m L^{m\mathrm{T}}),\\
L^m J_{11}^m \delta &= (-J_{11}^m[2,1]J_{11}^m[1,1]^{-1}J_{11}^m[1,2] + J_{11}^m[2,2])\delta_2,\\
L^m J_{11}^m L^{m\mathrm{T}} &= -J_{11}^m[2,1]J_{11}^m[1,1]^{-1}J_{11}^m[1,2] + J_{11}^m[2,2],
\end{aligned}
$$

and that of PM1 are

$$
\begin{aligned}
\frac{1}{\sqrt{n}}\sum_{i=1}^{n} u_{2i}^{m}(\check{\theta}_1^{f}) &\sim N_r(L^f {J_{11}^f}' \delta, L^f J_{11}^f L^{f\mathrm{T}}),\\
L^f {J_{11}^f}' \delta &= (-J_{11}^m[2,1]J_{11}[1,1]^{-1}J_{11}[1,2] + J_{11}^m[2,2])\delta_2,\\
L^f J_{11}^f L^{f\mathrm{T}} &= -J_{11}^m[2,1]J_{11}[1,1]^{-1}J_{11}^m[1,2] + J_{11}^m[2,2].
\end{aligned}
$$

First, we compare the mean of CST and that of PM1. Recall that I_i is a binary indicator of whether the genotype G_i is observed or not. We make the following assumptions: (i) I_i independently and identically follows a binomial distribution $I_i \sim \mathrm{Bin}(1, 1-R)$ for $i = 1, \cdots, n$, where R is a probability of random missing. Then expected value of I_i is $\mathrm{E}(I_i) = 1-R$; (ii) I_i is independent of a differential score function $\partial u_i(\theta)/\partial\theta$; (iii) $(1/n)\sum_{i=1}^{n} \partial u_i(\theta)/\partial\theta = O(1)$. We consider the quantity in the mean of CST,

$$
\begin{aligned}
J_{11}^m[1,1] = -\frac{1}{n}\frac{\partial u_1^m(\theta)}{\partial\theta_1} &= -\frac{1}{n}\sum_{i=1}^{n}\frac{\partial u_{1i}^m(\theta)}{\partial\theta_1}\\
&= -\frac{1}{n}\sum_{i=1}^{n}\frac{\partial u_{1i}(\theta)}{\partial\theta_1}I_i.
\end{aligned}
$$

Here, the expected value and variance of $(1/n)\sum_{i=1}^{n} \partial u_{1i}(\theta)I_i/\partial\theta_1$ with respect to I_i are

$$\begin{aligned}
\mathrm{E}\left\{\frac{1}{n}\sum_{i=1}^{n}\frac{\partial u_{1i}(\theta)}{\partial\theta_1}I_i\right\} &= \frac{1}{n}\sum_{i=1}^{n}\frac{\partial u_{1i}(\theta)}{\partial\theta_1}\mathrm{E}(I_i) \\
&= (1-R)\cdot\frac{1}{n}\sum_{i=1}^{n}\frac{\partial u_{1i}(\theta_1)}{\partial\theta_1}
\end{aligned}$$

and

$$\begin{aligned}
\mathrm{var}\left\{\frac{1}{n}\sum_{i=1}^{n}\frac{\partial u_{1i}(\theta)}{\partial\theta_1}I_i\right\} &= \sum_{i=1}^{n}\left\{\frac{1}{n}\frac{\partial u_{1i}(\theta)}{\partial\theta_1}\right\}^2\mathrm{var}(I_i) \\
&= R(1-R)\cdot\frac{1}{n^2}\sum_{i=1}^{n}\left\{\frac{\partial u_{1i}(\theta)}{\partial\theta_1}\right\}^2.
\end{aligned}$$

From the assumption (iii),

$$\mathrm{var}\left\{\frac{1}{n}\sum_{i=1}^{n}\frac{\partial u_{1i}(\theta)}{\partial\theta_1}I_i\right\} = O(1/n).$$

By Markov's inequality, for any $\epsilon > 0$,

$$\begin{aligned}
Pr\left(\left|\frac{1}{n}\sum_{i=1}^{n}\frac{\partial u_{1i}(\theta)}{\partial\theta_1}I_i - \frac{1-R}{n}\sum_{i=1}^{n}\frac{\partial u_{1i}(\theta)}{\partial\theta_1}\right| > \epsilon\right) &= Pr\left(\left|\frac{1}{n}\sum_{i=1}^{n}\frac{\partial u_{1i}(\theta)}{\partial\theta_1}I_i - \frac{1-R}{n}\sum_{i=1}^{n}\frac{\partial u_{1i}(\theta)}{\partial\theta_1}\right|^2 > \epsilon^2\right) \\
&\le \mathrm{E}\left\{\frac{\left|\frac{1}{n}\sum_{i=1}^{n}\frac{\partial u_{1i}(\theta)}{\partial\theta_1}I_i - \frac{1-R}{n}\sum_{i=1}^{n}\frac{\partial u_{1i}(\theta)}{\partial\theta_1}\right|^2}{\epsilon^2}\right\} \\
&= \frac{\mathrm{var}\left\{\frac{1}{n}\sum_{i=1}^{n}\frac{\partial u_{1i}(\theta)}{\partial\theta_1}I_i\right\}}{\epsilon^2} \\
&= O(1/n) \to 0.
\end{aligned}$$

Thus, by the law of large numbers,

$$\begin{aligned} J_{11}^m[1,1] &= -\frac{1}{n}\sum_{i=1}^n \frac{\partial u_{1i}(\theta)}{\partial \theta_1} I_i \\ &\xrightarrow{p} \mathrm{E}\left\{ -\frac{1}{n}\sum_{i=1}^n \frac{\partial u_{1i}(\theta)}{\partial \theta_1} I_i \right\} \\ &= (1-R)\left\{ -\frac{1}{n}\sum_{i=1}^n \frac{\partial u_{1i}(\theta)}{\partial \theta_1} \right\} \\ &= (1-R) J_{11}[1,1]. \end{aligned} \tag{10}$$

Similarly, $J_{11}^m[1,2]$ is

$$J_{11}^m[1,2] \xrightarrow{p} (1-R) J_{11}[1,2]. \tag{11}$$

From (10) and (11), it follows that

$$\begin{aligned} J_{11}^m[1,1]^{-1} J_{11}^m[1,2] &\xrightarrow{p} \frac{1}{1-R} J_{11}[1,1]^{-1}(1-R) J_{11}[1,2] \\ &= J_{11}[1,1]^{-1} J_{11}[1,2]. \end{aligned} \tag{12}$$

Hence, from (12), the mean of CST, $L^m J_{11}^m \delta$, is asymptotically equivalent to that of PM1, $L^f J_{11}^{f\,\prime} \delta$.

Second, we compare the variance of CST and that of PM1. To this end, we may only compare $J_{11}^m[1,1]$ and $J_{11}[1,1]$. The former is

$$J_{11}^m[1,1] = -\frac{1}{n}\sum_{i=1}^n \frac{\partial u_{1i}^m(\theta)}{\partial \theta_1} = -\frac{1}{n}\sum_{i=1}^n \frac{\partial u_{1i}(\theta)}{\partial \theta_1} I_i$$

and the letter is

$$J_{11}[1,1] = -\frac{1}{n}\sum_{i=1}^n \frac{\partial u_{1i}(\theta)}{\partial \theta_1}.$$

Assuming positive-semidefiniteness of $\partial u_{1i}(\theta)/\partial\theta_1$ for all i, we deduce that $J_{11}[1,1] \geq J_{11}^m[1,1]$, in which we denote that, for two matrices A and B, $A \geq B$ if $A - B$ is positive-semidefinite. Thus, $-J_{11}[1,1]^{-1} \geq -J_{11}^m[1,1]^{-1}$. Consequently, the variance of PM1 $L^m J_{11}^m L^{m\mathrm{T}}$, is bigger than that of CST, $L^f J_{11}^f L^{f\mathrm{T}}$.

If a score function u follows the normal distribution with mean μ and variance V, the quadratic form $u^{\mathrm{T}} V^{-1} u$ (test statistic) follows the non-central chi-square distribution, where the non-centrality parameter is $\mu^{\mathrm{T}} V^{-1} \mu$. The power of the test statistic increases as the non-centrality parameter increases. In the above arguments, we have shown that the mean of CST score function is asymptotically equivalent to that of PM1 score function while the variance of PM1 score function is bigger than the variance of CST score function. That is, the magnitude of non-centrality parameter is dominated only by the magnitude of variance, and the non-centrality parameter of PM1 is smaller than that of CST. Therefore, the power of PM1 is smaller than that of CST.

References

[1] Hjort NL, Claeskens G. Frequentist model average estimators. Journal of the American Statistical Association. 2003;98(464):879–899.
