# Supplementary material for: Fast score test with global null estimation regardless of missing genotypes
Source: PLoS One. 2018 Jul 5;13(7):e0199692. doi: 10.1371/journal.pone.0199692 (PMC6033421; doi:10.1371/journal.pone.0199692)
Supplement: S2 Appendix — A program code of simulations. (PDF) [file pone.0199692.s002.pdf]

```
#-----------------------------------------------------------------------
# Date: 2018/4/10
# Author: Shuntaro Sato, Masao Ueki
# R code for "Fast score test with global null estimation regardless of missing genotypes"?.
#-----------------------------------------------------------------------

# Simulation -----------------------------------------------------------
# Before running the following simulation codes, please copy & paste the module code for simulation.
# Check Type I error
# Initial set up (Example)
# R <- 1e+6    # Number of simulation replicates
R <- 1000
seed <- 2017    # Random seed
beta1 <- log(1.2)    # log Odds ratio of genetic effect
beta2 <- log(1)    # log Odds ratio of environmental effect
beta3 <- log(1)    # log Odds ratio of gene-environmental interaction
prevalence <- 0.01    # Prevalence of disease
p.cov <- 0.5    # Ratio of population frequency of covariate
ncase <- 1000    # Number of case
ncontrol <- 1000    # Number of control
maf <- 0.3    # Minor allele frequency
p.missing <- 0.1    # Missing genotype rate
alpha <- 5e-2    # Nominal significance level


# G test
result_alpha_g <- CalcurateScoreStatistics(R, seed, prevalence, beta0, beta1, beta2, ncase, ncontrol, maf, p.cov)
# Type I error rates at the nominal level alpha
CalculateTypeIerror_g(result_alpha_g, alpha, ncase, ncontrol, maf)

#G-GE test
result_alpha_ge <- CalcurateScoreStatistics_ge(R, seed, prevalence, beta0, beta1, beta2, beta3, ncase, ncontrol, maf,
p.cov)
# Type I error rates at the nominal level alpha
CalculateTypeIerror_ge(result_alpha_ge, alpha, ncase, ncontrol, maf)


# Check Power
# Initial set up (Example)
R <- 1000    # Number of simulation replicates
seed <- 2016    # Random seed
beta1 <- log(1.2)    # log Odds ratio of genetic effect
prevalence <- 0.01    # Prevalence of disease
alpha <- 5e-8    # Nominal significance level
p.cov <- 0.5    # Ratio of population frequency of covariate
p.missing <- 0.1    # Missing genotype rate
maf <- 0.3    # Minor allele frequency
ncase <- 1000    # Number of case
ncontrol <- 1000    # Number of control
# G test
# Calcurate the transition of the power of G tests as the change of OR_genetic (1 to 1.5 by 0.1)
(result_power_g <- t(sapply(seq(1, 1.5, 0.1), f.power.g)))
# G-GE test
beta2 <- log(1.1)    # log Odds ratio of environmental effect
# Calcurate the transition of the power of G-GE tests as the change of OR_G-E interaction (1 to 1.5 by 0.1)
(result_power_ge <- t(sapply(seq(1, 1.5, 0.1), f.power.ge)))


# Before running the above codes, please copy & paste the following.
# Module for simulation ------------------------------------------------
# G Test
# We explore the beta0 corresponding to the prevalence.
ExploreBeta0<- function(prevalence, beta1, beta2, maf, p.cov){

  beta0 <- seq(-10, 0 , 0.0001)

  p <- (1-maf)^2*(1-p.cov)/(1+exp(-beta0)) +
    2*maf*(1-maf)*(1-p.cov)/(1+exp(-beta0-beta2)) +
    maf^2*(1-p.cov)/(1+exp(-beta0-2*beta2)) +
    (1-maf)^2*p.cov/(1+exp(-beta0-beta1)) +
    2*maf*(1-maf)*p.cov/(1+exp(-beta0-beta1-beta2)) +
    maf^2*p.cov/(1+exp(-beta0-beta1-2*beta2))

  diffp <- abs(prevalence - p)

  z <- cbind(beta0, p, diffp)
  mini.beta0 <- z[which.min(diffp),][1]
  mini.beta0
}

#We create simulation data.
CreateData <- function(prevalence, beta0, beta1, beta2, ncase, ncontrol, maf, p.cov){

  #set up
  row <- c(1,2,3,4,5,6)
```

```
  #case control sampling----------------------------------------------------------------
  #y=1
  p_y1_a0b0 <- dbinom(0, 2, maf) * dbinom(0, 1, p.cov) * (1 / prevalence) * (1 / (1 + exp(-beta0 - beta1*0 -
beta2*0)))
  p_y1_a1b0 <- dbinom(1, 2, maf) * dbinom(0, 1, p.cov) * (1 / prevalence) * (1 / (1 + exp(-beta0 - beta1*0 -
beta2*1)))
  p_y1_a2b0 <- dbinom(2, 2, maf) * dbinom(0, 1, p.cov) * (1 / prevalence) * (1 / (1 + exp(-beta0 - beta1*0 -
beta2*2)))
  p_y1_a0b1 <- dbinom(0, 2, maf) * dbinom(1, 1, p.cov) * (1 / prevalence) * (1 / (1 + exp(-beta0 - beta1*1 -
beta2*0)))
  p_y1_a1b1 <- dbinom(1, 2, maf) * dbinom(1, 1, p.cov) * (1 / prevalence) * (1 / (1 + exp(-beta0 - beta1*1 -
beta2*1)))
  p_y1_a2b1 <- dbinom(2, 2, maf) * dbinom(1, 1, p.cov) * (1 / prevalence) * (1 / (1 + exp(-beta0 - beta1*1 -
beta2*2)))
  p_y1 <- c(p_y1_a0b0, p_y1_a1b0, p_y1_a2b0, p_y1_a0b1, p_y1_a1b1, p_y1_a2b1)

  x1_y1 <- numeric(ncase)
  x2_y1 <- numeric(ncase)
  u1 <- t(row %*% rmultinom(ncase, 1, p_y1))

  x1_y1[u1==1] <- 0
  x1_y1[u1==2] <- 0
  x1_y1[u1==3] <- 0
  x1_y1[u1==4] <- 1
  x1_y1[u1==5] <- 1
  x1_y1[u1==6] <- 1

  x2_y1[u1==1] <- 0
  x2_y1[u1==2] <- 1
  x2_y1[u1==3] <- 2
  x2_y1[u1==4] <- 0
  x2_y1[u1==5] <- 1
  x2_y1[u1==6] <- 2

  y_y1 <- rep(1, ncase)

  #y=0
  p_y0_a0b0 <- dbinom(0 ,2, maf) * dbinom(0, 1, p.cov) * (1 / (1 - prevalence)) * (exp(-beta0 - beta1*0 - beta2*0) /
(1 + exp(-beta0 - beta1*0 - beta2*0)))
  p_y0_a1b0 <- dbinom(1 ,2, maf) * dbinom(0, 1, p.cov) * (1 / (1 - prevalence)) * (exp(-beta0 - beta1*0 - beta2*1) /
(1 + exp(-beta0 - beta1*0 - beta2*1)))
  p_y0_a2b0 <- dbinom(2 ,2, maf) * dbinom(0, 1, p.cov) * (1 / (1 - prevalence)) * (exp(-beta0 - beta1*0 - beta2*2) /
(1 + exp(-beta0 - beta1*0 - beta2*2)))
  p_y0_a0b1 <- dbinom(0 ,2, maf) * dbinom(1, 1, p.cov) * (1 / (1 - prevalence)) * (exp(-beta0 - beta1*1 - beta2*0) /
(1 + exp(-beta0 - beta1*1 - beta2*0)))
  p_y0_a1b1 <- dbinom(1 ,2, maf) * dbinom(1, 1, p.cov) * (1 / (1 - prevalence)) * (exp(-beta0 - beta1*1 - beta2*1) /
(1 + exp(-beta0 - beta1*1 - beta2*1)))
  p_y0_a2b1 <- dbinom(2 ,2, maf) * dbinom(1, 1, p.cov) * (1 / (1 - prevalence)) * (exp(-beta0 - beta1*1 - beta2*2) /
(1 + exp(-beta0 - beta1*1 - beta2*2)))
  p_y0<- c(p_y0_a0b0, p_y0_a1b0, p_y0_a2b0, p_y0_a0b1, p_y0_a1b1, p_y0_a2b1)

  x1_y0 <- numeric(ncontrol)
  x2_y0 <- numeric(ncontrol)
  u0 <- t(row %*% rmultinom(ncontrol,1,p_y0))

  x1_y0[u0==1] <- 0
  x1_y0[u0==2] <- 0
  x1_y0[u0==3] <- 0
  x1_y0[u0==4] <- 1
  x1_y0[u0==5] <- 1
  x1_y0[u0==6] <- 1

  x2_y0[u0==1] <- 0
  x2_y0[u0==2] <- 1
  x2_y0[u0==3] <- 2
  x2_y0[u0==4] <- 0
  x2_y0[u0==5] <- 1
  x2_y0[u0==6] <- 2

  y_y0 <- rep(0, ncontrol)

  e <- c(x1_y1, x1_y0)  #snp
  g <- c(x2_y1, x2_y0)  #covariate
  y <- c(y_y1, y_y0)
  simulation.data <- cbind(y, e, g)
  simulation.data
}


# Create missing data for SNP(g).
MissingData <- function(sim.data, p.missing, ncase, ncontrol){
  ndata <- ncase + ncontrol
  sim.data[, 3][runif(ndata) < p.missing] <- NA    #g <- missing
  sim.data
```

```
}

# My own GLM
piFunc<- function(Beta, X){
  pi <- 1 / (1 + exp(-X %*% Beta))
}

# Null estimation
f.glm.null <- function(y, e){
  #initial value
  beta.0.i <- 0
  beta.e.i <- 0
  Beta <- rbind(beta.0.i, beta.e.i)

  intercept <- rep(1, length(y))
  X <- cbind(intercept, e)
  i <- 1
  first <- c(Beta, sqrt(sum(sum(t(X) %*% (y - piFunc(Beta, X))) ^ 2)))
  repeat
  {
    i <- i + 1
    pi <- piFunc(Beta, X)
    #score function
    U <- t(X) %*% (y - pi)
    #Information matrix
    w <- as.numeric(pi * (1 - pi))
    I <- crossprod(X, w*X)
    iI <- solve(I)

    #Update
    Beta <- Beta + iI %*% U
    pi <- piFunc(Beta, X)
    U <- t(X) %*% (y - pi)
    w <- as.numeric(pi * (1 - pi))
    I <- crossprod(X, w*X)
    iI <- solve(I)

    SE <- sqrt(diag(iI))
    cof.SE <- cbind(Beta, SE)

    #Stop criteria
    U <- t(X) %*% (y - pi)
    nU <- sqrt(sum(U^2))
    first <- rbind(first,c(Beta, nU))
    if(i == 100)break
    if(sqrt(sum((first[i,1:2] - first[i-1,1:2])^2)) <= 1e-7)break
    if(nU <= 1e-6)break
  }
  cof.SE
}

# Calculate Score statistics
CalcurateScoreStatistics <- function(R, seed, prevalence, beta0, beta1, beta2, ncase, ncontrol, maf, p.cov){

  score.cv <- numeric(R)
  score.pm1 <- numeric(R)
  score.pm2 <- numeric(R)
  score.med <- numeric(R)

  set.seed(seed)

  for (r in 1:R) {
    beta0 <- as.numeric(ExploreBeta0(prevalence, beta1, beta2, maf, p.cov))
    sim.data <- CreateData(prevalence, beta0, beta1, beta2, ncase, ncontrol, maf, p.cov)
    missing.sim.data <- MissingData(sim.data, p.missing, ncase, ncontrol)

    #  Conventional score test
    complete.data <-as.matrix(missing.sim.data[complete.cases(missing.sim.data), ])    #missing omit

    y.com <- complete.data[, 1]
    intercept.com <- rep(1, length(y.com))
    e.com <- complete.data[, 2]
    g.com <- complete.data[, 3]
    X.com <- cbind(intercept.com, e.com, g.com)
    X2.com <- cbind(g.com)

    glm.com <- f.glm.null(y.com, e.com)
    pi.com <- piFunc(glm.com[,1], X.com[,1:2])
    w.com <- as.numeric(pi.com * (1 - pi.com))
    I.com <- crossprod(X.com, w.com*X.com)
    iI.com <- solve(I.com)
    U.com <- t(X2.com) %*% (y.com - pi.com)
    score.cv[r] <- t(U.com) %*% iI.com[3, 3] %*% U.com
```

```
    # Proposed method 1
    y.mis1 <- missing.sim.data[, 1]
    intercept.mis1 <- rep(1, length(y.mis1))
    e.mis1 <- missing.sim.data[, 2]
    g.mis1 <- missing.sim.data[, 3]
    g.mis1[is.na(g.mis1) == TRUE] <- 0    #if missing then 0
    X.mis1 <- cbind(intercept.mis1, e.mis1, g.mis1)
    X2.mis1 <- cbind(g.mis1)

    glm.mis1 <- f.glm.null(y.mis1, e.mis1)    #null model
    pi.mis1 <- piFunc(glm.mis1[,1], X.mis1[,1:2])
    w.mis1 <- as.numeric(pi.mis1 * (1 - pi.mis1))
    I.mis1 <- crossprod(X.mis1, w.mis1*X.mis1)
    iI.mis1 <- solve(I.mis1)
    U.mis1 <- t(X2.mis1) %*% (y.mis1 - pi.mis1)
    score.pm1[r] <- t(U.mis1) %*% iI.mis1[3, 3] %*% U.mis1


    # Proposed method 2
    inmis <- which(!is.na(missing.sim.data[, 3]))
    X.mis2 <- cbind(intercept.mis1, e.mis1, g.mis1)[inmis,]
    U.mis2 <-  t(X.mis2) %*% (y.mis1[inmis] - pi.mis1[inmis])    #(4, 1)
    I.mis2 <- crossprod(X.mis2, w.mis1[inmis]*X.mis2)    #(4, 4)

    iI.mis2.11 <- solve(I.mis2[1:2, 1:2])    #(3, 3)
    U.mis2.ast <- U.mis2[3] - I.mis2[3,1:2] %*% iI.mis2.11 %*% U.mis2[1:2]    #(1, 1)

    iImod.mis2 <- solve(-I.mis2[3,1:2] %*% iI.mis2.11 %*% I.mis2[1:2,3] + I.mis2[3,3])

    score.pm2[r] <- t(U.mis2.ast) %*% iImod.mis2 %*% U.mis2.ast


    # Median imputation
    g.med <- missing.sim.data[, 3]
    median.g <- median(g.med, na.rm = TRUE)
    g.med[is.na(g.med) == TRUE] <- median.g    #if missing then 0
    X.med <- cbind(intercept.mis1, e.mis1, g.med)
    X2.med <- cbind(g.med)
    I.med <- crossprod(X.med, w.mis1*X.med)
    iI.med <- solve(I.med)
    U.med <- t(X2.med) %*% (y.mis1 - pi.mis1)
    score.med[r] <- t(U.med) %*% iI.med[3, 3] %*% U.med
  }
  result.score <- cbind(score.cv, score.pm1, score.pm2, score.med)
  result.score
}


# Calculate Type I error.
CalculateTypeIerror_g <- function(result, alpha, ncase, ncontrol, maf){

  TypeIerror.SCORE.com <- mean(pchisq(result[, 1], 1, lower.tail=F) < alpha, na.rm=TRUE)
  TypeIerror.SCORE.mis1 <- mean(pchisq(result[, 2], 1, lower.tail=F) < alpha, na.rm=TRUE)
  TypeIerror.SCORE.mis2 <- mean(pchisq(result[, 3], 1, lower.tail=F) < alpha, na.rm=TRUE)
  TypeIerror.SCORE.med <- mean(pchisq(result[, 4], 1, lower.tail=F) < alpha, na.rm=TRUE)

  result.summary <- c(alpha, maf, ncase, ncontrol, TypeIerror.SCORE.com, TypeIerror.SCORE.mis1,
TypeIerror.SCORE.mis2, TypeIerror.SCORE.med)
  names(result.summary) <- c("Alpha", "MAF", "case","control", "TypeIerror.CST", "TypeIerror.PM1", "TypeIerror.PM2",
"TypeIerror.median")
  result.summary
}

# Calculate Score statistics for Power
CalcurateScoreStatistics_power <- function(R, seed, prevalence, beta0, beta1, beta2, ncase, ncontrol, maf, p.cov,
alpha, p.missing){

  score.cv <- numeric(R)
  score.pm1 <- numeric(R)
  score.pm2 <- numeric(R)
  score.med <- numeric(R)

  set.seed(seed)

  # r <- 1
  for (r in 1:R) {

    # print(r)

    beta0 <- as.numeric(ExploreBeta0(prevalence, beta1, beta2, maf, p.cov))
    sim.data <- CreateData(prevalence, beta0, beta1, beta2, ncase, ncontrol, maf, p.cov)
    missing.sim.data <- MissingData(sim.data, p.missing, ncase, ncontrol)

    # Conventional score test
    complete.data <-as.matrix(missing.sim.data[complete.cases(missing.sim.data), ])    #missing omit
```

```
    y.com <- complete.data[, 1]
    intercept.com <- rep(1, length(y.com))
    e.com <- complete.data[, 2]
    g.com <- complete.data[, 3]
    X.com <- cbind(intercept.com, e.com, g.com)
    X2.com <- cbind(g.com)

    glm.com <- f.glm.null(y.com, e.com)
    pi.com <- piFunc(glm.com[,1], X.com[,1:2])
    w.com <- as.numeric(pi.com * (1 - pi.com))
    I.com <- crossprod(X.com, w.com*X.com)
    iI.com <- solve(I.com)
    U.com <- t(X2.com) %*% (y.com - pi.com)
    score.cv[r] <- t(U.com) %*% iI.com[3, 3] %*% U.com


    # missing score statistics
    # Proposed method 1
    y.mis1 <- missing.sim.data[, 1]
    intercept.mis1 <- rep(1, length(y.mis1))
    e.mis1 <- missing.sim.data[, 2]
    g.mis1 <- missing.sim.data[, 3]
    g.mis1[is.na(g.mis1) == TRUE] <- 0    #if missing then 0
    X.mis1 <- cbind(intercept.mis1, e.mis1, g.mis1)
    X2.mis1 <- cbind(g.mis1)

    glm.mis1 <- f.glm.null(y.mis1, e.mis1)    #null model
    pi.mis1 <- piFunc(glm.mis1[,1], X.mis1[,1:2])
    w.mis1 <- as.numeric(pi.mis1 * (1 - pi.mis1))
    I.mis1 <- crossprod(X.mis1, w.mis1*X.mis1)
    iI.mis1 <- solve(I.mis1)
    U.mis1 <- t(X2.mis1) %*% (y.mis1 - pi.mis1)
    score.pm1[r] <- t(U.mis1) %*% iI.mis1[3, 3] %*% U.mis1


    # Proposed method 2
    inmis <- which(!is.na(missing.sim.data[, 3]))
    X.mis2 <- cbind(intercept.mis1, e.mis1, g.mis1)[inmis,]
    U.mis2 <-  t(X.mis2) %*% (y.mis1[inmis] - pi.mis1[inmis])    #(4, 1)
    I.mis2 <- crossprod(X.mis2, w.mis1[inmis]*X.mis2)    #(4, 4)

    iI.mis2.11 <- solve(I.mis2[1:2, 1:2])    #(3, 3)
    U.mis2.ast <- U.mis2[3] - I.mis2[3,1:2] %*% iI.mis2.11 %*% U.mis2[1:2]    #(1, 1)

    iImod.mis2 <- solve(-I.mis2[3,1:2] %*% iI.mis2.11 %*% I.mis2[1:2,3] + I.mis2[3,3])

    score.pm2[r] <- t(U.mis2.ast) %*% iImod.mis2 %*% U.mis2.ast


    # Median impute
    g.med <- missing.sim.data[, 3]
    median.g <- median(g.med, na.rm = TRUE)
    g.med[is.na(g.med) == TRUE] <- median.g    #if missing then 0
    X.med <- cbind(intercept.mis1, e.mis1, g.med)
    X2.med <- cbind(g.med)

    I.med <- crossprod(X.med, w.mis1*X.med)
    iI.med <- solve(I.med)
    U.med <- t(X2.med) %*% (y.mis1 - pi.mis1)
    score.med[r] <- t(U.med) %*% iI.med[3, 3] %*% U.med
  }
  result.score <- cbind(score.cv, score.pm1, score.pm2, score.med)

  TypeIerror.SCORE.cv <- mean(pchisq(result.score[, 1], 1, lower.tail=F) < alpha, na.rm=TRUE)
  TypeIerror.SCORE.pm1 <- mean(pchisq(result.score[, 2], 1, lower.tail=F) < alpha, na.rm=TRUE)
  TypeIerror.SCORE.pm2 <- mean(pchisq(result.score[, 3], 1, lower.tail=F) < alpha, na.rm=TRUE)
  TypeIerror.SCORE.med <- mean(pchisq(result.score[, 4], 1, lower.tail=F) < alpha, na.rm=TRUE)

  result.summary <- c(alpha, maf, ncase, exp(beta2), p.missing, TypeIerror.SCORE.cv, TypeIerror.SCORE.pm1,
TypeIerror.SCORE.pm2, TypeIerror.SCORE.med)
  names(result.summary) <- c("Alpha", "MAF", "case","beta2", "Missing","Power.CST", "Power.PM1", "Power.PM2",
"Power.median")
  result.summary

}

f.power.g <- function(OR2){
  beta2 <- log(OR2)
  CalcurateScoreStatistics_power(R, seed, prevalence, beta0, beta1, beta2, ncase, ncontrol, maf, p.cov, alpha,
p.missing)
}
```

```
# G-GE test --------------------------------------------------------------
#We explore the beta0 corresponding to the prevalence.
ExploreBeta0_ge<- function(prevalence, beta1, beta2, beta3, maf, p.cov){

  beta0 <- seq(-10, 0 , 0.0001)

  p <- (1-maf)^2*(1-p.cov)/(1+exp(-beta0)) +
    2*maf*(1-maf)*(1-p.cov)/(1+exp(-beta0-beta2)) +
    maf^2*(1-p.cov)/(1+exp(-beta0-2*beta2)) +
    (1-maf)^2*p.cov/(1+exp(-beta0-beta1)) +
    2*maf*(1-maf)*p.cov/(1+exp(-beta0-beta1-beta2-beta3)) +
    maf^2*p.cov/(1+exp(-beta0-beta1-2*beta2-2*beta3))

  diffp <- abs(prevalence - p)
  z <- cbind(beta0, p, diffp)
  mini.beta0 <- z[which.min(diffp),][1]
  mini.beta0
}


#We creat simulation data.
CreateData_ge <- function(prevalence, beta0, beta1, beta2, beta3, ncase, ncontrol, maf, p.cov){

  #set up
  row <- c(1,2,3,4,5,6)

  #case control sampling-----------------------------------------------------------------
  #y=1
  p_y1_a0b0 <- dbinom(0, 2, maf) * dbinom(0, 1, p.cov) * (1 / prevalence) * (1 / (1 + exp(-beta0 - beta1*0 - beta2*0
- beta3*0)))
  p_y1_a1b0 <- dbinom(1, 2, maf) * dbinom(0, 1, p.cov) * (1 / prevalence) * (1 / (1 + exp(-beta0 - beta1*0 - beta2*1
- beta3*0)))
  p_y1_a2b0 <- dbinom(2, 2, maf) * dbinom(0, 1, p.cov) * (1 / prevalence) * (1 / (1 + exp(-beta0 - beta1*0 - beta2*2
- beta3*0)))
  p_y1_a0b1 <- dbinom(0, 2, maf) * dbinom(1, 1, p.cov) * (1 / prevalence) * (1 / (1 + exp(-beta0 - beta1*1 - beta2*0
- beta3*0)))
  p_y1_a1b1 <- dbinom(1, 2, maf) * dbinom(1, 1, p.cov) * (1 / prevalence) * (1 / (1 + exp(-beta0 - beta1*1 - beta2*1
- beta3*1)))
  p_y1_a2b1 <- dbinom(2, 2, maf) * dbinom(1, 1, p.cov) * (1 / prevalence) * (1 / (1 + exp(-beta0 - beta1*1 - beta2*2
- beta3*2)))
  p_y1 <- c(p_y1_a0b0, p_y1_a1b0, p_y1_a2b0, p_y1_a0b1, p_y1_a1b1, p_y1_a2b1)

  x1_y1 <- numeric(ncase)
  x2_y1 <- numeric(ncase)
  u1 <- t(row %*% rmultinom(ncase, 1, p_y1))

  x1_y1[u1==1] <- 0
  x1_y1[u1==2] <- 0
  x1_y1[u1==3] <- 0
  x1_y1[u1==4] <- 1
  x1_y1[u1==5] <- 1
  x1_y1[u1==6] <- 1

  x2_y1[u1==1] <- 0
  x2_y1[u1==2] <- 1
  x2_y1[u1==3] <- 2
  x2_y1[u1==4] <- 0
  x2_y1[u1==5] <- 1
  x2_y1[u1==6] <- 2

  y_y1 <- rep(1, ncase)

  #y=0
  p_y0_a0b0 <- dbinom(0 ,2, maf) * dbinom(0, 1, p.cov) * (1 / (1 - prevalence)) * (exp(-beta0 - beta1*0 - beta2*0 -
beta3*0) / (1 + exp(-beta0 - beta1*0 - beta2*0 - beta3*0)))
  p_y0_a1b0 <- dbinom(1 ,2, maf) * dbinom(0, 1, p.cov) * (1 / (1 - prevalence)) * (exp(-beta0 - beta1*0 - beta2*1 -
beta3*0) / (1 + exp(-beta0 - beta1*0 - beta2*1 - beta3*0)))
  p_y0_a2b0 <- dbinom(2 ,2, maf) * dbinom(0, 1, p.cov) * (1 / (1 - prevalence)) * (exp(-beta0 - beta1*0 - beta2*2 -
beta3*0) / (1 + exp(-beta0 - beta1*0 - beta2*2 - beta3*0)))
  p_y0_a0b1 <- dbinom(0 ,2, maf) * dbinom(1, 1, p.cov) * (1 / (1 - prevalence)) * (exp(-beta0 - beta1*1 - beta2*0 -
beta3*0) / (1 + exp(-beta0 - beta1*1 - beta2*0 - beta3*0)))
  p_y0_a1b1 <- dbinom(1 ,2, maf) * dbinom(1, 1, p.cov) * (1 / (1 - prevalence)) * (exp(-beta0 - beta1*1 - beta2*1 -
beta3*1) / (1 + exp(-beta0 - beta1*1 - beta2*1 - beta3*1)))
  p_y0_a2b1 <- dbinom(2 ,2, maf) * dbinom(1, 1, p.cov) * (1 / (1 - prevalence)) * (exp(-beta0 - beta1*1 - beta2*2 -
beta3*2) / (1 + exp(-beta0 - beta1*1 - beta2*2 - beta3*2)))
  p_y0<- c(p_y0_a0b0, p_y0_a1b0, p_y0_a2b0, p_y0_a0b1, p_y0_a1b1, p_y0_a2b1)

  x1_y0 <- numeric(ncontrol)
  x2_y0 <- numeric(ncontrol)
  u0 <- t(row %*% rmultinom(ncontrol,1,p_y0))

  x1_y0[u0==1] <- 0
  x1_y0[u0==2] <- 0
  x1_y0[u0==3] <- 0
  x1_y0[u0==4] <- 1
```

```
  x1_y0[u0==5] <- 1
  x1_y0[u0==6] <- 1

  x2_y0[u0==1] <- 0
  x2_y0[u0==2] <- 1
  x2_y0[u0==3] <- 2
  x2_y0[u0==4] <- 0
  x2_y0[u0==5] <- 1
  x2_y0[u0==6] <- 2

  y_y0 <- rep(0, ncontrol)

  e <- c(x1_y1, x1_y0)  #snp
  g <- c(x2_y1, x2_y0)  #covariate
  ge <- g*e             #gene-environment factor
  y <- c(y_y1, y_y0)
  simulation.data <- cbind(y, e, g, ge)
  simulation.data
}

# Create missing data for SNP(g).
MissingData_ge <- function(sim.data, p.missing, ncase, ncontrol){
  ndata <- ncase + ncontrol
  sim.data[, 3][runif(ndata) < p.missing] <- NA    #g <- missing
  sim.data[, 4] <- sim.data[, 2] * sim.data[, 3]    #ge <- missing
  sim.data
}

# Calculate Score statistics
CalcurateScoreStatistics_ge <- function(R, seed, prevalence, beta0, beta1, beta2, beta3, ncase, ncontrol, maf, p.cov)
{

  score.cv <- numeric(R)
  score.pm1 <- numeric(R)
  score.pm2 <- numeric(R)
  score.med <- numeric(R)

  set.seed(seed)

  for (r in 1:R) {
    beta0 <- as.numeric(ExploreBeta0_ge(prevalence, beta1, beta2, beta3, maf, p.cov))
    sim.data <- CreateData_ge(prevalence, beta0, beta1, beta2, beta3, ncase, ncontrol, maf, p.cov)
    missing.sim.data <- MissingData_ge(sim.data, p.missing, ncase, ncontrol)

    # Conventional score test
    complete.data <-as.matrix(missing.sim.data[complete.cases(missing.sim.data), ])    #missing omit
    y.com <- complete.data[, 1]
    intercept.com <- rep(1, length(y.com))
    e.com <- complete.data[, 2]
    g.com <- complete.data[, 3]
    ge.com <- complete.data[, 4]
    X.com <- cbind(intercept.com, e.com, g.com, ge.com)
    X2.com <- cbind(g.com, ge.com)

    glm.com <- f.glm.null(y.com, e.com)
    pi.com <- piFunc(glm.com[,1], X.com[,1:2])
    w.com <- as.numeric(pi.com * (1 - pi.com))
    I.com <- crossprod(X.com, w.com*X.com)
    iI.com <- solve(I.com)
    U.com <- t(X2.com) %*% (y.com - pi.com)
    score.cv[r] <- t(U.com) %*% iI.com[3:4, 3:4] %*% U.com


    # Proposed method 1
    y.mis1 <- missing.sim.data[, 1]
    intercept.mis1 <- rep(1, length(y.mis1))
    e.mis1 <- missing.sim.data[, 2]
    g.mis1 <- missing.sim.data[, 3]
    ge.mis1 <- missing.sim.data[, 4]
    g.mis1[is.na(g.mis1) == TRUE] <- 0    #if missing then 0
    ge.mis1[is.na(ge.mis1) == TRUE] <- 0    #if missing then 0
    X.mis1 <- cbind(intercept.mis1, e.mis1, g.mis1, ge.mis1)
    X2.mis1 <- cbind(g.mis1, ge.mis1)

    glm.mis1 <- f.glm.null(y.mis1, e.mis1)    #null model
    pi.mis1 <- piFunc(glm.mis1[,1], X.mis1[,1:2])
    w.mis1 <- as.numeric(pi.mis1 * (1 - pi.mis1))
    I.mis1 <- crossprod(X.mis1, w.mis1*X.mis1)
    iI.mis1 <- solve(I.mis1)
    U.mis1 <- t(X2.mis1) %*% (y.mis1 - pi.mis1)
    score.pm1[r] <- t(U.mis1) %*% iI.mis1[3:4, 3:4] %*% U.mis1


    # Proposed method 2
    inmis <- which(!is.na(missing.sim.data[, 3]))
```

```
    X.mis2 <- cbind(intercept.mis1, e.mis1, g.mis1, ge.mis1)[inmis,]
    U.mis2 <-  t(X.mis2) %*% (y.mis1[inmis] - pi.mis1[inmis])    #(4, 1)
    I.mis2 <- crossprod(X.mis2, w.mis1[inmis]*X.mis2)    #(4, 4)

    iI.mis2.11 <- solve(I.mis2[1:2, 1:2])    #(3, 3)
    U.mis2.ast <- U.mis2[3:4] - I.mis2[3:4, 1:2] %*% iI.mis2.11 %*% U.mis2[1:2]    #(1, 1)

    iImod.mis2 <- solve(-I.mis2[3:4, 1:2] %*% iI.mis2.11 %*% I.mis2[1:2, 3:4] + I.mis2[3:4, 3:4])

    score.pm2[r] <- t(U.mis2.ast) %*% iImod.mis2 %*% U.mis2.ast


    # Median imputation
    g.med <- missing.sim.data[, 3]
    ge.med <- missing.sim.data[, 4]
    median.g <- median(g.med, na.rm = TRUE)
    g.med[is.na(g.med) == TRUE] <- median.g    #if missing then 0
    ge.med <- e.mis1 * g.med
    X.med <- cbind(intercept.mis1, e.mis1, g.med, ge.med)
    X2.med <- cbind(g.med, ge.med)

    I.med <- crossprod(X.med, w.mis1*X.med)
    iI.med <- solve(I.med)
    U.med <- t(X2.med) %*% (y.mis1 - pi.mis1)
    score.med[r] <- t(U.med) %*% iI.med[3:4, 3:4] %*% U.med
  }
  result.score <- cbind(score.cv, score.pm1, score.pm2, score.med)
  result.score
}


# Calculate Type I error.
CalculateTypeIerror_ge <- function(result, alpha, ncase, ncontrol, maf){

  TypeIerror.SCORE.com <- mean(pchisq(result[, 1], 2, lower.tail=F) < alpha, na.rm=TRUE)
  TypeIerror.SCORE.mis1 <- mean(pchisq(result[, 2], 2, lower.tail=F) < alpha, na.rm=TRUE)
  TypeIerror.SCORE.mis2 <- mean(pchisq(result[, 3], 2, lower.tail=F) < alpha, na.rm=TRUE)
  TypeIerror.SCORE.med <- mean(pchisq(result[, 4], 2, lower.tail=F) < alpha, na.rm=TRUE)

  result.summary <- c(alpha, maf, ncase, ncontrol, TypeIerror.SCORE.com, TypeIerror.SCORE.mis1,
TypeIerror.SCORE.mis2, TypeIerror.SCORE.med)
  names(result.summary) <- c("Alpha", "MAF", "case","control", "TypeIerror.CST", "TypeIerror.PM1", "TypeIerror.PM2",
"TypeIerror.median")
  result.summary
}

# Calculate Score statistics for Power
CalcurateScoreStatistics_ge_power <- function(R, seed, prevalence, beta0, beta1, beta2, beta3, ncase, ncontrol, maf,
p.cov, alpha, p.missing){

  score.cv <- numeric(R)
  score.pm1 <- numeric(R)
  score.pm2 <- numeric(R)
  score.med <- numeric(R)

  set.seed(seed)

  # r <- 1
  for (r in 1:R) {

    beta0 <- as.numeric(ExploreBeta0_ge(prevalence, beta1, beta2, beta3, maf, p.cov))
    sim.data <- CreateData_ge(prevalence, beta0, beta1, beta2, beta3, ncase, ncontrol, maf, p.cov)
    missing.sim.data <- MissingData_ge(sim.data, p.missing, ncase, ncontrol)

    # Conventional score test
    complete.data <-as.matrix(missing.sim.data[complete.cases(missing.sim.data), ])    #missing omit
    y.com <- complete.data[, 1]
    intercept.com <- rep(1, length(y.com))
    e.com <- complete.data[, 2]
    g.com <- complete.data[, 3]
    ge.com <- complete.data[, 4]
    X.com <- cbind(intercept.com, e.com, g.com, ge.com)
    X2.com <- cbind(g.com, ge.com)

    glm.com <- f.glm.null(y.com, e.com)
    pi.com <- piFunc(glm.com[,1], X.com[,1:2])
    w.com <- as.numeric(pi.com * (1 - pi.com))
    I.com <- crossprod(X.com, w.com*X.com)
    iI.com <- solve(I.com)
    U.com <- t(X2.com) %*% (y.com - pi.com)
    score.cv[r] <- t(U.com) %*% iI.com[3:4, 3:4] %*% U.com


    # missing score statistics
    # Proposed method 1
```

```
    y.mis1 <- missing.sim.data[, 1]
    intercept.mis1 <- rep(1, length(y.mis1))
    e.mis1 <- missing.sim.data[, 2]
    g.mis1 <- missing.sim.data[, 3]
    ge.mis1 <- missing.sim.data[, 4]
    g.mis1[is.na(g.mis1) == TRUE] <- 0    #if missing then 0
    ge.mis1[is.na(ge.mis1) == TRUE] <- 0    #if missing then 0
    X.mis1 <- cbind(intercept.mis1, e.mis1, g.mis1, ge.mis1)
    X2.mis1 <- cbind(g.mis1, ge.mis1)

    glm.mis1 <- f.glm.null(y.mis1, e.mis1)    #null model
    pi.mis1 <- piFunc(glm.mis1[,1], X.mis1[,1:2])
    w.mis1 <- as.numeric(pi.mis1 * (1 - pi.mis1))
    I.mis1 <- crossprod(X.mis1, w.mis1*X.mis1)
    iI.mis1 <- solve(I.mis1)
    U.mis1 <- t(X2.mis1) %*% (y.mis1 - pi.mis1)
    score.pm1[r] <- t(U.mis1) %*% iI.mis1[3:4, 3:4] %*% U.mis1


    # Proposed method 2
    inmis <- which(!is.na(missing.sim.data[, 3]))
    X.mis2 <- cbind(intercept.mis1, e.mis1, g.mis1, ge.mis1)[inmis,]
    U.mis2 <-  t(X.mis2) %*% (y.mis1[inmis] - pi.mis1[inmis])    #(4, 1)
    I.mis2 <- crossprod(X.mis2, w.mis1[inmis]*X.mis2)    #(4, 4)

    iI.mis2.11 <- solve(I.mis2[1:2, 1:2])    #(3, 3)
    U.mis2.ast <- U.mis2[3:4] - I.mis2[3:4, 1:2] %*% iI.mis2.11 %*% U.mis2[1:2]    #(1, 1)

    iImod.mis2 <- solve(-I.mis2[3:4, 1:2] %*% iI.mis2.11 %*% I.mis2[1:2, 3:4] + I.mis2[3:4, 3:4])

    score.pm2[r] <- t(U.mis2.ast) %*% iImod.mis2 %*% U.mis2.ast


    # Median impute
    g.med <- missing.sim.data[, 3]
    ge.med <- missing.sim.data[, 4]
    median.g <- median(g.med, na.rm = TRUE)
    g.med[is.na(g.med) == TRUE] <- median.g    #if missing then 0
    ge.med <- e.mis1 * g.med
    X.med <- cbind(intercept.mis1, e.mis1, g.med, ge.med)
    X2.med <- cbind(g.med, ge.med)

    I.med <- crossprod(X.med, w.mis1*X.med)
    iI.med <- solve(I.med)
    U.med <- t(X2.med) %*% (y.mis1 - pi.mis1)
    score.med[r] <- t(U.med) %*% iI.med[3:4, 3:4] %*% U.med
  }
  result.score <- cbind(score.cv, score.pm1, score.pm2, score.med)

  TypeIerror.SCORE.cv <- mean(pchisq(result.score[, 1], 2, lower.tail=F) < alpha, na.rm=TRUE)
  TypeIerror.SCORE.pm1 <- mean(pchisq(result.score[, 2], 2, lower.tail=F) < alpha, na.rm=TRUE)
  TypeIerror.SCORE.pm2 <- mean(pchisq(result.score[, 3], 2, lower.tail=F) < alpha, na.rm=TRUE)
  TypeIerror.SCORE.med <- mean(pchisq(result.score[, 4], 2, lower.tail=F) < alpha, na.rm=TRUE)

  result.summary <- c(alpha, maf, ncase, exp(beta2), exp(beta3), p.missing, TypeIerror.SCORE.cv,
TypeIerror.SCORE.pm1, TypeIerror.SCORE.pm2, TypeIerror.SCORE.med)
  names(result.summary) <- c("Alpha", "MAF", "case", "beta2", "beta3", "Missing","Power.CST", "Power.PM1",
"Power.PM2", "Power.median")
  result.summary
}

f.power.ge <- function(OR3){
  beta3 <- log(OR3)
  CalcurateScoreStatistics_ge_power(R, seed, prevalence, beta0, beta1, beta2, beta3, ncase, ncontrol, maf, p.cov,
alpha, p.missing)
}
```
