# Supplementary figures and images for: Fast score test with global null estimation regardless of missing genotypes
Source: PLoS One. 2018 Jul 5;13(7):e0199692. doi: 10.1371/journal.pone.0199692 (PMC6033421; doi:10.1371/journal.pone.0199692)

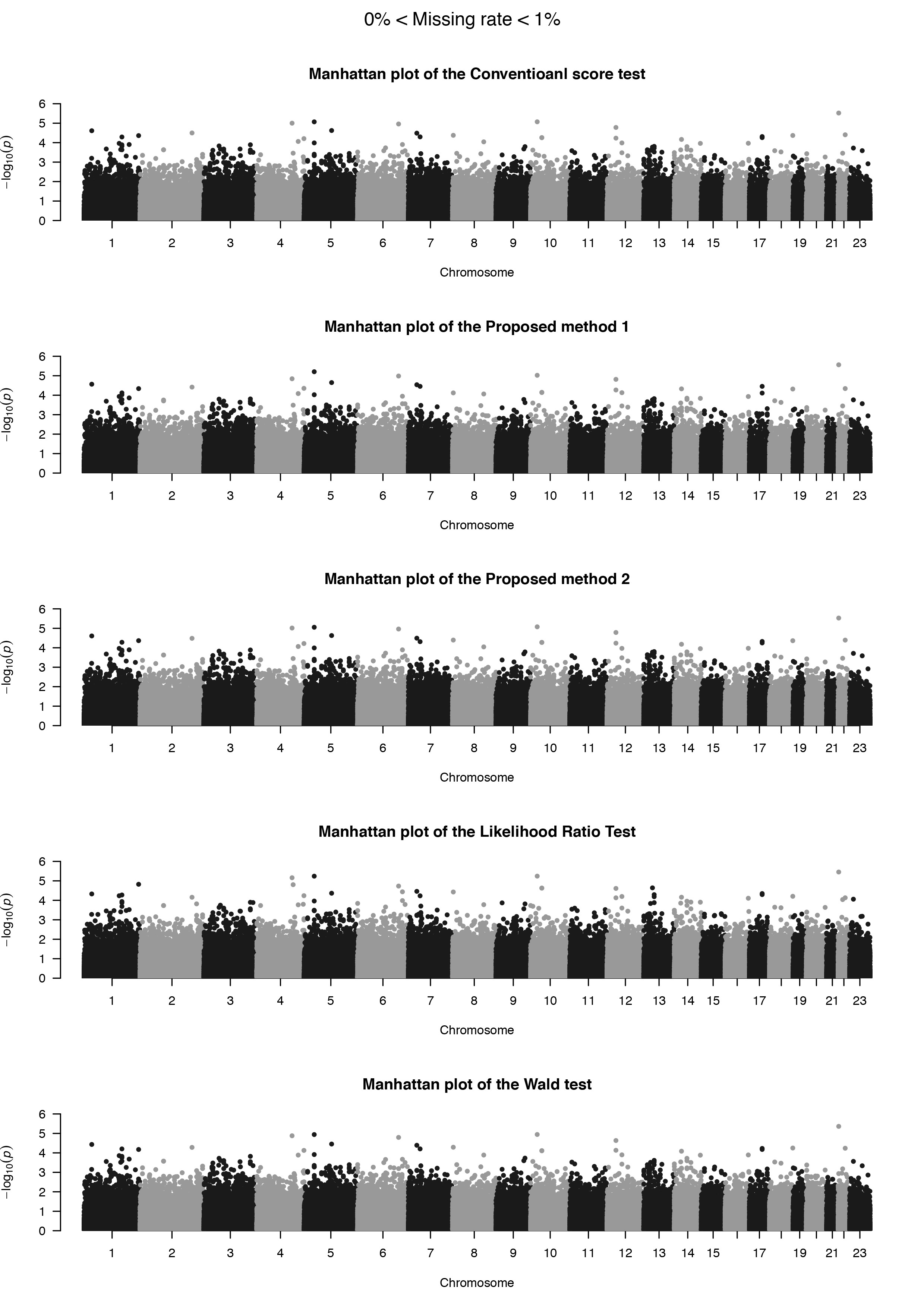

Supplement: S7 Fig — The y-axis denotes the number of SNPs. The x-axis denotes Missing rate. Low missing population include SNPs with missing (0% < Missing rate < 1%). (PNG) [file pone.0199692.s009.png]

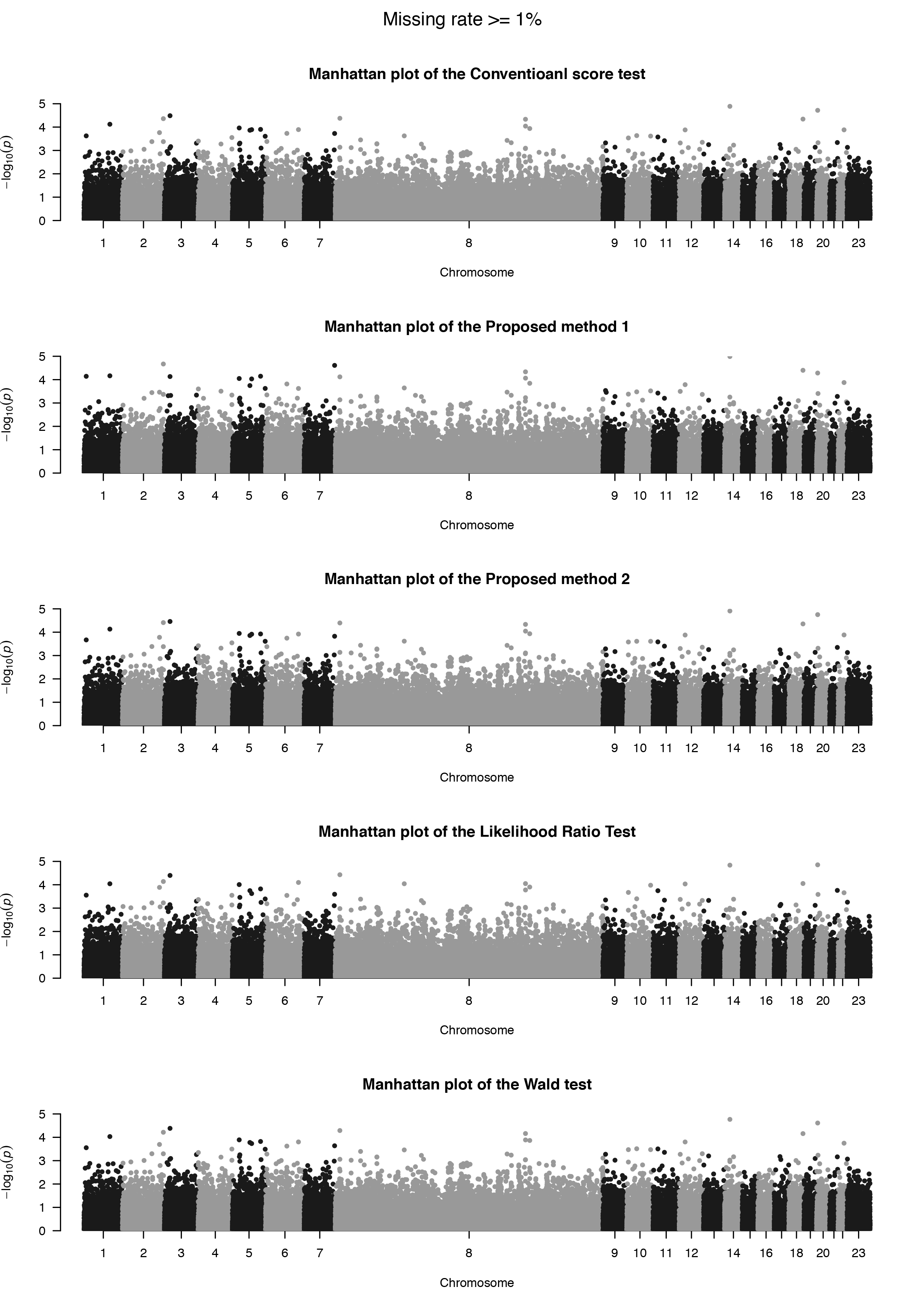

Supplement: S8 Fig — The y-axis denotes the number of SNPs. The x-axis denotes Missing rate. High missing population include SNPs with missing (Missing rate ≥ 1%). (PNG) [file pone.0199692.s010.png]
