# Supplementary material for: Fast score test with global null estimation regardless of missing genotypes
Source: PLoS One. 2018 Jul 5;13(7):e0199692. doi: 10.1371/journal.pone.0199692 (PMC6033421; doi:10.1371/journal.pone.0199692)
Supplement: S1 Table — Type I error rates of the conventional score test (CST), the proposed method 1 (PM1), and the proposed method 2 (PM2) at a significance level of α = 5 × 10^−5^. (PDF) [file pone.0199692.s011.pdf]

Test	Missing rate (%)	MAF	#case/control	CST	PM1	PM2
G	2	10	100/100	3.20E-05	3.30E-05	3.20E-05
G	2	10	500/500	4.30E-05	4.10E-05	4.30E-05
G	2	30	100/100	5.00E-05	4.70E-05	4.90E-05
G	2	30	500/500	5.30E-05	5.30E-05	5.30E-05
G	5	10	100/100	2.70E-05	2.70E-05	2.60E-05
G	5	10	500/500	4.00E-05	3.80E-05	4.00E-05
G	5	30	100/100	4.40E-05	4.80E-05	4.50E-05
G	5	30	500/500	5.40E-05	4.60E-05	5.40E-05
G	10	10	100/100	2.50E-05	2.00E-05	2.40E-05
G	10	10	500/500	3.50E-05	4.10E-05	3.40E-05
G	10	30	100/100	4.60E-05	4.50E-05	4.60E-05
G	10	30	500/500	5.30E-05	4.80E-05	5.30E-05
G	2	10	1000/2000	5.10E-05	5.50E-05	5.10E-05
G	2	30	1000/2000	4.70E-05	4.60E-05	4.60E-05
G	5	10	1000/2000	4.80E-05	5.00E-05	4.80E-05
G	5	30	1000/2000	5.00E-05	4.10E-05	5.10E-05
G	10	10	1000/2000	4.20E-05	4.40E-05	4.30E-05
G	10	30	1000/2000	4.40E-05	4.70E-05	4.40E-05
G-GE	2	10	100/100	2.20E-05	2.10E-05	2.20E-05
G-GE	2	10	500/500	2.50E-05	2.40E-05	2.50E-05
G-GE	2	30	100/100	3.70E-05	3.70E-05	3.70E-05
G-GE	2	30	500/500	5.40E-05	4.90E-05	5.30E-05
G-GE	5	10	100/100	1.70E-05	1.60E-05	1.70E-05
G-GE	5	10	500/500	3.10E-05	3.00E-05	3.10E-05
G-GE	5	30	100/100	4.40E-05	3.10E-05	4.40E-05
G-GE	5	30	500/500	5.80E-05	4.80E-05	5.80E-05
G-GE	10	10	100/100	1.70E-05	2.10E-05	1.70E-05
G-GE	10	10	500/500	2.50E-05	2.30E-05	2.50E-05
G-GE	10	30	100/100	3.30E-05	2.90E-05	3.30E-05
G-GE	10	30	500/500	6.00E-05	4.80E-05	6.00E-05
G-GE	2	10	1000/2000	5.10E-05	4.90E-05	5.00E-05
G-GE	2	30	1000/2000	4.50E-05	4.50E-05	4.50E-05
G-GE	5	10	1000/2000	5.10E-05	4.80E-05	5.00E-05
G-GE	5	30	1000/2000	4.00E-05	4.40E-05	4.00E-05
G-GE	10	10	1000/2000	5.00E-05	4.40E-05	5.00E-05
G-GE	10	30	1000/2000	5.10E-05	4.60E-05	5.20E-05
