# Supplementary material for: Fast score test with global null estimation regardless of missing genotypes
Source: PLoS One. 2018 Jul 5;13(7):e0199692. doi: 10.1371/journal.pone.0199692 (PMC6033421; doi:10.1371/journal.pone.0199692)
Supplement: S2 Table — G test Power of the conventional score test (CST), the proposed method 1 (PM1), and the proposed method 2 (PM2) under missing rate (2%, 5%, 10%, 30%), minor allele frequency (MAF) (10%, 30%), and the number of case/control (1,000, 5,000). The x-axis denotes genetic odds ratios (ORg = exp(βg)). The significance level is 5 × 10^−8^. G-GE test Power of CST, PM1, and PM2 under genetic odds ratios (ORg = exp(βg) = 1.1, 1.2), missing rate (2%, 5%, 10%, 30%), minor allele frequency (MAF) (10%, 30%), and the number of case/control is 1,000. The x-axis denotes gene-environment interaction odds ratios (ORge = exp(βge)). The significance level is 5 × 10^−8^. (PDF) [file pone.0199692.s012.pdf]

Test	Alpha	MAF	#case/control	beta2	beta3	Missing rate	CST	PM1	PM2	median imputation
G	5.00E-08	0.1	1000/1000	1	-	0.02	0	0	0	0
G	5.00E-08	0.1	1000/1000	1.1	-	0.02	0	0	0	0
G	5.00E-08	0.1	1000/1000	1.2	-	0.02	0	0	0	0
G	5.00E-08	0.1	1000/1000	1.3	-	0.02	0.001	0.001	0.001	0.001
G	5.00E-08	0.1	1000/1000	1.4	-	0.02	0.015	0.015	0.015	0.015
G	5.00E-08	0.1	1000/1000	1.5	-	0.02	0.085	0.085	0.085	0.085
G	5.00E-08	0.1	5000/5000	1	-	0.02	0	0	0	0
G	5.00E-08	0.1	5000/5000	1.1	-	0.02	0	0	0	0
G	5.00E-08	0.1	5000/5000	1.2	-	0.02	0.057	0.054	0.057	0.054
G	5.00E-08	0.1	5000/5000	1.3	-	0.02	0.629	0.625	0.629	0.625
G	5.00E-08	0.1	5000/5000	1.4	-	0.02	0.981	0.979	0.981	0.979
G	5.00E-08	0.1	5000/5000	1.5	-	0.02	1	1	1	1
G	5.00E-08	0.3	1000/1000	1	-	0.02	0	0	0	0
G	5.00E-08	0.3	1000/1000	1.1	-	0.02	0	0	0	0
G	5.00E-08	0.3	1000/1000	1.2	-	0.02	0.002	0.001	0.002	0.003
G	5.00E-08	0.3	1000/1000	1.3	-	0.02	0.063	0.054	0.063	0.064
G	5.00E-08	0.3	1000/1000	1.4	-	0.02	0.287	0.264	0.287	0.283
G	5.00E-08	0.3	1000/1000	1.5	-	0.02	0.701	0.688	0.702	0.7
G	5.00E-08	0.3	5000/5000	1	-	0.02	0	0	0	0
G	5.00E-08	0.3	5000/5000	1.1	-	0.02	0.015	0.014	0.015	0.013
G	5.00E-08	0.3	5000/5000	1.2	-	0.02	0.708	0.678	0.708	0.703
G	5.00E-08	0.3	5000/5000	1.3	-	0.02	0.999	0.999	0.999	0.999
G	5.00E-08	0.3	5000/5000	1.4	-	0.02	1	1	1	1
G	5.00E-08	0.3	5000/5000	1.5	-	0.02	1	1	1	1
G	5.00E-08	0.1	1000/1000	1	-	0.05	0	0	0	0
G	5.00E-08	0.1	1000/1000	1.1	-	0.05	0	0	0	0
G	5.00E-08	0.1	1000/1000	1.2	-	0.05	0	0	0	0
G	5.00E-08	0.1	1000/1000	1.3	-	0.05	0.001	0.001	0.001	0.001
G	5.00E-08	0.1	1000/1000	1.4	-	0.05	0.014	0.015	0.014	0.015
G	5.00E-08	0.1	1000/1000	1.5	-	0.05	0.077	0.075	0.077	0.075
G	5.00E-08	0.1	5000/5000	1	-	0.05	0	0	0	0
G	5.00E-08	0.1	5000/5000	1.1	-	0.05	0	0	0	0
G	5.00E-08	0.1	5000/5000	1.2	-	0.05	0.049	0.05	0.049	0.05
G	5.00E-08	0.1	5000/5000	1.3	-	0.05	0.589	0.572	0.589	0.572
G	5.00E-08	0.1	5000/5000	1.4	-	0.05	0.975	0.972	0.975	0.972
G	5.00E-08	0.1	5000/5000	1.5	-	0.05	1	1	1	1
G	5.00E-08	0.3	1000/1000	1	-	0.05	0	0	0	0
G	5.00E-08	0.3	1000/1000	1.1	-	0.05	0	0	0	0
G	5.00E-08	0.3	1000/1000	1.2	-	0.05	0.001	0.001	0.001	0.002
G	5.00E-08	0.3	1000/1000	1.3	-	0.05	0.057	0.048	0.057	0.054
G	5.00E-08	0.3	1000/1000	1.4	-	0.05	0.257	0.22	0.258	0.253
G	5.00E-08	0.3	1000/1000	1.5	-	0.05	0.671	0.608	0.671	0.661
G	5.00E-08	0.3	5000/5000	1	-	0.05	0	0	0	0
G	5.00E-08	0.3	5000/5000	1.1	-	0.05	0.013	0.012	0.012	0.009
G	5.00E-08	0.3	5000/5000	1.2	-	0.05	0.674	0.621	0.674	0.662
G	5.00E-08	0.3	5000/5000	1.3	-	0.05	0.997	0.995	0.997	0.998
G	5.00E-08	0.3	5000/5000	1.4	-	0.05	1	1	1	1
G	5.00E-08	0.3	5000/5000	1.5	-	0.05	1	1	1	1
G	5.00E-08	0.1	1000/1000	1	-	0.1	0	0	0	0
G	5.00E-08	0.1	1000/1000	1.1	-	0.1	0	0	0	0
G	5.00E-08	0.1	1000/1000	1.2	-	0.1	0	0	0	0
G	5.00E-08	0.1	1000/1000	1.3	-	0.1	0	0.001	0	0.001
G	5.00E-08	0.1	1000/1000	1.4	-	0.1	0.01	0.006	0.01	0.006
G	5.00E-08	0.1	1000/1000	1.5	-	0.1	0.062	0.055	0.062	0.055
G	5.00E-08	0.1	5000/5000	1	-	0.1	0	0	0	0
G	5.00E-08	0.1	5000/5000	1.1	-	0.1	0	0	0	0
G	5.00E-08	0.1	5000/5000	1.2	-	0.1	0.044	0.039	0.044	0.039
G	5.00E-08	0.1	5000/5000	1.3	-	0.1	0.541	0.496	0.541	0.496
G	5.00E-08	0.1	5000/5000	1.4	-	0.1	0.965	0.963	0.965	0.963
G	5.00E-08	0.1	5000/5000	1.5	-	0.1	1	1	1	1

Test	Alpha	MAF	#case/control	beta2	beta3	Missing rate	CST	PM1	PM2	median imputation
G	5.00E-08	0.3	1000/1000	1	-	0.1	0	0	0	0
G	5.00E-08	0.3	1000/1000	1.1	-	0.1	0	0	0	0
G	5.00E-08	0.3	1000/1000	1.2	-	0.1	0.001	0.001	0.001	0.001
G	5.00E-08	0.3	1000/1000	1.3	-	0.1	0.043	0.026	0.043	0.035
G	5.00E-08	0.3	1000/1000	1.4	-	0.1	0.216	0.158	0.215	0.2
G	5.00E-08	0.3	1000/1000	1.5	-	0.1	0.614	0.482	0.614	0.6
G	5.00E-08	0.3	5000/5000	1	-	0.1	0	0	0	0
G	5.00E-08	0.3	5000/5000	1.1	-	0.1	0.012	0.01	0.012	0.01
G	5.00E-08	0.3	5000/5000	1.2	-	0.1	0.625	0.518	0.626	0.591
G	5.00E-08	0.3	5000/5000	1.3	-	0.1	0.997	0.987	0.997	0.996
G	5.00E-08	0.3	5000/5000	1.4	-	0.1	1	1	1	1
G	5.00E-08	0.3	5000/5000	1.5	-	0.1	1	1	1	1
G	5.00E-08	0.1	1000/1000	1	-	0.3	0	0	0	0
G	5.00E-08	0.1	1000/1000	1.1	-	0.3	0	0	0	0
G	5.00E-08	0.1	1000/1000	1.2	-	0.3	0	0	0	0
G	5.00E-08	0.1	1000/1000	1.3	-	0.3	0	0	0	0
G	5.00E-08	0.1	1000/1000	1.4	-	0.3	0.006	0	0.006	0
G	5.00E-08	0.1	1000/1000	1.5	-	0.3	0.024	0.015	0.024	0.015
G	5.00E-08	0.1	5000/5000	1	-	0.3	0	0	0	0
G	5.00E-08	0.1	5000/5000	1.1	-	0.3	0	0	0	0
G	5.00E-08	0.1	5000/5000	1.2	-	0.3	0.024	0.022	0.024	0.022
G	5.00E-08	0.1	5000/5000	1.3	-	0.3	0.276	0.23	0.276	0.23
G	5.00E-08	0.1	5000/5000	1.4	-	0.3	0.812	0.745	0.812	0.745
G	5.00E-08	0.1	5000/5000	1.5	-	0.3	0.992	0.985	0.992	0.985
G	5.00E-08	0.3	1000/1000	1	-	0.3	0	0	0	0
G	5.00E-08	0.3	1000/1000	1.1	-	0.3	0	0	0	0
G	5.00E-08	0.3	1000/1000	1.2	-	0.3	0	0	0	0
G	5.00E-08	0.3	1000/1000	1.3	-	0.3	0.017	0.006	0.017	0.013
G	5.00E-08	0.3	1000/1000	1.4	-	0.3	0.09	0.035	0.09	0.078
G	5.00E-08	0.3	1000/1000	1.5	-	0.3	0.337	0.156	0.336	0.289
G	5.00E-08	0.3	5000/5000	1	-	0.3	0	0	0	0
G	5.00E-08	0.3	5000/5000	1.1	-	0.3	0.006	0.001	0.006	0.001
G	5.00E-08	0.3	5000/5000	1.2	-	0.3	0.347	0.17	0.346	0.272
G	5.00E-08	0.3	5000/5000	1.3	-	0.3	0.973	0.836	0.973	0.95
G	5.00E-08	0.3	5000/5000	1.4	-	0.3	1	1	1	1
G	5.00E-08	0.3	5000/5000	1.5	-	0.3	1	1	1	1
G-GE	5.00E-08	0.1	1000/1000	1.1	1	0.02	0	0	0	0
G-GE	5.00E-08	0.1	1000/1000	1.1	1.1	0.02	0	0	0	0
G-GE	5.00E-08	0.1	1000/1000	1.1	1.2	0.02	0	0	0	0
G-GE	5.00E-08	0.1	1000/1000	1.1	1.3	0.02	0.005	0.005	0.005	0.005
G-GE	5.00E-08	0.1	1000/1000	1.1	1.4	0.02	0.015	0.016	0.015	0.016
G-GE	5.00E-08	0.1	1000/1000	1.1	1.5	0.02	0.06	0.059	0.06	0.059
G-GE	5.00E-08	0.1	1000/1000	1.2	1	0.02	0	0	0	0
G-GE	5.00E-08	0.1	1000/1000	1.2	1.1	0.02	0.005	0.005	0.005	0.005
G-GE	5.00E-08	0.1	1000/1000	1.2	1.2	0.02	0.006	0.006	0.006	0.006
G-GE	5.00E-08	0.1	1000/1000	1.2	1.3	0.02	0.024	0.021	0.024	0.021
G-GE	5.00E-08	0.1	1000/1000	1.2	1.4	0.02	0.081	0.074	0.081	0.074
G-GE	5.00E-08	0.1	1000/1000	1.2	1.5	0.02	0.206	0.202	0.206	0.202
G-GE	5.00E-08	0.1	5000/5000	1.1	1	0.02	0	0	0	0
G-GE	5.00E-08	0.1	5000/5000	1.1	1.1	0.02	0.014	0.014	0.014	0.014
G-GE	5.00E-08	0.1	5000/5000	1.1	1.2	0.02	0.229	0.221	0.229	0.221
G-GE	5.00E-08	0.1	5000/5000	1.1	1.3	0.02	0.734	0.737	0.734	0.737
G-GE	5.00E-08	0.1	5000/5000	1.1	1.4	0.02	0.967	0.963	0.967	0.963
G-GE	5.00E-08	0.1	5000/5000	1.1	1.5	0.02	0.999	0.999	0.999	0.999
G-GE	5.00E-08	0.1	5000/5000	1.2	1	0.02	0.072	0.068	0.072	0.068
G-GE	5.00E-08	0.1	5000/5000	1.2	1.1	0.02	0.443	0.442	0.443	0.442
G-GE	5.00E-08	0.1	5000/5000	1.2	1.2	0.02	0.885	0.879	0.885	0.879
G-GE	5.00E-08	0.1	5000/5000	1.2	1.3	0.02	0.996	0.995	0.996	0.995
G-GE	5.00E-08	0.1	5000/5000	1.2	1.4	0.02	1	1	1	1
G-GE	5.00E-08	0.1	5000/5000	1.2	1.5	0.02	1	1	1	1

Test	Alpha	MAF	#case/control	beta2	beta3	Missing rate	CST	PM1	PM2	median imputation
G-GE	5.00E-08	0.3	1000/1000	1.1	1	0.02	0	0	0	0
G-GE	5.00E-08	0.3	1000/1000	1.1	1.1	0.02	0.001	0.002	0.001	0.001
G-GE	5.00E-08	0.3	1000/1000	1.1	1.2	0.02	0.018	0.016	0.018	0.019
G-GE	5.00E-08	0.3	1000/1000	1.1	1.3	0.02	0.081	0.077	0.081	0.079
G-GE	5.00E-08	0.3	1000/1000	1.1	1.4	0.02	0.303	0.286	0.301	0.289
G-GE	5.00E-08	0.3	1000/1000	1.1	1.5	0.02	0.601	0.577	0.601	0.601
G-GE	5.00E-08	0.3	1000/1000	1.2	1	0.02	0.003	0.002	0.003	0.004
G-GE	5.00E-08	0.3	1000/1000	1.2	1.1	0.02	0.026	0.021	0.026	0.028
G-GE	5.00E-08	0.3	1000/1000	1.2	1.2	0.02	0.135	0.129	0.135	0.132
G-GE	5.00E-08	0.3	1000/1000	1.2	1.3	0.02	0.432	0.414	0.433	0.422
G-GE	5.00E-08	0.3	1000/1000	1.2	1.4	0.02	0.737	0.715	0.736	0.733
G-GE	5.00E-08	0.3	1000/1000	1.2	1.5	0.02	0.911	0.887	0.911	0.911
G-GE	5.00E-08	0.3	5000/5000	1.1	1	0.02	0.017	0.015	0.017	0.018
G-GE	5.00E-08	0.3	5000/5000	1.1	1.1	0.02	0.35	0.325	0.35	0.352
G-GE	5.00E-08	0.3	5000/5000	1.1	1.2	0.02	0.953	0.948	0.953	0.947
G-GE	5.00E-08	0.3	5000/5000	1.1	1.3	0.02	1	1	1	1
G-GE	5.00E-08	0.3	5000/5000	1.1	1.4	0.02	1	1	1	1
G-GE	5.00E-08	0.3	5000/5000	1.1	1.5	0.02	1	1	1	1
G-GE	5.00E-08	0.3	5000/5000	1.2	1	0.02	0.74	0.724	0.74	0.729
G-GE	5.00E-08	0.3	5000/5000	1.2	1.1	0.02	0.994	0.993	0.994	0.993
G-GE	5.00E-08	0.3	5000/5000	1.2	1.2	0.02	1	1	1	1
G-GE	5.00E-08	0.3	5000/5000	1.2	1.3	0.02	1	1	1	1
G-GE	5.00E-08	0.3	5000/5000	1.2	1.4	0.02	1	1	1	1
G-GE	5.00E-08	0.3	5000/5000	1.2	1.5	0.02	1	1	1	1
G-GE	5.00E-08	0.1	1000/1000	1.1	1	0.05	0	0	0	0
G-GE	5.00E-08	0.1	1000/1000	1.1	1.1	0.05	0	0	0	0
G-GE	5.00E-08	0.1	1000/1000	1.1	1.2	0.05	0	0	0	0
G-GE	5.00E-08	0.1	1000/1000	1.1	1.3	0.05	0.004	0.004	0.004	0.004
G-GE	5.00E-08	0.1	1000/1000	1.1	1.4	0.05	0.015	0.018	0.015	0.018
G-GE	5.00E-08	0.1	1000/1000	1.1	1.5	0.05	0.051	0.051	0.051	0.051
G-GE	5.00E-08	0.1	1000/1000	1.2	1	0.05	0	0	0	0
G-GE	5.00E-08	0.1	1000/1000	1.2	1.1	0.05	0.005	0.004	0.005	0.004
G-GE	5.00E-08	0.1	1000/1000	1.2	1.2	0.05	0.006	0.007	0.006	0.007
G-GE	5.00E-08	0.1	1000/1000	1.2	1.3	0.05	0.021	0.02	0.021	0.02
G-GE	5.00E-08	0.1	1000/1000	1.2	1.4	0.05	0.07	0.061	0.07	0.061
G-GE	5.00E-08	0.1	1000/1000	1.2	1.5	0.05	0.176	0.17	0.177	0.17
G-GE	5.00E-08	0.1	5000/5000	1.1	1	0.05	0	0.001	0	0.001
G-GE	5.00E-08	0.1	5000/5000	1.1	1.1	0.05	0.014	0.016	0.014	0.016
G-GE	5.00E-08	0.1	5000/5000	1.1	1.2	0.05	0.201	0.186	0.202	0.186
G-GE	5.00E-08	0.1	5000/5000	1.1	1.3	0.05	0.704	0.694	0.704	0.694
G-GE	5.00E-08	0.1	5000/5000	1.1	1.4	0.05	0.956	0.954	0.956	0.954
G-GE	5.00E-08	0.1	5000/5000	1.1	1.5	0.05	0.998	0.998	0.998	0.998
G-GE	5.00E-08	0.1	5000/5000	1.2	1	0.05	0.064	0.06	0.064	0.06
G-GE	5.00E-08	0.1	5000/5000	1.2	1.1	0.05	0.412	0.389	0.412	0.389
G-GE	5.00E-08	0.1	5000/5000	1.2	1.2	0.05	0.846	0.836	0.846	0.836
G-GE	5.00E-08	0.1	5000/5000	1.2	1.3	0.05	0.993	0.991	0.993	0.991
G-GE	5.00E-08	0.1	5000/5000	1.2	1.4	0.05	1	1	1	1
G-GE	5.00E-08	0.1	5000/5000	1.2	1.5	0.05	1	1	1	1
G-GE	5.00E-08	0.3	1000/1000	1.1	1	0.05	0	0	0	0
G-GE	5.00E-08	0.3	1000/1000	1.1	1.1	0.05	0.001	0.001	0.001	0.001
G-GE	5.00E-08	0.3	1000/1000	1.1	1.2	0.05	0.019	0.013	0.019	0.015
G-GE	5.00E-08	0.3	1000/1000	1.1	1.3	0.05	0.07	0.063	0.071	0.07
G-GE	5.00E-08	0.3	1000/1000	1.1	1.4	0.05	0.265	0.242	0.266	0.254
G-GE	5.00E-08	0.3	1000/1000	1.1	1.5	0.05	0.571	0.501	0.571	0.564
G-GE	5.00E-08	0.3	1000/1000	1.2	1	0.05	0.001	0.001	0.001	0.002
G-GE	5.00E-08	0.3	1000/1000	1.2	1.1	0.05	0.029	0.017	0.029	0.03
G-GE	5.00E-08	0.3	1000/1000	1.2	1.2	0.05	0.131	0.111	0.131	0.124
G-GE	5.00E-08	0.3	1000/1000	1.2	1.3	0.05	0.407	0.354	0.406	0.396
G-GE	5.00E-08	0.3	1000/1000	1.2	1.4	0.05	0.714	0.651	0.714	0.711
G-GE	5.00E-08	0.3	1000/1000	1.2	1.5	0.05	0.889	0.855	0.889	0.886

Test	Alpha	MAF	#case/control	beta2	beta3	Missing rate	CST	PM1	PM2	median imputation
G-GE	5.00E-08	0.3	5000/5000	1.1	1	0.05	0.016	0.015	0.016	0.012
G-GE	5.00E-08	0.3	5000/5000	1.1	1.1	0.05	0.31	0.265	0.31	0.299
G-GE	5.00E-08	0.3	5000/5000	1.1	1.2	0.05	0.941	0.91	0.941	0.935
G-GE	5.00E-08	0.3	5000/5000	1.1	1.3	0.05	1	0.999	1	1
G-GE	5.00E-08	0.3	5000/5000	1.1	1.4	0.05	1	1	1	1
G-GE	5.00E-08	0.3	5000/5000	1.1	1.5	0.05	1	1	1	1
G-GE	5.00E-08	0.3	5000/5000	1.2	1	0.05	0.7	0.652	0.701	0.685
G-GE	5.00E-08	0.3	5000/5000	1.2	1.1	0.05	0.987	0.986	0.987	0.986
G-GE	5.00E-08	0.3	5000/5000	1.2	1.2	0.05	1	1	1	1
G-GE	5.00E-08	0.3	5000/5000	1.2	1.3	0.05	1	1	1	1
G-GE	5.00E-08	0.3	5000/5000	1.2	1.4	0.05	1	1	1	1
G-GE	5.00E-08	0.3	5000/5000	1.2	1.5	0.05	1	1	1	1
G-GE	5.00E-08	0.1	1000/1000	1.1	1	0.1	0	0	0	0
G-GE	5.00E-08	0.1	1000/1000	1.1	1.1	0.1	0	0	0	0
G-GE	5.00E-08	0.1	1000/1000	1.1	1.2	0.1	0	0	0	0
G-GE	5.00E-08	0.1	1000/1000	1.1	1.3	0.1	0.003	0.003	0.003	0.003
G-GE	5.00E-08	0.1	1000/1000	1.1	1.4	0.1	0.014	0.012	0.014	0.012
G-GE	5.00E-08	0.1	1000/1000	1.1	1.5	0.1	0.04	0.035	0.04	0.035
G-GE	5.00E-08	0.1	1000/1000	1.2	1	0.1	0	0	0	0
G-GE	5.00E-08	0.1	1000/1000	1.2	1.1	0.1	0.004	0.004	0.004	0.004
G-GE	5.00E-08	0.1	1000/1000	1.2	1.2	0.1	0.004	0.003	0.004	0.003
G-GE	5.00E-08	0.1	1000/1000	1.2	1.3	0.1	0.016	0.014	0.016	0.014
G-GE	5.00E-08	0.1	1000/1000	1.2	1.4	0.1	0.056	0.045	0.056	0.045
G-GE	5.00E-08	0.1	1000/1000	1.2	1.5	0.1	0.148	0.129	0.147	0.129
G-GE	5.00E-08	0.1	5000/5000	1.1	1	0.1	0	0	0	0
G-GE	5.00E-08	0.1	5000/5000	1.1	1.1	0.1	0.013	0.009	0.013	0.009
G-GE	5.00E-08	0.1	5000/5000	1.1	1.2	0.1	0.17	0.16	0.17	0.16
G-GE	5.00E-08	0.1	5000/5000	1.1	1.3	0.1	0.65	0.623	0.65	0.623
G-GE	5.00E-08	0.1	5000/5000	1.1	1.4	0.1	0.946	0.932	0.947	0.932
G-GE	5.00E-08	0.1	5000/5000	1.1	1.5	0.1	0.997	0.995	0.997	0.995
G-GE	5.00E-08	0.1	5000/5000	1.2	1	0.1	0.053	0.047	0.053	0.047
G-GE	5.00E-08	0.1	5000/5000	1.2	1.1	0.1	0.364	0.318	0.365	0.318
G-GE	5.00E-08	0.1	5000/5000	1.2	1.2	0.1	0.808	0.785	0.808	0.785
G-GE	5.00E-08	0.1	5000/5000	1.2	1.3	0.1	0.985	0.979	0.985	0.979
G-GE	5.00E-08	0.1	5000/5000	1.2	1.4	0.1	0.999	0.999	0.999	0.999
G-GE	5.00E-08	0.1	5000/5000	1.2	1.5	0.1	1	1	1	1
G-GE	5.00E-08	0.3	1000/1000	1.1	1	0.1	0	0	0	0
G-GE	5.00E-08	0.3	1000/1000	1.1	1.1	0.1	0	0	0	0
G-GE	5.00E-08	0.3	1000/1000	1.1	1.2	0.1	0.013	0.01	0.013	0.01
G-GE	5.00E-08	0.3	1000/1000	1.1	1.3	0.1	0.058	0.042	0.058	0.056
G-GE	5.00E-08	0.3	1000/1000	1.1	1.4	0.1	0.221	0.171	0.221	0.211
G-GE	5.00E-08	0.3	1000/1000	1.1	1.5	0.1	0.517	0.392	0.518	0.503
G-GE	5.00E-08	0.3	1000/1000	1.2	1	0.1	0.001	0.001	0.001	0.001
G-GE	5.00E-08	0.3	1000/1000	1.2	1.1	0.1	0.023	0.01	0.023	0.02
G-GE	5.00E-08	0.3	1000/1000	1.2	1.2	0.1	0.114	0.08	0.113	0.104
G-GE	5.00E-08	0.3	1000/1000	1.2	1.3	0.1	0.348	0.258	0.349	0.33
G-GE	5.00E-08	0.3	1000/1000	1.2	1.4	0.1	0.657	0.54	0.658	0.639
G-GE	5.00E-08	0.3	1000/1000	1.2	1.5	0.1	0.841	0.762	0.841	0.83
G-GE	5.00E-08	0.3	5000/5000	1.1	1	0.1	0.014	0.012	0.014	0.013
G-GE	5.00E-08	0.3	5000/5000	1.1	1.1	0.1	0.284	0.211	0.284	0.257
G-GE	5.00E-08	0.3	5000/5000	1.1	1.2	0.1	0.915	0.848	0.915	0.894
G-GE	5.00E-08	0.3	5000/5000	1.1	1.3	0.1	0.999	0.996	0.999	1
G-GE	5.00E-08	0.3	5000/5000	1.1	1.4	0.1	1	1	1	1
G-GE	5.00E-08	0.3	5000/5000	1.1	1.5	0.1	1	1	1	1
G-GE	5.00E-08	0.3	5000/5000	1.2	1	0.1	0.661	0.547	0.661	0.625
G-GE	5.00E-08	0.3	5000/5000	1.2	1.1	0.1	0.984	0.963	0.984	0.979
G-GE	5.00E-08	0.3	5000/5000	1.2	1.2	0.1	1	1	1	1
G-GE	5.00E-08	0.3	5000/5000	1.2	1.3	0.1	1	1	1	1
G-GE	5.00E-08	0.3	5000/5000	1.2	1.4	0.1	1	1	1	1
G-GE	5.00E-08	0.3	5000/5000	1.2	1.5	0.1	1	1	1	1

Test	Alpha	MAF	#case/control	beta2	beta3	Missing rate	CST	PM1	PM2	median imputation
G-GE	5.00E-08	0.1	1000/1000	1.1	1	0.3	0	0	0	0
G-GE	5.00E-08	0.1	1000/1000	1.1	1.1	0.3	0	0.001	0	0.001
G-GE	5.00E-08	0.1	1000/1000	1.1	1.2	0.3	0	0	0	0
G-GE	5.00E-08	0.1	1000/1000	1.1	1.3	0.3	0.001	0.001	0.001	0.001
G-GE	5.00E-08	0.1	1000/1000	1.1	1.4	0.3	0.005	0.002	0.005	0.002
G-GE	5.00E-08	0.1	1000/1000	1.1	1.5	0.3	0.009	0.008	0.009	0.008
G-GE	5.00E-08	0.1	1000/1000	1.2	1	0.3	0	0	0	0
G-GE	5.00E-08	0.1	1000/1000	1.2	1.1	0.3	0.002	0.001	0.002	0.001
G-GE	5.00E-08	0.1	1000/1000	1.2	1.2	0.3	0.001	0.001	0.001	0.001
G-GE	5.00E-08	0.1	1000/1000	1.2	1.3	0.3	0.005	0.003	0.005	0.003
G-GE	5.00E-08	0.1	1000/1000	1.2	1.4	0.3	0.015	0.008	0.015	0.008
G-GE	5.00E-08	0.1	1000/1000	1.2	1.5	0.3	0.061	0.036	0.06	0.036
G-GE	5.00E-08	0.1	5000/5000	1.1	1	0.3	0	0	0	0
G-GE	5.00E-08	0.1	5000/5000	1.1	1.1	0.3	0.005	0.003	0.006	0.003
G-GE	5.00E-08	0.1	5000/5000	1.1	1.2	0.3	0.081	0.064	0.081	0.064
G-GE	5.00E-08	0.1	5000/5000	1.1	1.3	0.3	0.383	0.304	0.383	0.304
G-GE	5.00E-08	0.1	5000/5000	1.1	1.4	0.3	0.785	0.698	0.785	0.698
G-GE	5.00E-08	0.1	5000/5000	1.1	1.5	0.3	0.962	0.929	0.962	0.929
G-GE	5.00E-08	0.1	5000/5000	1.2	1	0.3	0.027	0.026	0.027	0.026
G-GE	5.00E-08	0.1	5000/5000	1.2	1.1	0.3	0.173	0.125	0.174	0.125
G-GE	5.00E-08	0.1	5000/5000	1.2	1.2	0.3	0.54	0.448	0.54	0.448
G-GE	5.00E-08	0.1	5000/5000	1.2	1.3	0.3	0.893	0.83	0.893	0.83
G-GE	5.00E-08	0.1	5000/5000	1.2	1.4	0.3	0.986	0.975	0.986	0.975
G-GE	5.00E-08	0.1	5000/5000	1.2	1.5	0.3	1	0.995	1	0.995
G-GE	5.00E-08	0.3	1000/1000	1.1	1	0.3	0	0	0	0
G-GE	5.00E-08	0.3	1000/1000	1.1	1.1	0.3	0.001	0	0.001	0
G-GE	5.00E-08	0.3	1000/1000	1.1	1.2	0.3	0.005	0	0.005	0.003
G-GE	5.00E-08	0.3	1000/1000	1.1	1.3	0.3	0.021	0.007	0.021	0.017
G-GE	5.00E-08	0.3	1000/1000	1.1	1.4	0.3	0.102	0.036	0.1	0.078
G-GE	5.00E-08	0.3	1000/1000	1.1	1.5	0.3	0.272	0.11	0.272	0.225
G-GE	5.00E-08	0.3	1000/1000	1.2	1	0.3	0	0	0	0
G-GE	5.00E-08	0.3	1000/1000	1.2	1.1	0.3	0.006	0.001	0.006	0.005
G-GE	5.00E-08	0.3	1000/1000	1.2	1.2	0.3	0.051	0.013	0.051	0.039
G-GE	5.00E-08	0.3	1000/1000	1.2	1.3	0.3	0.161	0.061	0.162	0.143
G-GE	5.00E-08	0.3	1000/1000	1.2	1.4	0.3	0.378	0.158	0.377	0.335
G-GE	5.00E-08	0.3	1000/1000	1.2	1.5	0.3	0.622	0.325	0.621	0.575
G-GE	5.00E-08	0.3	5000/5000	1.1	1	0.3	0.006	0.001	0.006	0.002
G-GE	5.00E-08	0.3	5000/5000	1.1	1.1	0.3	0.129	0.056	0.13	0.094
G-GE	5.00E-08	0.3	5000/5000	1.1	1.2	0.3	0.702	0.438	0.702	0.626
G-GE	5.00E-08	0.3	5000/5000	1.1	1.3	0.3	0.978	0.886	0.978	0.968
G-GE	5.00E-08	0.3	5000/5000	1.1	1.4	0.3	1	0.994	1	0.998
G-GE	5.00E-08	0.3	5000/5000	1.1	1.5	0.3	1	1	1	1
G-GE	5.00E-08	0.3	5000/5000	1.2	1	0.3	0.374	0.194	0.374	0.302
G-GE	5.00E-08	0.3	5000/5000	1.2	1.1	0.3	0.893	0.663	0.893	0.839
G-GE	5.00E-08	0.3	5000/5000	1.2	1.2	0.3	0.995	0.959	0.995	0.991
G-GE	5.00E-08	0.3	5000/5000	1.2	1.3	0.3	1	0.999	1	1
G-GE	5.00E-08	0.3	5000/5000	1.2	1.4	0.3	1	1	1	1
G-GE	5.00E-08	0.3	5000/5000	1.2	1.5	0.3	1	1	1	1
