# Supplementary material for: Fast score test with global null estimation regardless of missing genotypes
Source: PLoS One. 2018 Jul 5;13(7):e0199692. doi: 10.1371/journal.pone.0199692 (PMC6033421; doi:10.1371/journal.pone.0199692)
Supplement: S3 Table — G test Power of the conventional score test (CST), the proposed method 1 (PM1), and the proposed method 2 (PM2) under missing rate (2%, 5%, 10%, 30%), minor allele frequency (MAF) (10%, 30%), and the number of case/control (1,000, 5,000). The x-axis denotes genetic odds ratios (ORg = exp(βg)). The significance level is 5 × 10^−8^. G-GE test Power of CST, PM1, and PM2 under genetic odds ratios (ORg = exp(βg) = 1.1, 1.2), missing rate (2%, 5%, 10%, 30%), minor allele frequency (MAF) (10%, 30%), and the number of case/control is 1,000. The x-axis denotes gene-environment interaction odds ratios (ORge = exp(βge)). The significance level is 5 × 10^−8^. (PDF) [file pone.0199692.s013.pdf]

Test	Alpha	MAF	#case/control	beta2	beta3	Missing rate	CST	PM1	PM2	median imputation
G	5.00E-08	0.1	100/100	1	-	0.02	0	0	0	0
G	5.00E-08	0.1	100/100	2	-	0.02	0.001	0.001	0.001	0.001
G	5.00E-08	0.1	100/100	3	-	0.02	0.036	0.033	0.036	0.033
G	5.00E-08	0.1	100/100	4	-	0.02	0.247	0.246	0.247	0.246
G	5.00E-08	0.1	100/100	5	-	0.02	0.628	0.619	0.629	0.619
G	5.00E-08	0.1	500/500	1	-	0.02	0	0	0	0
G	5.00E-08	0.1	500/500	2	-	0.02	0.379	0.365	0.378	0.365
G	5.00E-08	0.1	500/500	3	-	0.02	1	1	1	1
G	5.00E-08	0.1	500/500	4	-	0.02	1	1	1	1
G	5.00E-08	0.1	500/500	5	-	0.02	1	1	1	1
G	5.00E-08	0.3	100/100	1	-	0.02	0	0	0	0
G	5.00E-08	0.3	100/100	2	-	0.02	0.012	0.009	0.012	0.012
G	5.00E-08	0.3	100/100	3	-	0.02	0.334	0.305	0.335	0.334
G	5.00E-08	0.3	100/100	4	-	0.02	0.843	0.798	0.841	0.843
G	5.00E-08	0.3	100/100	5	-	0.02	0.974	0.968	0.974	0.974
G	5.00E-08	0.3	500/500	1	-	0.02	0	0	0	0
G	5.00E-08	0.3	500/500	2	-	0.02	0.975	0.971	0.975	0.972
G	5.00E-08	0.3	500/500	3	-	0.02	1	1	1	1
G	5.00E-08	0.3	500/500	4	-	0.02	1	1	1	1
G	5.00E-08	0.3	500/500	5	-	0.02	1	1	1	1
G	5.00E-08	0.1	100/100	1	-	0.05	0	0	0	0
G	5.00E-08	0.1	100/100	2	-	0.05	0.001	0.001	0.001	0.001
G	5.00E-08	0.1	100/100	3	-	0.05	0.031	0.027	0.031	0.027
G	5.00E-08	0.1	100/100	4	-	0.05	0.222	0.21	0.22	0.21
G	5.00E-08	0.1	100/100	5	-	0.05	0.584	0.56	0.582	0.56
G	5.00E-08	0.1	500/500	1	-	0.05	0	0	0	0
G	5.00E-08	0.1	500/500	2	-	0.05	0.349	0.338	0.349	0.338
G	5.00E-08	0.1	500/500	3	-	0.05	0.999	0.999	0.999	0.999
G	5.00E-08	0.1	500/500	4	-	0.05	1	1	1	1
G	5.00E-08	0.1	500/500	5	-	0.05	1	1	1	1
G	5.00E-08	0.3	100/100	1	-	0.05	0	0	0	0
G	5.00E-08	0.3	100/100	2	-	0.05	0.01	0.01	0.01	0.01
G	5.00E-08	0.3	100/100	3	-	0.05	0.298	0.228	0.299	0.295
G	5.00E-08	0.3	100/100	4	-	0.05	0.812	0.714	0.812	0.815
G	5.00E-08	0.3	100/100	5	-	0.05	0.972	0.928	0.972	0.974
G	5.00E-08	0.3	500/500	1	-	0.05	0	0	0	0
G	5.00E-08	0.3	500/500	2	-	0.05	0.963	0.945	0.963	0.962
G	5.00E-08	0.3	500/500	3	-	0.05	1	1	1	1
G	5.00E-08	0.3	500/500	4	-	0.05	1	1	1	1
G	5.00E-08	0.3	500/500	5	-	0.05	1	1	1	1
G	5.00E-08	0.1	100/100	1	-	0.1	0	0	0	0
G	5.00E-08	0.1	100/100	2	-	0.1	0.001	0.001	0.001	0.001
G	5.00E-08	0.1	100/100	3	-	0.1	0.031	0.023	0.031	0.023
G	5.00E-08	0.1	100/100	4	-	0.1	0.181	0.143	0.18	0.143
G	5.00E-08	0.1	100/100	5	-	0.1	0.516	0.453	0.512	0.453
G	5.00E-08	0.1	500/500	1	-	0.1	0	0	0	0
G	5.00E-08	0.1	500/500	2	-	0.1	0.301	0.264	0.301	0.264
G	5.00E-08	0.1	500/500	3	-	0.1	0.999	0.996	0.999	0.996
G	5.00E-08	0.1	500/500	4	-	0.1	1	1	1	1
G	5.00E-08	0.1	500/500	5	-	0.1	1	1	1	1
G	5.00E-08	0.3	100/100	1	-	0.1	0	0	0	0
G	5.00E-08	0.3	100/100	2	-	0.1	0.005	0.005	0.005	0.004
G	5.00E-08	0.3	100/100	3	-	0.1	0.231	0.153	0.23	0.236
G	5.00E-08	0.3	100/100	4	-	0.1	0.764	0.557	0.758	0.762
G	5.00E-08	0.3	100/100	5	-	0.1	0.959	0.819	0.957	0.957
G	5.00E-08	0.3	500/500	1	-	0.1	0	0	0	0
G	5.00E-08	0.3	500/500	2	-	0.1	0.942	0.878	0.941	0.933
G	5.00E-08	0.3	500/500	3	-	0.1	1	1	1	1
G	5.00E-08	0.3	500/500	4	-	0.1	1	1	1	1
G	5.00E-08	0.3	500/500	5	-	0.1	1	1	1	1
G	5.00E-08	0.1	100/100	1	-	0.3	0	0	0	0
G	5.00E-08	0.1	100/100	2	-	0.3	0.001	0.001	0.001	0.001
G	5.00E-08	0.1	100/100	3	-	0.3	0.004	0	0.004	0
G	5.00E-08	0.1	100/100	4	-	0.3	0.048	0.019	0.045	0.019
G	5.00E-08	0.1	100/100	5	-	0.3	0.227	0.117	0.225	0.116

Test	Alpha	MAF	#case/control	beta2	beta3	Missing rate	CST	PM1	PM2	median imputation
G	5.00E-08	0.1	500/500	1	-	0.3	0	0	0	0
G	5.00E-08	0.1	500/500	2	-	0.3	0.116	0.075	0.115	0.075
G	5.00E-08	0.1	500/500	3	-	0.3	0.975	0.933	0.975	0.933
G	5.00E-08	0.1	500/500	4	-	0.3	1	1	1	1
G	5.00E-08	0.1	500/500	5	-	0.3	1	1	1	1
G	5.00E-08	0.3	100/100	1	-	0.3	0	0	0	0
G	5.00E-08	0.3	100/100	2	-	0.3	0.002	0.001	0.002	0.001
G	5.00E-08	0.3	100/100	3	-	0.3	0.093	0.022	0.089	0.085
G	5.00E-08	0.3	100/100	4	-	0.3	0.428	0.101	0.42	0.428
G	5.00E-08	0.3	100/100	5	-	0.3	0.755	0.253	0.748	0.755
G	5.00E-08	0.3	500/500	1	-	0.3	0	0	0	0
G	5.00E-08	0.3	500/500	2	-	0.3	0.783	0.432	0.782	0.746
G	5.00E-08	0.3	500/500	3	-	0.3	1	0.993	1	1
G	5.00E-08	0.3	500/500	4	-	0.3	1	1	1	1
G	5.00E-08	0.3	500/500	5	-	0.3	1	1	1	1

Test	Alpha	MAF	#case/control	beta2	beta3	Missing rate	CST	PM1	PM2	median imputation
G	5.00E-08	0.1	1000/2000	1	-	0.02	0	0	0	0
G	5.00E-08	0.1	1000/2000	1.2	-	0.02	0	0.001	0	0.001
G	5.00E-08	0.1	1000/2000	1.4	-	0.02	0.079	0.08	0.079	0.08
G	5.00E-08	0.1	1000/2000	1.6	-	0.02	0.598	0.598	0.599	0.598
G	5.00E-08	0.1	1000/2000	1.8	-	0.02	0.966	0.964	0.966	0.964
G	5.00E-08	0.1	1000/2000	2	-	0.02	0.999	0.999	0.999	0.999
G	5.00E-08	0.3	1000/2000	1	-	0.02	0	0	0	0
G	5.00E-08	0.3	1000/2000	1.2	-	0.02	0.011	0.01	0.011	0.01
G	5.00E-08	0.3	1000/2000	1.4	-	0.02	0.615	0.593	0.615	0.616
G	5.00E-08	0.3	1000/2000	1.6	-	0.02	1	0.999	1	1
G	5.00E-08	0.3	1000/2000	1.8	-	0.02	1	1	1	1
G	5.00E-08	0.3	1000/2000	2	-	0.02	1	1	1	1
G	5.00E-08	0.1	1000/2000	1	-	0.05	0	0	0	0
G	5.00E-08	0.1	1000/2000	1.2	-	0.05	0	0.001	0	0.001
G	5.00E-08	0.1	1000/2000	1.4	-	0.05	0.068	0.064	0.068	0.064
G	5.00E-08	0.1	1000/2000	1.6	-	0.05	0.567	0.554	0.569	0.554
G	5.00E-08	0.1	1000/2000	1.8	-	0.05	0.951	0.948	0.953	0.948
G	5.00E-08	0.1	1000/2000	2	-	0.05	0.997	0.998	0.998	0.998
G	5.00E-08	0.3	1000/2000	1	-	0.05	0	0	0	0
G	5.00E-08	0.3	1000/2000	1.2	-	0.05	0.009	0.006	0.009	0.008
G	5.00E-08	0.3	1000/2000	1.4	-	0.05	0.593	0.537	0.594	0.574
G	5.00E-08	0.3	1000/2000	1.6	-	0.05	1	0.992	1	0.999
G	5.00E-08	0.3	1000/2000	1.8	-	0.05	1	1	1	1
G	5.00E-08	0.3	1000/2000	2	-	0.05	1	1	1	1
G	5.00E-08	0.1	1000/2000	1	-	0.1	0	0	0	0
G	5.00E-08	0.1	1000/2000	1.2	-	0.1	0	0	0	0
G	5.00E-08	0.1	1000/2000	1.4	-	0.1	0.056	0.053	0.057	0.053
G	5.00E-08	0.1	1000/2000	1.6	-	0.1	0.508	0.482	0.509	0.482
G	5.00E-08	0.1	1000/2000	1.8	-	0.1	0.931	0.91	0.931	0.91
G	5.00E-08	0.1	1000/2000	2	-	0.1	0.996	0.996	0.997	0.996
G	5.00E-08	0.3	1000/2000	1	-	0.1	0	0	0	0
G	5.00E-08	0.3	1000/2000	1.2	-	0.1	0.006	0.005	0.006	0.003
G	5.00E-08	0.3	1000/2000	1.4	-	0.1	0.533	0.42	0.532	0.504
G	5.00E-08	0.3	1000/2000	1.6	-	0.1	0.99	0.973	0.99	0.99
G	5.00E-08	0.3	1000/2000	1.8	-	0.1	1	1	1	1
G	5.00E-08	0.3	1000/2000	2	-	0.1	1	1	1	1
G	5.00E-08	0.1	1000/2000	1	-	0.3	0	0	0	0
G	5.00E-08	0.1	1000/2000	1.2	-	0.3	0.001	0	0.001	0
G	5.00E-08	0.1	1000/2000	1.4	-	0.3	0.024	0.012	0.024	0.012
G	5.00E-08	0.1	1000/2000	1.6	-	0.3	0.285	0.223	0.283	0.223
G	5.00E-08	0.1	1000/2000	1.8	-	0.3	0.73	0.652	0.733	0.652
G	5.00E-08	0.1	1000/2000	2	-	0.3	0.966	0.946	0.966	0.946
G	5.00E-08	0.3	1000/2000	1	-	0.3	0	0	0	0
G	5.00E-08	0.3	1000/2000	1.2	-	0.3	0.001	0	0.001	0.002
G	5.00E-08	0.3	1000/2000	1.4	-	0.3	0.28	0.145	0.281	0.233
G	5.00E-08	0.3	1000/2000	1.6	-	0.3	0.92	0.726	0.92	0.888
G	5.00E-08	0.3	1000/2000	1.8	-	0.3	0.999	0.973	0.999	0.998
G	5.00E-08	0.3	1000/2000	2	-	0.3	1	1	1	1

Test	Alpha	MAF	#case/control	beta2	beta3	Missing rate	CST	PM1	PM2	median imputation
G-GE	5.00E-08	0.1	100/100	1.1	1	0.02	0	0	0	0
G-GE	5.00E-08	0.1	100/100	1.1	1.5	0.02	0	0	0	0
G-GE	5.00E-08	0.1	100/100	1.1	2	0.02	0	0	0	0
G-GE	5.00E-08	0.1	100/100	1.1	2.5	0.02	0	0.001	0	0.001
G-GE	5.00E-08	0.1	100/100	1.1	3	0.02	0.002	0.001	0.002	0.001
G-GE	5.00E-08	0.1	100/100	1.2	1	0.02	0	0	0	0
G-GE	5.00E-08	0.1	100/100	1.2	1.5	0.02	0	0	0	0
G-GE	5.00E-08	0.1	100/100	1.2	2	0.02	0	0	0	0
G-GE	5.00E-08	0.1	100/100	1.2	2.5	0.02	0.002	0.002	0.002	0.002
G-GE	5.00E-08	0.1	100/100	1.2	3	0.02	0.005	0.005	0.005	0.005
G-GE	5.00E-08	0.1	500/500	1.1	1	0.02	0	0	0	0
G-GE	5.00E-08	0.1	500/500	1.1	1.5	0.02	0.002	0.002	0.002	0.002
G-GE	5.00E-08	0.1	500/500	1.1	2	0.02	0.146	0.138	0.147	0.138
G-GE	5.00E-08	0.1	500/500	1.1	2.5	0.02	0.646	0.64	0.649	0.64
G-GE	5.00E-08	0.1	500/500	1.1	3	0.02	0.947	0.94	0.947	0.94
G-GE	5.00E-08	0.1	500/500	1.2	1	0.02	0	0	0	0
G-GE	5.00E-08	0.1	500/500	1.2	1.5	0.02	0.007	0.01	0.007	0.01
G-GE	5.00E-08	0.1	500/500	1.2	2	0.02	0.317	0.311	0.318	0.311
G-GE	5.00E-08	0.1	500/500	1.2	2.5	0.02	0.836	0.828	0.836	0.828
G-GE	5.00E-08	0.1	500/500	1.2	3	0.02	0.986	0.989	0.986	0.989
G-GE	5.00E-08	0.3	100/100	1.1	1	0.02	0	0	0	0
G-GE	5.00E-08	0.3	100/100	1.1	1.5	0.02	0	0.001	0	0
G-GE	5.00E-08	0.3	100/100	1.1	2	0.02	0.003	0.002	0.003	0.003
G-GE	5.00E-08	0.3	100/100	1.1	2.5	0.02	0.032	0.03	0.032	0.032
G-GE	5.00E-08	0.3	100/100	1.1	3	0.02	0.109	0.095	0.11	0.109
G-GE	5.00E-08	0.3	100/100	1.2	1	0.02	0	0	0	0
G-GE	5.00E-08	0.3	100/100	1.2	1.5	0.02	0	0	0	0
G-GE	5.00E-08	0.3	100/100	1.2	2	0.02	0.003	0.002	0.003	0.003
G-GE	5.00E-08	0.3	100/100	1.2	2.5	0.02	0.047	0.043	0.047	0.047
G-GE	5.00E-08	0.3	100/100	1.2	3	0.02	0.183	0.162	0.181	0.18
G-GE	5.00E-08	0.3	500/500	1.1	1	0.02	0	0	0	0
G-GE	5.00E-08	0.3	500/500	1.1	1.5	0.02	0.071	0.065	0.071	0.072
G-GE	5.00E-08	0.3	500/500	1.1	2	0.02	0.825	0.81	0.828	0.824
G-GE	5.00E-08	0.3	500/500	1.1	2.5	0.02	0.999	0.999	0.999	0.999
G-GE	5.00E-08	0.3	500/500	1.1	3	0.02	1	1	1	1
G-GE	5.00E-08	0.3	500/500	1.2	1	0.02	0	0	0	0
G-GE	5.00E-08	0.3	500/500	1.2	1.5	0.02	0.267	0.25	0.267	0.269
G-GE	5.00E-08	0.3	500/500	1.2	2	0.02	0.974	0.962	0.975	0.974
G-GE	5.00E-08	0.3	500/500	1.2	2.5	0.02	1	0.999	1	1
G-GE	5.00E-08	0.3	500/500	1.2	3	0.02	1	1	1	1
G-GE	5.00E-08	0.1	100/100	1.1	1	0.05	0	0	0	0
G-GE	5.00E-08	0.1	100/100	1.1	1.5	0.05	0	0	0	0
G-GE	5.00E-08	0.1	100/100	1.1	2	0.05	0	0	0	0
G-GE	5.00E-08	0.1	100/100	1.1	2.5	0.05	0	0	0	0
G-GE	5.00E-08	0.1	100/100	1.1	3	0.05	0.002	0	0.002	0
G-GE	5.00E-08	0.1	100/100	1.2	1	0.05	0	0	0	0
G-GE	5.00E-08	0.1	100/100	1.2	1.5	0.05	0	0	0	0
G-GE	5.00E-08	0.1	100/100	1.2	2	0.05	0	0	0	0
G-GE	5.00E-08	0.1	100/100	1.2	2.5	0.05	0	0	0	0
G-GE	5.00E-08	0.1	100/100	1.2	3	0.05	0.002	0.003	0.002	0.003
G-GE	5.00E-08	0.1	500/500	1.1	1	0.05	0	0	0	0
G-GE	5.00E-08	0.1	500/500	1.1	1.5	0.05	0.002	0.002	0.002	0.002
G-GE	5.00E-08	0.1	500/500	1.1	2	0.05	0.13	0.121	0.13	0.121
G-GE	5.00E-08	0.1	500/500	1.1	2.5	0.05	0.616	0.593	0.615	0.593
G-GE	5.00E-08	0.1	500/500	1.1	3	0.05	0.927	0.914	0.926	0.914
G-GE	5.00E-08	0.1	500/500	1.2	1	0.05	0	0	0	0
G-GE	5.00E-08	0.1	500/500	1.2	1.5	0.05	0.007	0.006	0.007	0.006
G-GE	5.00E-08	0.1	500/500	1.2	2	0.05	0.293	0.278	0.294	0.278
G-GE	5.00E-08	0.1	500/500	1.2	2.5	0.05	0.813	0.798	0.814	0.798
G-GE	5.00E-08	0.1	500/500	1.2	3	0.05	0.979	0.98	0.979	0.98

Test	Alpha	MAF	#case/control	beta2	beta3	Missing rate	CST	PM1	PM2	median imputation
G-GE	5.00E-08	0.3	100/100	1.1	1	0.05	0	0	0	0
G-GE	5.00E-08	0.3	100/100	1.1	1.5	0.05	0	0	0	0
G-GE	5.00E-08	0.3	100/100	1.1	2	0.05	0.001	0	0.001	0.002
G-GE	5.00E-08	0.3	100/100	1.1	2.5	0.05	0.027	0.022	0.026	0.026
G-GE	5.00E-08	0.3	100/100	1.1	3	0.05	0.099	0.076	0.099	0.097
G-GE	5.00E-08	0.3	100/100	1.2	1	0.05	0	0	0	0
G-GE	5.00E-08	0.3	100/100	1.2	1.5	0.05	0	0	0	0
G-GE	5.00E-08	0.3	100/100	1.2	2	0.05	0.002	0.003	0.002	0.001
G-GE	5.00E-08	0.3	100/100	1.2	2.5	0.05	0.049	0.031	0.049	0.046
G-GE	5.00E-08	0.3	100/100	1.2	3	0.05	0.169	0.117	0.167	0.167
G-GE	5.00E-08	0.3	500/500	1.1	1	0.05	0	0	0	0
G-GE	5.00E-08	0.3	500/500	1.1	1.5	0.05	0.063	0.052	0.063	0.061
G-GE	5.00E-08	0.3	500/500	1.1	2	0.05	0.8	0.749	0.801	0.794
G-GE	5.00E-08	0.3	500/500	1.1	2.5	0.05	0.999	0.994	0.998	0.998
G-GE	5.00E-08	0.3	500/500	1.1	3	0.05	1	1	1	1
G-GE	5.00E-08	0.3	500/500	1.2	1	0.05	0	0	0	0
G-GE	5.00E-08	0.3	500/500	1.2	1.5	0.05	0.243	0.195	0.244	0.243
G-GE	5.00E-08	0.3	500/500	1.2	2	0.05	0.969	0.942	0.968	0.967
G-GE	5.00E-08	0.3	500/500	1.2	2.5	0.05	1	0.998	1	1
G-GE	5.00E-08	0.3	500/500	1.2	3	0.05	1	1	1	1
G-GE	5.00E-08	0.1	100/100	1.1	1	0.1	0	0	0	0
G-GE	5.00E-08	0.1	100/100	1.1	1.5	0.1	0	0	0	0
G-GE	5.00E-08	0.1	100/100	1.1	2	0.1	0	0	0	0
G-GE	5.00E-08	0.1	100/100	1.1	2.5	0.1	0	0	0	0
G-GE	5.00E-08	0.1	100/100	1.1	3	0.1	0	0	0	0
G-GE	5.00E-08	0.1	100/100	1.2	1	0.1	0	0	0	0
G-GE	5.00E-08	0.1	100/100	1.2	1.5	0.1	0	0	0	0
G-GE	5.00E-08	0.1	100/100	1.2	2	0.1	0	0	0	0
G-GE	5.00E-08	0.1	100/100	1.2	2.5	0.1	0	0	0	0
G-GE	5.00E-08	0.1	100/100	1.2	3	0.1	0.001	0.001	0.002	0.001
G-GE	5.00E-08	0.1	500/500	1.1	1	0.1	0	0	0	0
G-GE	5.00E-08	0.1	500/500	1.1	1.5	0.1	0.002	0.003	0.002	0.003
G-GE	5.00E-08	0.1	500/500	1.1	2	0.1	0.102	0.088	0.102	0.088
G-GE	5.00E-08	0.1	500/500	1.1	2.5	0.1	0.541	0.504	0.539	0.504
G-GE	5.00E-08	0.1	500/500	1.1	3	0.1	0.891	0.857	0.891	0.857
G-GE	5.00E-08	0.1	500/500	1.2	1	0.1	0	0	0	0
G-GE	5.00E-08	0.1	500/500	1.2	1.5	0.1	0.005	0.006	0.005	0.006
G-GE	5.00E-08	0.1	500/500	1.2	2	0.1	0.24	0.216	0.242	0.216
G-GE	5.00E-08	0.1	500/500	1.2	2.5	0.1	0.752	0.713	0.752	0.713
G-GE	5.00E-08	0.1	500/500	1.2	3	0.1	0.975	0.963	0.975	0.963
G-GE	5.00E-08	0.3	100/100	1.1	1	0.1	0	0	0	0
G-GE	5.00E-08	0.3	100/100	1.1	1.5	0.1	0.001	0.001	0.001	0.001
G-GE	5.00E-08	0.3	100/100	1.1	2	0.1	0.002	0.001	0.002	0.001
G-GE	5.00E-08	0.3	100/100	1.1	2.5	0.1	0.022	0.012	0.022	0.021
G-GE	5.00E-08	0.3	100/100	1.1	3	0.1	0.072	0.039	0.073	0.074
G-GE	5.00E-08	0.3	100/100	1.2	1	0.1	0	0	0	0
G-GE	5.00E-08	0.3	100/100	1.2	1.5	0.1	0	0	0	0
G-GE	5.00E-08	0.3	100/100	1.2	2	0.1	0.001	0.001	0.001	0
G-GE	5.00E-08	0.3	100/100	1.2	2.5	0.1	0.038	0.016	0.041	0.043
G-GE	5.00E-08	0.3	100/100	1.2	3	0.1	0.135	0.062	0.13	0.13
G-GE	5.00E-08	0.3	500/500	1.1	1	0.1	0	0	0	0
G-GE	5.00E-08	0.3	500/500	1.1	1.5	0.1	0.051	0.037	0.052	0.044
G-GE	5.00E-08	0.3	500/500	1.1	2	0.1	0.75	0.601	0.749	0.744
G-GE	5.00E-08	0.3	500/500	1.1	2.5	0.1	0.995	0.979	0.995	0.996
G-GE	5.00E-08	0.3	500/500	1.1	3	0.1	1	1	1	1
G-GE	5.00E-08	0.3	500/500	1.2	1	0.1	0	0	0	0
G-GE	5.00E-08	0.3	500/500	1.2	1.5	0.1	0.199	0.13	0.199	0.187
G-GE	5.00E-08	0.3	500/500	1.2	2	0.1	0.944	0.883	0.944	0.941
G-GE	5.00E-08	0.3	500/500	1.2	2.5	0.1	0.999	0.991	0.999	0.999
G-GE	5.00E-08	0.3	500/500	1.2	3	0.1	1	1	1	1

Test	Alpha	MAF	#case/control	beta2	beta3	Missing rate	CST	PM1	PM2	median imputation
G-GE	5.00E-08	0.1	100/100	1.1	1	0.3	0	0	0	0
G-GE	5.00E-08	0.1	100/100	1.1	1.5	0.3	0	0	0	0
G-GE	5.00E-08	0.1	100/100	1.1	2	0.3	0	0	0	0
G-GE	5.00E-08	0.1	100/100	1.1	2.5	0.3	0	0	0	0
G-GE	5.00E-08	0.1	100/100	1.1	3	0.3	0	0	0	0
G-GE	5.00E-08	0.1	100/100	1.2	1	0.3	0	0	0	0
G-GE	5.00E-08	0.1	100/100	1.2	1.5	0.3	0	0	0	0
G-GE	5.00E-08	0.1	100/100	1.2	2	0.3	0	0	0	0
G-GE	5.00E-08	0.1	100/100	1.2	2.5	0.3	0	0	0	0
G-GE	5.00E-08	0.1	100/100	1.2	3	0.3	0	0	0	0
G-GE	5.00E-08	0.1	500/500	1.1	1	0.3	0	0	0	0
G-GE	5.00E-08	0.1	500/500	1.1	1.5	0.3	0	0	0	0
G-GE	5.00E-08	0.1	500/500	1.1	2	0.3	0.038	0.021	0.036	0.021
G-GE	5.00E-08	0.1	500/500	1.1	2.5	0.3	0.276	0.175	0.278	0.175
G-GE	5.00E-08	0.1	500/500	1.1	3	0.3	0.655	0.504	0.653	0.504
G-GE	5.00E-08	0.1	500/500	1.2	1	0.3	0	0	0	0
G-GE	5.00E-08	0.1	500/500	1.2	1.5	0.3	0.001	0	0.001	0
G-GE	5.00E-08	0.1	500/500	1.2	2	0.3	0.093	0.06	0.093	0.06
G-GE	5.00E-08	0.1	500/500	1.2	2.5	0.3	0.467	0.345	0.463	0.345
G-GE	5.00E-08	0.1	500/500	1.2	3	0.3	0.86	0.73	0.856	0.73
G-GE	5.00E-08	0.3	100/100	1.1	1	0.3	0	0	0	0
G-GE	5.00E-08	0.3	100/100	1.1	1.5	0.3	0	0	0	0
G-GE	5.00E-08	0.3	100/100	1.1	2	0.3	0	0	0	0
G-GE	5.00E-08	0.3	100/100	1.1	2.5	0.3	0.004	0	0.003	0.003
G-GE	5.00E-08	0.3	100/100	1.1	3	0.3	0.02	0.003	0.02	0.02
G-GE	5.00E-08	0.3	100/100	1.2	1	0.3	0	0	0	0
G-GE	5.00E-08	0.3	100/100	1.2	1.5	0.3	0	0	0	0
G-GE	5.00E-08	0.3	100/100	1.2	2	0.3	0	0.001	0	0
G-GE	5.00E-08	0.3	100/100	1.2	2.5	0.3	0.01	0	0.012	0.012
G-GE	5.00E-08	0.3	100/100	1.2	3	0.3	0.041	0.003	0.04	0.037
G-GE	5.00E-08	0.3	500/500	1.1	1	0.3	0	0	0	0
G-GE	5.00E-08	0.3	500/500	1.1	1.5	0.3	0.02	0.009	0.018	0.01
G-GE	5.00E-08	0.3	500/500	1.1	2	0.3	0.469	0.177	0.465	0.44
G-GE	5.00E-08	0.3	500/500	1.1	2.5	0.3	0.938	0.631	0.936	0.928
G-GE	5.00E-08	0.3	500/500	1.1	3	0.3	0.997	0.908	0.997	0.996
G-GE	5.00E-08	0.3	500/500	1.2	1	0.3	0	0	0	0
G-GE	5.00E-08	0.3	500/500	1.2	1.5	0.3	0.074	0.017	0.073	0.063
G-GE	5.00E-08	0.3	500/500	1.2	2	0.3	0.776	0.342	0.772	0.738
G-GE	5.00E-08	0.3	500/500	1.2	2.5	0.3	0.989	0.795	0.989	0.987
G-GE	5.00E-08	0.3	500/500	1.2	3	0.3	1	0.97	1	1

Test	Alpha	MAF	#case/control	beta2	beta3	Missing rate	CST	PM1	PM2	median imputation
G-GE	5.00E-08	0.1	1000/2000	1.1	1	0.02	0	0	0	0
G-GE	5.00E-08	0.1	1000/2000	1.1	1.1	0.02	0	0	0	0
G-GE	5.00E-08	0.1	1000/2000	1.1	1.2	0.02	0.003	0.004	0.003	0.004
G-GE	5.00E-08	0.1	1000/2000	1.1	1.3	0.02	0.024	0.024	0.024	0.024
G-GE	5.00E-08	0.1	1000/2000	1.1	1.4	0.02	0.08	0.08	0.079	0.08
G-GE	5.00E-08	0.1	1000/2000	1.1	1.5	0.02	0.212	0.215	0.212	0.215
G-GE	5.00E-08	0.1	1000/2000	1.2	1	0.02	0.001	0.001	0.001	0.001
G-GE	5.00E-08	0.1	1000/2000	1.2	1.1	0.02	0.008	0.009	0.009	0.009
G-GE	5.00E-08	0.1	1000/2000	1.2	1.2	0.02	0.027	0.025	0.028	0.025
G-GE	5.00E-08	0.1	1000/2000	1.2	1.3	0.02	0.114	0.121	0.115	0.121
G-GE	5.00E-08	0.1	1000/2000	1.2	1.4	0.02	0.298	0.293	0.297	0.293
G-GE	5.00E-08	0.1	1000/2000	1.2	1.5	0.02	0.584	0.586	0.584	0.586
G-GE	5.00E-08	0.3	1000/2000	1.1	1	0.02	0	0	0	0
G-GE	5.00E-08	0.3	1000/2000	1.1	1.1	0.02	0.002	0.002	0.002	0.002
G-GE	5.00E-08	0.3	1000/2000	1.1	1.2	0.02	0.049	0.05	0.049	0.049
G-GE	5.00E-08	0.3	1000/2000	1.1	1.3	0.02	0.28	0.266	0.28	0.283
G-GE	5.00E-08	0.3	1000/2000	1.1	1.4	0.02	0.606	0.59	0.606	0.604
G-GE	5.00E-08	0.3	1000/2000	1.1	1.5	0.02	0.9	0.874	0.9	0.899
G-GE	5.00E-08	0.3	1000/2000	1.2	1	0.02	0.014	0.014	0.014	0.013
G-GE	5.00E-08	0.3	1000/2000	1.2	1.1	0.02	0.098	0.091	0.098	0.096
G-GE	5.00E-08	0.3	1000/2000	1.2	1.2	0.02	0.387	0.377	0.389	0.38
G-GE	5.00E-08	0.3	1000/2000	1.2	1.3	0.02	0.767	0.752	0.768	0.762
G-GE	5.00E-08	0.3	1000/2000	1.2	1.4	0.02	0.951	0.945	0.95	0.952
G-GE	5.00E-08	0.3	1000/2000	1.2	1.5	0.02	0.995	0.993	0.995	0.995
G-GE	5.00E-08	0.1	1000/2000	1.1	1	0.05	0	0	0	0
G-GE	5.00E-08	0.1	1000/2000	1.1	1.1	0.05	0	0	0	0
G-GE	5.00E-08	0.1	1000/2000	1.1	1.2	0.05	0.002	0.002	0.002	0.002
G-GE	5.00E-08	0.1	1000/2000	1.1	1.3	0.05	0.027	0.024	0.026	0.024
G-GE	5.00E-08	0.1	1000/2000	1.1	1.4	0.05	0.076	0.077	0.076	0.077
G-GE	5.00E-08	0.1	1000/2000	1.1	1.5	0.05	0.187	0.184	0.189	0.184
G-GE	5.00E-08	0.1	1000/2000	1.2	1	0.05	0	0.001	0	0.001
G-GE	5.00E-08	0.1	1000/2000	1.2	1.1	0.05	0.004	0.006	0.005	0.006
G-GE	5.00E-08	0.1	1000/2000	1.2	1.2	0.05	0.024	0.026	0.025	0.026
G-GE	5.00E-08	0.1	1000/2000	1.2	1.3	0.05	0.115	0.112	0.116	0.112
G-GE	5.00E-08	0.1	1000/2000	1.2	1.4	0.05	0.271	0.267	0.268	0.267
G-GE	5.00E-08	0.1	1000/2000	1.2	1.5	0.05	0.547	0.547	0.549	0.547
G-GE	5.00E-08	0.3	1000/2000	1.1	1	0.05	0	0	0	0
G-GE	5.00E-08	0.3	1000/2000	1.1	1.1	0.05	0.001	0.001	0.001	0.001
G-GE	5.00E-08	0.3	1000/2000	1.1	1.2	0.05	0.039	0.033	0.039	0.041
G-GE	5.00E-08	0.3	1000/2000	1.1	1.3	0.05	0.251	0.21	0.25	0.241
G-GE	5.00E-08	0.3	1000/2000	1.1	1.4	0.05	0.581	0.52	0.58	0.566
G-GE	5.00E-08	0.3	1000/2000	1.1	1.5	0.05	0.883	0.833	0.882	0.879
G-GE	5.00E-08	0.3	1000/2000	1.2	1	0.05	0.013	0.008	0.013	0.011
G-GE	5.00E-08	0.3	1000/2000	1.2	1.1	0.05	0.089	0.068	0.087	0.084
G-GE	5.00E-08	0.3	1000/2000	1.2	1.2	0.05	0.365	0.326	0.365	0.351
G-GE	5.00E-08	0.3	1000/2000	1.2	1.3	0.05	0.738	0.708	0.739	0.723
G-GE	5.00E-08	0.3	1000/2000	1.2	1.4	0.05	0.94	0.92	0.939	0.936
G-GE	5.00E-08	0.3	1000/2000	1.2	1.5	0.05	0.993	0.985	0.993	0.993
G-GE	5.00E-08	0.1	1000/2000	1.1	1	0.1	0	0	0	0
G-GE	5.00E-08	0.1	1000/2000	1.1	1.1	0.1	0	0	0	0
G-GE	5.00E-08	0.1	1000/2000	1.1	1.2	0.1	0.001	0.002	0.001	0.002
G-GE	5.00E-08	0.1	1000/2000	1.1	1.3	0.1	0.015	0.016	0.016	0.016
G-GE	5.00E-08	0.1	1000/2000	1.1	1.4	0.1	0.059	0.057	0.061	0.057
G-GE	5.00E-08	0.1	1000/2000	1.1	1.5	0.1	0.162	0.148	0.162	0.148
G-GE	5.00E-08	0.1	1000/2000	1.2	1	0.1	0	0	0	0
G-GE	5.00E-08	0.1	1000/2000	1.2	1.1	0.1	0.004	0.003	0.004	0.003
G-GE	5.00E-08	0.1	1000/2000	1.2	1.2	0.1	0.02	0.02	0.021	0.02
G-GE	5.00E-08	0.1	1000/2000	1.2	1.3	0.1	0.102	0.097	0.103	0.097
G-GE	5.00E-08	0.1	1000/2000	1.2	1.4	0.1	0.237	0.213	0.237	0.213
G-GE	5.00E-08	0.1	1000/2000	1.2	1.5	0.1	0.499	0.477	0.498	0.477

Test	Alpha	MAF	#case/control	beta2	beta3	Missing rate	CST	PM1	PM2	median imputation
G-GE	5.00E-08	0.3	1000/2000	1.1	1	0.1	0	0	0	0
G-GE	5.00E-08	0.3	1000/2000	1.1	1.1	0.1	0.001	0.001	0.001	0
G-GE	5.00E-08	0.3	1000/2000	1.1	1.2	0.1	0.037	0.027	0.037	0.036
G-GE	5.00E-08	0.3	1000/2000	1.1	1.3	0.1	0.212	0.16	0.212	0.196
G-GE	5.00E-08	0.3	1000/2000	1.1	1.4	0.1	0.52	0.438	0.522	0.504
G-GE	5.00E-08	0.3	1000/2000	1.1	1.5	0.1	0.838	0.735	0.837	0.824
G-GE	5.00E-08	0.3	1000/2000	1.2	1	0.1	0.011	0.006	0.01	0.004
G-GE	5.00E-08	0.3	1000/2000	1.2	1.1	0.1	0.075	0.047	0.074	0.067
G-GE	5.00E-08	0.3	1000/2000	1.2	1.2	0.1	0.315	0.248	0.314	0.302
G-GE	5.00E-08	0.3	1000/2000	1.2	1.3	0.1	0.692	0.59	0.693	0.665
G-GE	5.00E-08	0.3	1000/2000	1.2	1.4	0.1	0.918	0.854	0.917	0.903
G-GE	5.00E-08	0.3	1000/2000	1.2	1.5	0.1	0.986	0.974	0.986	0.983
G-GE	5.00E-08	0.1	1000/2000	1.1	1	0.3	0	0	0	0
G-GE	5.00E-08	0.1	1000/2000	1.1	1.1	0.3	0	0	0	0
G-GE	5.00E-08	0.1	1000/2000	1.1	1.2	0.3	0	0	0	0
G-GE	5.00E-08	0.1	1000/2000	1.1	1.3	0.3	0.006	0.006	0.006	0.006
G-GE	5.00E-08	0.1	1000/2000	1.1	1.4	0.3	0.026	0.023	0.027	0.023
G-GE	5.00E-08	0.1	1000/2000	1.1	1.5	0.3	0.063	0.051	0.06	0.051
G-GE	5.00E-08	0.1	1000/2000	1.2	1	0.3	0.001	0.001	0.001	0.001
G-GE	5.00E-08	0.1	1000/2000	1.2	1.1	0.3	0.001	0.001	0	0.001
G-GE	5.00E-08	0.1	1000/2000	1.2	1.2	0.3	0.009	0.007	0.008	0.007
G-GE	5.00E-08	0.1	1000/2000	1.2	1.3	0.3	0.039	0.025	0.036	0.025
G-GE	5.00E-08	0.1	1000/2000	1.2	1.4	0.3	0.095	0.076	0.094	0.076
G-GE	5.00E-08	0.1	1000/2000	1.2	1.5	0.3	0.273	0.219	0.272	0.219
G-GE	5.00E-08	0.3	1000/2000	1.1	1	0.3	0	0	0	0
G-GE	5.00E-08	0.3	1000/2000	1.1	1.1	0.3	0	0	0	0
G-GE	5.00E-08	0.3	1000/2000	1.1	1.2	0.3	0.011	0.006	0.012	0.004
G-GE	5.00E-08	0.3	1000/2000	1.1	1.3	0.3	0.096	0.044	0.094	0.065
G-GE	5.00E-08	0.3	1000/2000	1.1	1.4	0.3	0.297	0.135	0.297	0.246
G-GE	5.00E-08	0.3	1000/2000	1.1	1.5	0.3	0.594	0.314	0.593	0.531
G-GE	5.00E-08	0.3	1000/2000	1.2	1	0.3	0.001	0.001	0.001	0.002
G-GE	5.00E-08	0.3	1000/2000	1.2	1.1	0.3	0.029	0.014	0.031	0.017
G-GE	5.00E-08	0.3	1000/2000	1.2	1.2	0.3	0.143	0.068	0.145	0.1
G-GE	5.00E-08	0.3	1000/2000	1.2	1.3	0.3	0.416	0.217	0.416	0.34
G-GE	5.00E-08	0.3	1000/2000	1.2	1.4	0.3	0.718	0.431	0.721	0.655
G-GE	5.00E-08	0.3	1000/2000	1.2	1.5	0.3	0.926	0.694	0.928	0.886
